# Supplementary figures and images for: Death of backcountry winter-sports practitioners in avalanches – A systematic review and meta-analysis of proportion of causes of avalanche death
Source: PLOS Glob Public Health. 2025 May 30;5(5):e0004551. doi: 10.1371/journal.pgph.0004551 (PMC12124587; doi:10.1371/journal.pgph.0004551)

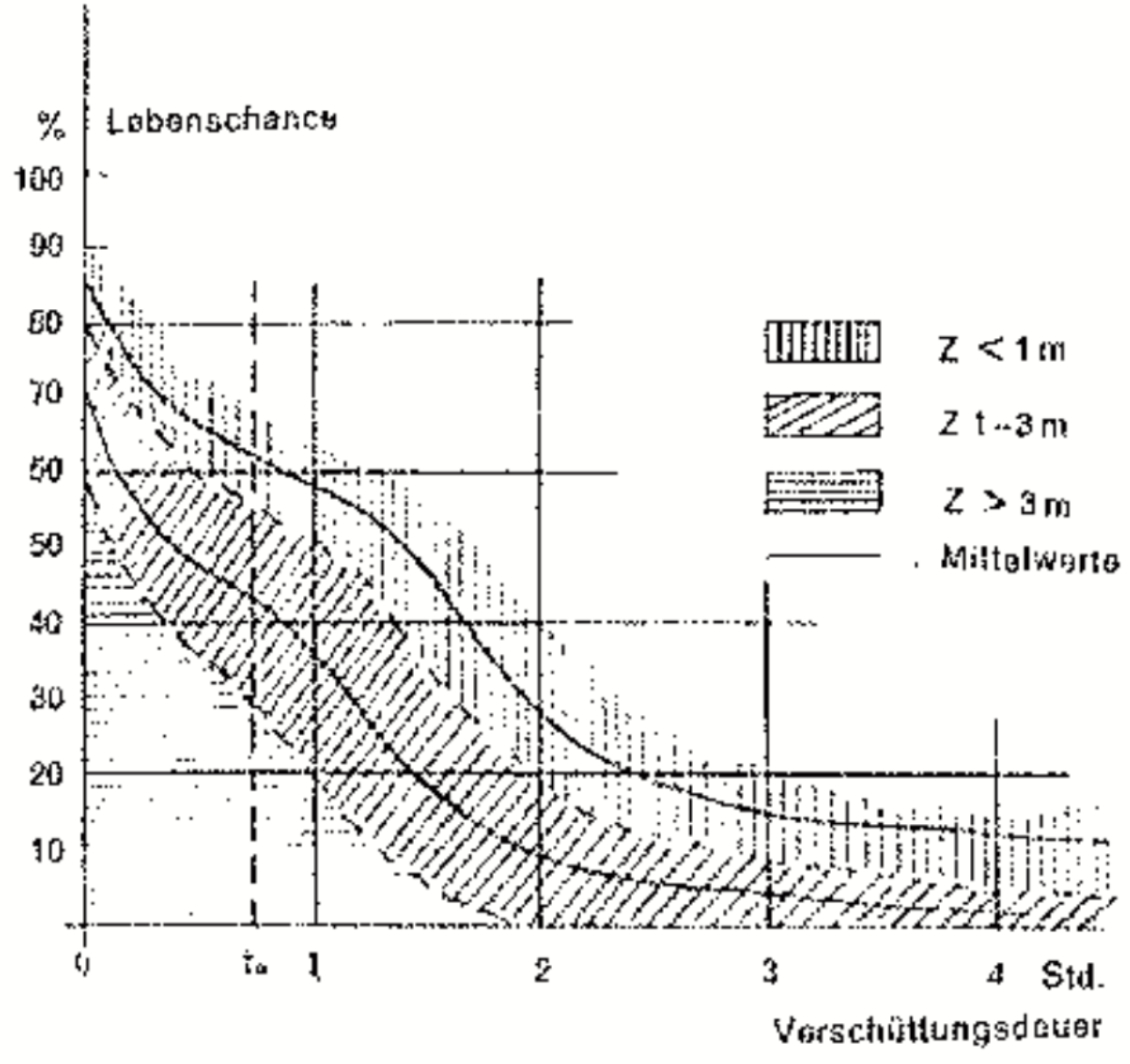

Supplement: S1 Fig — (TIF) [file pgph.0004551.s001.tif]

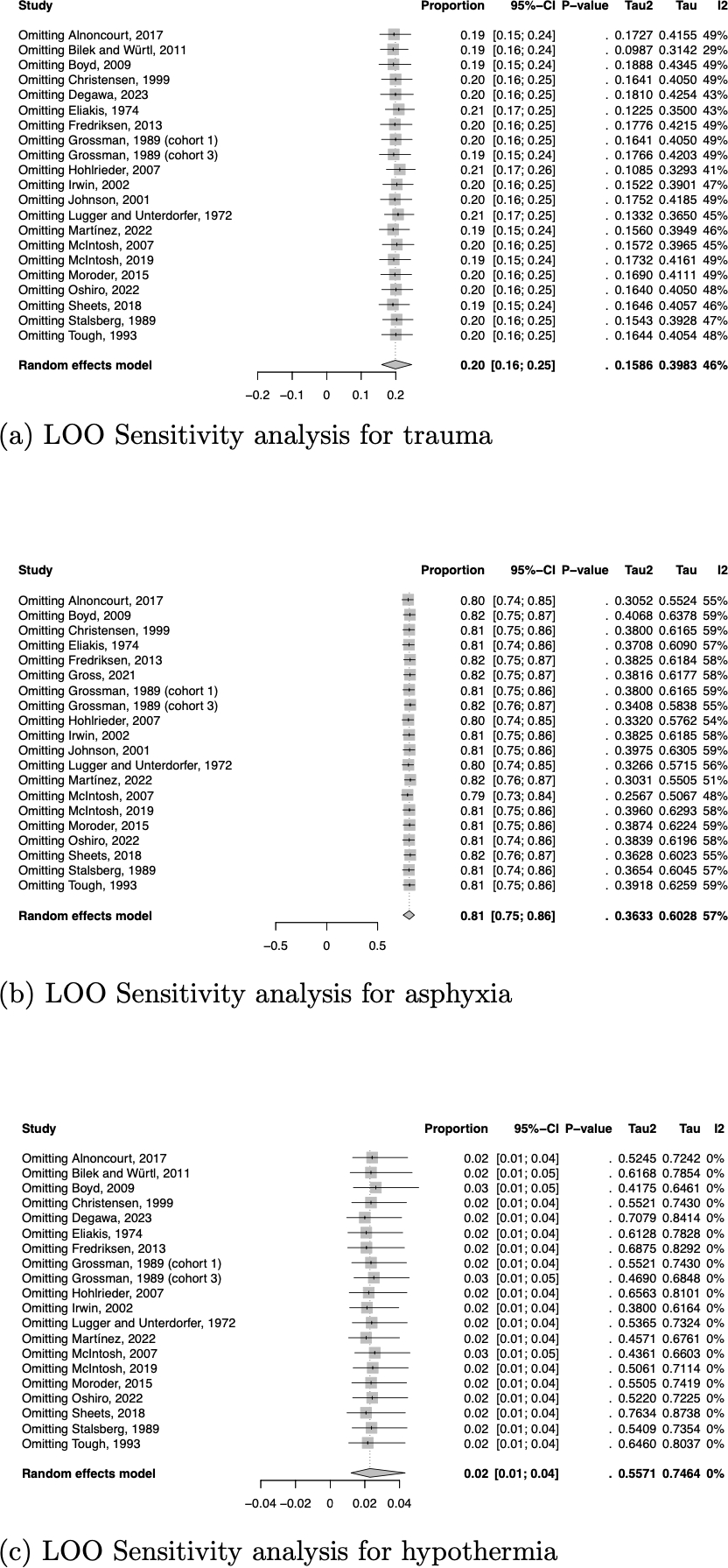

Supplement: S2 Fig — (TIF) [file pgph.0004551.s008.tif]

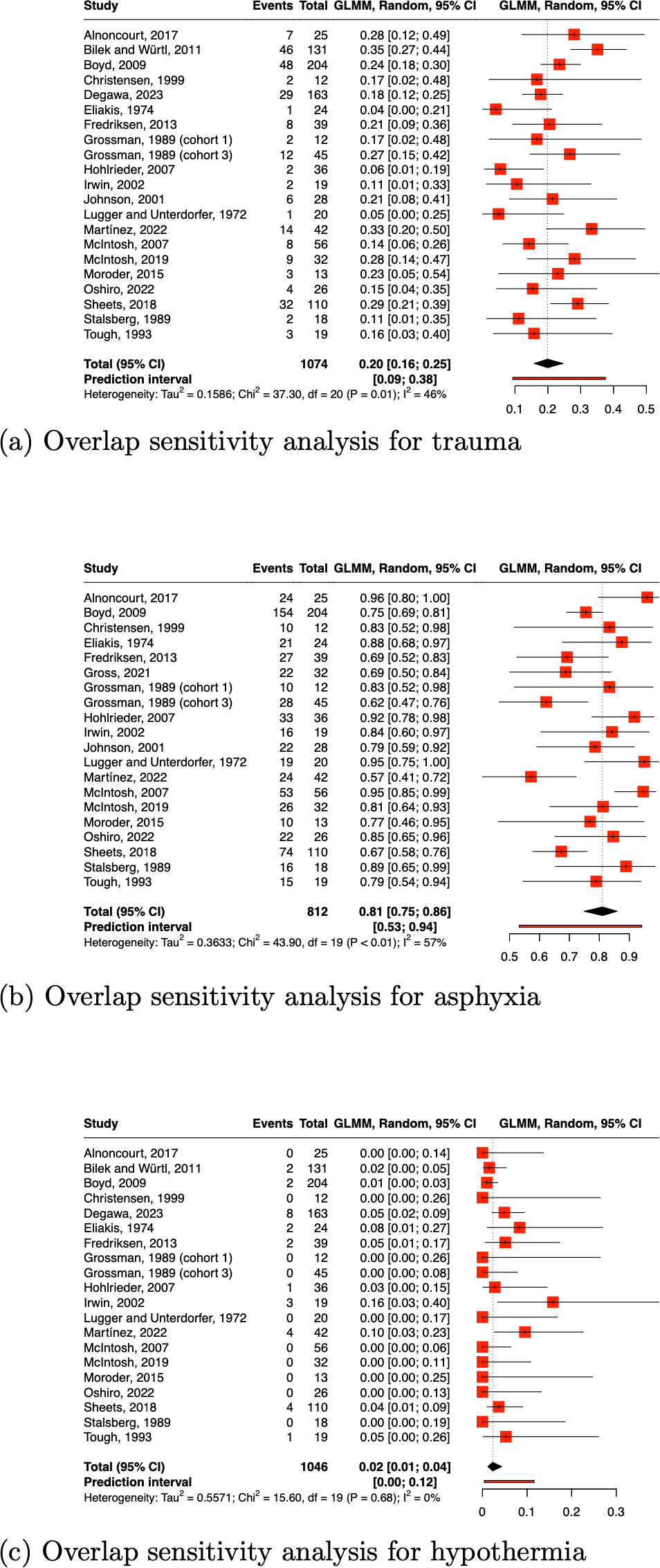

Supplement: S3 Fig — (TIF) [file pgph.0004551.s009.tif]

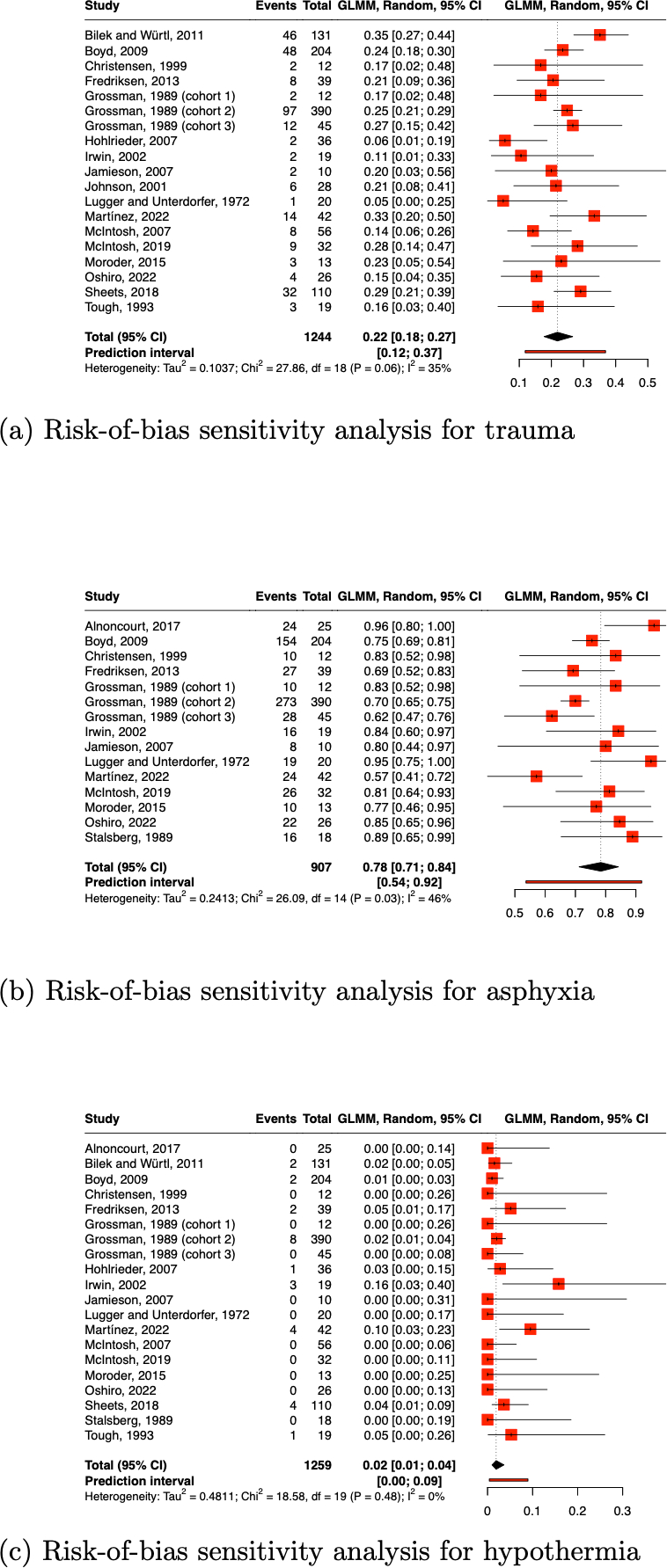

Supplement: S4 Fig — (TIF) [file pgph.0004551.s010.tif]

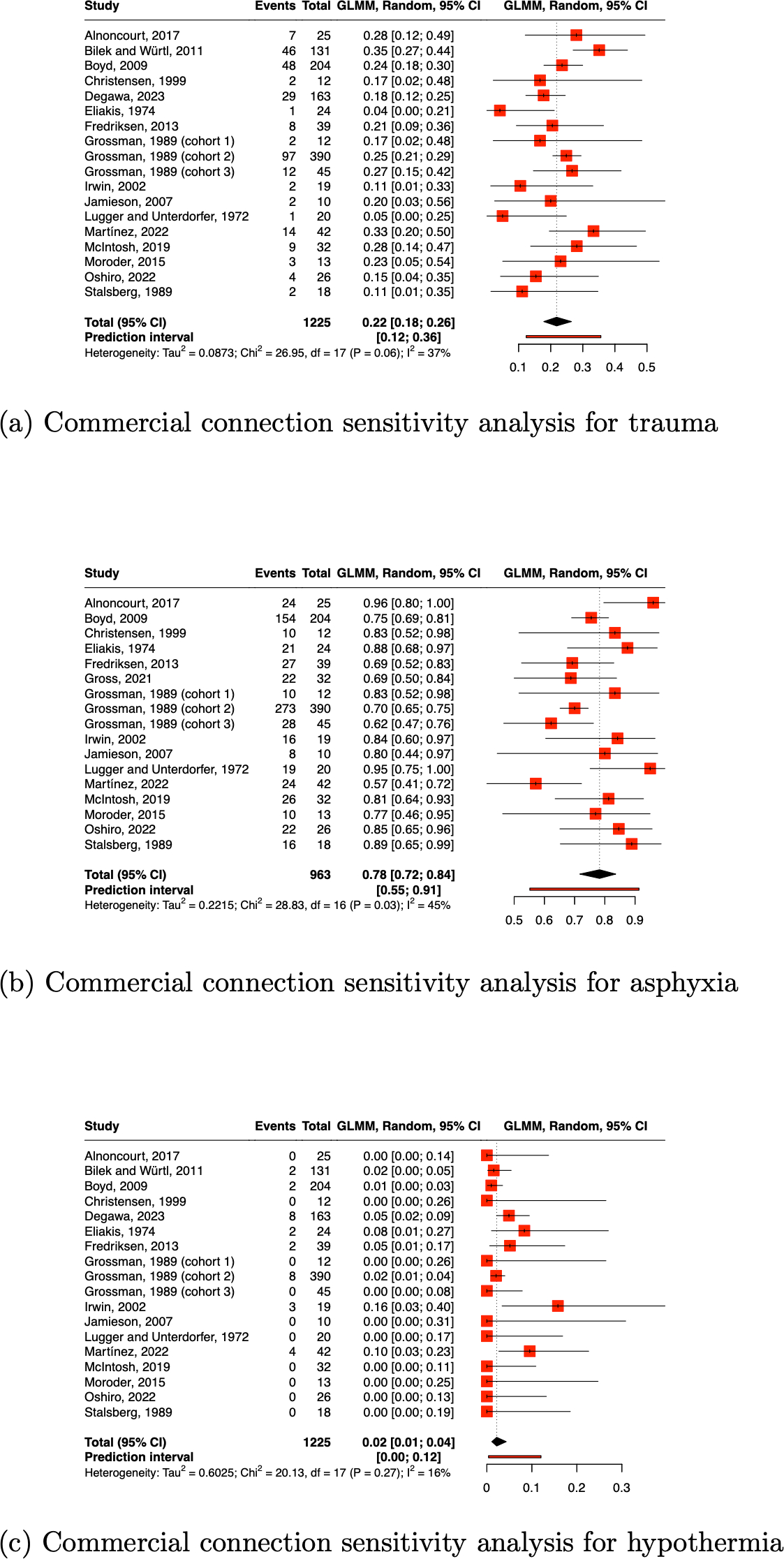

Supplement: S5 Fig — (TIF) [file pgph.0004551.s011.tif]
