# Supplementary material for: Death of backcountry winter-sports practitioners in avalanches – A systematic review and meta-analysis of proportion of causes of avalanche death
Source: PLOS Glob Public Health. 2025 May 30;5(5):e0004551. doi: 10.1371/journal.pgph.0004551 (PMC12124587; doi:10.1371/journal.pgph.0004551)
Supplement: S1 Table — (DOCX) [file pgph.0004551.s002.docx]

| **Section and Topic** | **Item #** | **Checklist item** | **Location where item is reported** |
| --- | --- | --- | --- |
| **TITLE** | | |  |
| Title | 1 | Identify the report as a systematic review. | At the beginning of the study |
| **ABSTRACT** | | |  |
| Abstract | 2 | See the PRISMA 2020 for Abstracts checklist. | At the beginning of the study |
| **INTRODUCTION** | | |  |
| Rationale | 3 | Describe the rationale for the review in the context of existing knowledge. | From the 3^rd^ paraph of introduction “Although these…” to the end of introduction |
| Objectives | 4 | Provide an explicit statement of the objective(s) or question(s) the review addresses. | In the last paragraph of introduction |
| **METHODS** | | |  |
| Eligibility criteria | 5 | Specify the inclusion and exclusion criteria for the review and how studies were grouped for the syntheses. | Section “Eligibility criteria and study selection” under “Materials and methods” |
| Information sources | 6 | Specify all databases, registers, websites, organisations, reference lists and other sources searched or consulted to identify studies. Specify the date when each source was last searched or consulted. | Section “Search strategy to identify studies” under “Materials and methods” |
| Search strategy | 7 | Present the full search strategies for all databases, registers and websites, including any filters and limits used. | S1 Table |
| Selection process | 8 | Specify the methods used to decide whether a study met the inclusion criteria of the review, including how many reviewers screened each record and each report retrieved, whether they worked independently, and if applicable, details of automation tools used in the process. | The last paragraph of section “Eligibility criteria and study selection” under “Materials and methods” |
| Data collection process | 9 | Specify the methods used to collect data from reports, including how many reviewers collected data from each report, whether they worked independently, any processes for obtaining or confirming data from study investigators, and if applicable, details of automation tools used in the process. | Section “Data extraction” under “Materials and methods” |
| Data items | 10a | List and define all outcomes for which data were sought. Specify whether all results that were compatible with each outcome domain in each study were sought (e.g. for all measures, time points, analyses), and if not, the methods used to decide which results to collect. | “Search strategy to identify studies” under “Method” (paragraph 1) |
|  | 10b | List and define all other variables for which data were sought (e.g. participant and intervention characteristics, funding sources). Describe any assumptions made about any missing or unclear information. | Section “Data extraction” under “Materials and methods” (paragraph 1) |
| Study risk of bias assessment | 11 | Specify the methods used to assess risk of bias in the included studies, including details of the tool(s) used, how many reviewers assessed each study and whether they worked independently, and if applicable, details of automation tools used in the process. | Section “Risk of bias” under “Materials and methods” |
| Effect measures | 12 | Specify for each outcome the effect measure(s) (e.g. risk ratio, mean difference) used in the synthesis or presentation of results. | Section “Data synthesis for Meta-analysis” under “Materials and methods” (sentence 2, paragraph 2) |
| Synthesis methods | 13a | Describe the processes used to decide which studies were eligible for each synthesis (e.g. tabulating the study intervention characteristics and comparing against the planned groups for each synthesis (item #5)). | Section “Data synthesis for Meta-analysis” under “Materials and methods” (sentence 2, paragraph 2) |
|  | 13b | Describe any methods required to prepare the data for presentation or synthesis, such as handling of missing summary statistics, or data conversions. | Section “Data synthesis for Meta-analysis” under “Method” (sentence 2, paragraph 2); Section “Dealing with missing data” under “Materials and methods” |
|  | 13c | Describe any methods used to tabulate or visually display results of individual studies and syntheses. | Section “Result presentation” under “Materials and methods” |
|  | 13d | Describe any methods used to synthesize results and provide a rationale for the choice(s). If meta-analysis was performed, describe the model(s), method(s) to identify the presence and extent of statistical heterogeneity, and software package(s) used. | Sections “Data synthesis for Meta-analysis”; “Assessment of heterogeneity” and “Tools” under “Materials and methods” |
|  | 13e | Describe any methods used to explore possible causes of heterogeneity among study results (e.g. subgroup analysis, meta-regression). | Section “Subgroup analysis” under “Materials and methods” |
|  | 13f | Describe any sensitivity analyses conducted to assess robustness of the synthesized results. | Section “Sensitivity analysis” under “Materials and methods” |
| Reporting bias assessment | 14 | Describe any methods used to assess risk of bias due to missing results in a synthesis (arising from reporting biases). | Section “Sensitivity analysis” under “Materials and methods” (Commercial connection) |
| Certainty assessment | 15 | Describe any methods used to assess certainty (or confidence) in the body of evidence for an outcome. | Sections “Data synthesis for Meta-analysis”; “Assessment of heterogeneity” and “Tools” under “Materials and methods” |
| **RESULTS** | | |  |
| Study selection | 16a | Describe the results of the search and selection process, from the number of records identified in the search to the number of studies included in the review, ideally using a flow diagram. | Section “Study selection” under “Results” (paragraphs 1 and 2) |
|  | 16b | Cite studies that might appear to meet the inclusion criteria, but which were excluded, and explain why they were excluded. | Section “Study selection” under “Results” (paragraph 3) |
| Study characteristics | 17 | Cite each included study and present its characteristics. | Section “Study selection” under “Results” (paragraph 4); Table 1. |
| Risk of bias in studies | 18 | Present assessments of risk of bias for each included study. | Section “Risk of bias” under “Results”; Table 1; Fig 3 |
| Results of individual studies | 19 | For all outcomes, present, for each study: (a) summary statistics for each group (where appropriate) and (b) an effect estimate and its precision (e.g. confidence/credible interval), ideally using structured tables or plots. | Table 1; Fig2A, 2B, 2C; Table 4 |
| Results of syntheses | 20a | For each synthesis, briefly summarise the characteristics and risk of bias among contributing studies. | Section “Risk of bias” under “Results” |
|  | 20b | Present results of all statistical syntheses conducted. If meta-analysis was done, present for each the summary estimate and its precision (e.g. confidence/credible interval) and measures of statistical heterogeneity. If comparing groups, describe the direction of the effect. | Table 1; Figure 2 (a, b, c); No comparing groups. |
|  | 20c | Present results of all investigations of possible causes of heterogeneity among study results. | Table 4; Table 5 |
|  | 20d | Present results of all sensitivity analyses conducted to assess the robustness of the synthesized results. | Section “Meta-analysis” under “Results” (“Sensitivity analysis” subsection) |
| Reporting biases | 21 | Present assessments of risk of bias due to missing results (arising from reporting biases) for each synthesis assessed. | Not reported |
| Certainty of evidence | 22 | Present assessments of certainty (or confidence) in the body of evidence for each outcome assessed. | Section “Meta-analysis” under “Results” (paragraph 1) |
| **DISCUSSION** | | |  |
| Discussion | 23a | Provide a general interpretation of the results in the context of other evidence. | Section “Summary of findings” under “Discussion” |
|  | 23b | Discuss any limitations of the evidence included in the review. | Section “Limitations of the study” under “Discussion” (paragraphs 2 and 3) |
|  | 23c | Discuss any limitations of the review processes used. | Section “Limitations of the study” under “Discussion” (paragraphs 1) |
|  | 23d | Discuss implications of the results for practice, policy, and future research. | Section “Implication” under “Discussion” |
| **OTHER INFORMATION** | | |  |
| Registration and protocol | 24a | Provide registration information for the review, including register name and registration number, or state that the review was not registered. | “Materials and methods” section (paragraph 1) |
|  | 24b | Indicate where the review protocol can be accessed, or state that a protocol was not prepared. | “Materials and methods” section (paragraph 1) |
|  | 24c | Describe and explain any amendments to information provided at registration or in the protocol. | Section “risk of bias” under “Materials and methods” (last paragraph); Section “Assessment of heterogeneity” under “Materials and methods” (last paragraph); section “Commercial connections” (last sentence) |
| Support | 25 | Describe sources of financial or non-financial support for the review, and the role of the funders or sponsors in the review. | Section “Funding” |
| Competing interests | 26 | Declare any competing interests of review authors. | Section “Conflict of interests” |
| Availability of data, code and other materials | 27 | Report which of the following are publicly available and where they can be found: template data collection forms; data extracted from included studies; data used for all analyses; analytic code; any other materials used in the review. | Data extracted from included studies are made open in OSF(https://osf.io/br8m5). Data collection form are in S4 Table, and codes are made open in OSF (https://osf.io/4c5ah). |

*From:*  Page MJ, McKenzie JE, Bossuyt PM, Boutron I, Hoffmann TC, Mulrow CD, et al. The PRISMA 2020 statement: an updated guideline for reporting systematic reviews. BMJ 2021;372:n71. doi: 10.1136/bmj.n71
