# Supplementary material for: Death of backcountry winter-sports practitioners in avalanches – A systematic review and meta-analysis of proportion of causes of avalanche death
Source: PLOS Glob Public Health. 2025 May 30;5(5):e0004551. doi: 10.1371/journal.pgph.0004551 (PMC12124587; doi:10.1371/journal.pgph.0004551)
Supplement: S2 Table — (PDF) [file pgph.0004551.s003.pdf]

S2 Table. Searching strategy

Ovid MEDLINE	1 exp avalanche/ ; 2 avalanche*.tw.; 3 “snow burial*”.tw.; 4 “snow immers*”.tw.; 5 casualty.tw. ; 6 mortality.tw. ; 7 fatal*.tw. ; 8 death.tw. ; 9 trauma.tw. ; 10 injur*.tw. ; 11 survival.tw. ; 12 snow*.tw. ; 13 ski*.tw. ; 14 mountain*.tw. ; 15 sled*.tw. ; 16 climb*.tw. ; 17 winter.tw. ; 18 sport*.tw. ; 19 activit*.tw. ; 20 pastime.tw. ; 21 recreation.tw. ; 22 or/1-4; 23 or/5-11 ; 24 22 and 23 ; 25 or/12-16 ; 26 or/18-21 ; 27 17 and 26 ; 28 25 or 27 ; 29 24 and 28
EMBASE	1 avalanche/ exp ; 2 avalanche*:ab,ti; 3 ‘snow burial*’:ab,ti; 4 ‘snow immers*’:ab,ti; 5 ‘casualty’:ab,ti ; 6 ‘mortality’:ab,ti ; 7 ‘fatal*’:ab,ti; 8 ‘death’:ab,ti; 9 ‘trauma’:ab,ti; 10 ‘injur*’:ab,ti; 11 ‘survival’:ab,ti; 12 ‘snow*’:ab,ti; 13 ‘ski*’:ab,ti; 14 ‘mountain*’:ab,ti; 15 ‘sled*’:ab,ti; 16 ‘climb*’:ab,ti; 17 ‘winter’:ab,ti; 18 ‘sport*’:ab,ti; 19 ‘activit*’:ab,ti; 20 ‘pastime’:ab,ti; 21 ‘recreation’:ab,ti; 22 1 OR 2 OR 3 OR 4; 23 5 OR 6 OR 7 OR 8 OR 9 OR 10 OR 11; 24 22 AND 23; 25 12 OR 13 OR 14 OR 15 OR 16 ; 26 18 OR 19 OR 20 OR 21; 27 17 and 26 ; 28 25 or 27 ; 29 24 and 28
Cochrane	1 MeSH descriptor Avalanche explode all trees; 2 avalanche*:ab,ti; 3 “snow burial*”:ab,ti; 4 “snow immers*”:ab,ti; 5 casualty:ab,ti ; 6 mortality:ab,ti ; 7 fatal*:ab,ti; 8 death:ab,ti; 9 trauma:ab,ti; 10 injur*:ab,ti; 11 survival:ab,ti; 12 snow*:ab,ti; 13 ski*:ab,ti; 14 mountain*:ab,ti; 15 sled*:ab,ti; 16 climb*:ab,ti; 17 winter:ab,ti; 18 sport*:ab,ti; 19 activit*:ab,ti; 20 pastime:ab,ti; 21 recreation:ab,ti; 22 or/1-4; 23 or/5-11 ; 24 22 and 23 ; 25 or/12-16 ; 26 or/18-21 ; 27 17 and 26 ; 28 25 or 27; 29 24 and 28

Web of Science	1 TS=(avalanche*); 2 TS=(snow burial); 3 TS=(snow immers*); 4 TS=(casualty) ; 5 TS=(mortality); 6 TS=(fatal*); 7 TS=(death?); 8 TS=(trauma); 9 TS=(injur*); 10 TS=(survival); 11 TS =(snow*); 12 TS =(ski*); 13 TS =(mountain*); 14 TS =(sled*); 15 TS =(climb*); 16 TS =(winter); 17 TS =(sport*); 18 TS =(activit*); 19 TS =(pastime); 20 TS =(recreation); 21 ((1 OR 2 OR 3) AND (4 OR 5 OR 6 OR 7 OR 8 OR 9 OR 10)) AND ((11 OR 12 OR 13 OR 14 OR 15) OR (16 AND (17 OR 18 OR 19 OR 20)))
Academic Search Complete (EBSCO)	S1 TI(avalanche*) OR AB(avalanche*); S2 TI(snow burial*) OR AB(snow burial*); S3 TI(snow immers*) OR AB(snow immers*); S4 TI(casualty) OR AB(casualty); S5 TI(mortality) OR AB(mortality); S6 TI(fatal*) OR AB(fatal*); S7 TI(death?) OR AB(death?); S8 TI(trauma) OR AB(trauma); S9 TI(injur*) OR AB(injur*); S10 TI(survival) OR AB(survival); S11 TI(snow*) OR AB(snow*); S12 TI(ski*) OR AB(ski*); S13 TI(mountain*) OR AB(mountain*); S14 TI(sled*) OR AB(sled*); S15 TI(climb*) OR AB(climb*); S16 TI(winter) OR AB(winter); S17 TI(sport*) OR AB(sport*); S18 TI(activit*) OR AB(activit*) ; S19 TI(pastime) OR AB(pastime); S20 TI(recreation) OR AB(recreation); S21 ((S1 OR S2 OR S3) AND (S4 OR S5 OR S6 OR S7 OR S8 OR S9 OR S10)) AND ((S11 OR S12 OR S13 OR S14 OR S15) OR (S16 AND (S17 OR S18 OR S19 OR S20)))

SPORTdicus (EBSCO)	S1 TI(avalanche*) OR AB(avalanche*); S2 TI(snow burial*) OR AB(snow burial*); S3 TI(snow immers*) OR AB(snow immers*); S4 TI(casualty) OR AB(casualty); S5 TI(mortality) OR AB(mortality); S6 TI(fatal*) OR AB(fatal*); S7 TI(death?) OR AB(death?); S8 TI(trauma) OR AB(trauma); S9 TI(injur*) OR AB(injur*); S10 TI(survival) OR AB(survival) ; S11 TI(snow*) OR AB(snow*); S12 TI(ski*) OR AB(ski*); S13 TI(mountain*) OR AB(mountain*); S14 TI(sled*) OR AB(sled*); S15 TI(climb*) OR AB(climb*); S16 TI(winter) OR AB(winter); S17 TI(sport*) OR AB(sport*); S18 TI(activit*) OR AB(activit*) ; S19 TI(pastime) OR AB(pastime); S20 TI(recreation) OR AB(recreation); S21 ((S1 OR S2 OR S3) AND (S4 OR S5 OR S6 OR S7 OR S8 OR S9 OR S10)) AND ((S11 OR S12 OR S13 OR S14 OR S15) OR (S16 AND (S17 OR S18 OR S19 OR S20)))
Eric (Proquest)	1 AB,TI(avalanche*); 2 AB,TI(snow burial*); 3 AB,TI(snow immers*); 4 AB,TI(casualty); 5 AB,TI(mortality) ; 6 AB,TI(fatal*); 7 AB,TI(death?); 8 AB,TI(trauma); 9 AB,TI(injur*); 10 AB,TI(survival); 11 AB,TI(snow*); 12 AB,TI(ski*); 13 AB,TI(mountain*); 14 AB,TI(sled*); 15 AB,TI(climb*); 16 AB,TI(winter); 17 AB,TI(sport*); 18 AB,TI(activit*); 19 AB,TI(pastime); 20 AB,TI(recreation); 21 ((S1 OR S2 OR S3) AND (S4 OR S5 OR S6 OR S7 OR S8 OR S9 OR S10)) AND ((S11 OR S12 OR S13 OR S14 OR S15) OR (S16 AND (S17 OR S18 OR S19 OR S20)))

Scopus	1 TITLE-ABS(avalanche*); 2 TITLE-ABS (snow burial*); 3 TITLE-ABS (snow immers*); 4 TITLE-ABS (casualty); 5 TITLE-ABS (mortality) ; 6 TITLE-ABS (fatal*); 7 TITLE-ABS (death?); 8 TITLE-ABS (trauma); 9 TITLE-ABS (injur*); 10 TITLE-ABS (survival); 11 TITLE-ABS (snow*); 12 TITLE-ABS (ski*); 13 TITLE-ABS (mountain*); 14 TITLE-ABS (sled*); 15 TITLE-ABS (climb*); 16 TITLE-ABS (winter); 17 TITLE-ABS (sport*); 18 TITLE-ABS (activit*); 19 TITLE-ABS (pastime); 20 TITLE-ABS (recreation); 21 ((1 OR 2 OR 3) AND (4 OR 5 OR 6 OR 7 OR 8 OR 9 OR 10)) AND ((11 OR 12 OR 13 OR 14 OR 15) OR (16 AND (17 OR 18 OR 19 OR 20)))
Safetylit	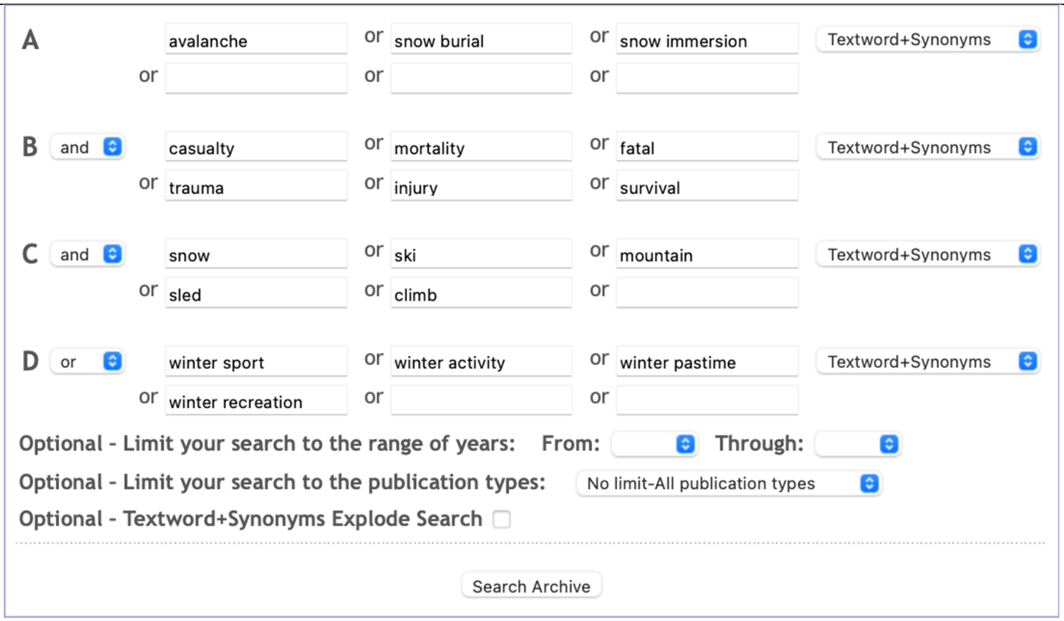

ISSW	Since the database has a very focused pool of studies relevant to avalanche, we searched for all the studies containing either "trauma", "asphyxia" or "hypothermia" in the full text
