# Supplementary material for: Death of backcountry winter-sports practitioners in avalanches – A systematic review and meta-analysis of proportion of causes of avalanche death
Source: PLOS Glob Public Health. 2025 May 30;5(5):e0004551. doi: 10.1371/journal.pgph.0004551 (PMC12124587; doi:10.1371/journal.pgph.0004551)
Supplement: S1 Text — (PDF) [file pgph.0004551.s004.pdf]

S1 Text. Risk of bias tool and summary of key items

Name of author(s): ___________________________ **Year of publication:** ______________

Name of paper/study: ____

This tool is designed to assess the risk of bias in population-based prevalence studies. Please read the additional notes for each item when initially using the tool. *Note: If there is insufficient information in the article to permit a judgement for a particular item, please answer **No (HIGH RISK)** for that particular item.*

External Validity

1. **Was the study's target population a close representation of the national/regional population in relation to relevant variables, e.g., age, sex, occupation?**

   Yes (LOW RISK): The study's target population was a close representation of the national/regional population

   No (HIGH RISK): The study's target population was clearly NOT representative of the national/regional population

   Examples:

   - Historical records from the Utha avalanche fatalities for the studied period were reviewed. The answer is: **Yes (LOW RISK)**.
   - All avalanche victims who at the forensic institute of Grenoble University Hospital for the studied period were reviewed. The answer is: **No (HIGH RISK, only representative of the hospital, unless it is the only hopital serving the region)**.

2. **Was the sampling frame a true or close representation of the target population?**

   Yes (LOW RISK): The sampling frame was a true or close representation of the target population.

No (HIGH RISK): The sampling frame was NOT a true or close representation of the target population.

Examples:

- The database includes data from most Japanese prefectures. The answer is: **Yes (LOW RISK)**.
- The case files of the Pierce County Medical Examiner's Office were reviewed for all fatalities. The answer is: **Yes (LOW RISK)**.
- We analyzed avalanche victims delivered to the emergency department of our hospital and failed to be resuscitated. The answer is: **No (HIGH RISK, all fatalities who were dead on-site can be missed)**.

3. **Was some form of random selection used to select the sample, OR, was a census undertaken?**

   Yes (LOW RISK):A census was undertaken, OR, some form of random selection was used to select the sample (e.g. simple random sampling, stratified random sampling, cluster sampling, systematic sampling).

   No (HIGH RISK):A census was NOT undertaken, AND some form of random selection was NOT used to select the sample.

   Examples:

   - All death cases were included. The answer is: **Yes (LOW RISK)**.
   - A random sample of all death cases were included. The answer is: **Yes (LOW RISK)**.
   - We collected such reports from police headquarters within all prefectures that had over 10 mountain death cases. The answer is: **No (HIGH RISK, selecting over 10 death accident is not random sampling)**.

4. **Was the likelihood of non-response bias minimal?**

   Yes (LOW RISK): The response rate for the study was $\geq$ 75%, OR, an analysis was performed that showed no significant difference in relevant demographic characteristics between responders and non-responders

No (HIGH RISK): The response rate was $< 75\%$, and if any analysis comparing responders and non-responders was done, it showed a significant difference in relevant demographic characteristics between responders and non-responders.

Note for the current study: non-response is impossible in our case, considering the researched targets are bodies of avalanche fatalities, and any improper missing of values due to study design will be rated under "random select".

Examples:

None

Internal Validity

5. **Were data collected directly from the subjects (as opposed to a proxy)?**

Yes (LOW RISK): All forensic diagnosis used in the study were made by physicians, pathologists, or autopsy practitioners who collected information directly from the subjects.

No (HIGH RISK): Forensic diagnosis used in the study were made by people without direct access to the subjects.

Examples:

- The autopsy diagnosis used for analysis were established by collecting the results of the autopsy reports made by pathologists. The answer is: **Yes (LOW RISK)**.
- Authors determined autopsy diagnosis on accident descriptions. The answer is: **No (HIGH RISK)**.

6. **Was an acceptable case definition used in the study?**

Yes (LOW RISK): An acceptable definition of trauma, asphyxia and hypothermia, and any other diagnosis used in the study was reported. between responders and non-responders

No (HIGH RISK): An acceptable definition of trauma, asphyxia and hypothermia, and any other diagnosis used in the study was NOT reported.

Examples:

- Trauma refers to physical injuries inflicted on the body due to the mechanical forces associated with the avalanche; Asphyxia refers to...; Hypothermia refers to... The answer is: **Yes (LOW RISK)**.
- The authors gave no definition of the causes of death used in the study was given. The answer is: **No (HIGH RISK)**.

7. **Was the study instrument that measured the parameter of interest (e.g. prevalence of low back pain) shown to have reliability and validity (if necessary)?**

Yes (LOW RISK): The category of cause of avalanche death includes at least trauma, asphyxia and hypothermia.

No (HIGH RISK): The category of cause of avalanche death includes none or some of trauma, asphyxia and hypothermia.

Examples:

- The authors used three types of cause including trauma, asphyxia and hypothermia. The answer is: **Yes (LOW RISK)**.
- The authors used trauma and brain death. The answer is: **No (HIGH RISK)**.
- The authors used trauma and asphyxia. The answer is: **No (HIGH RISK)**.

8. **Was the same mode of data collection used for all subjects?**

Yes (LOW RISK): All cases received internal autopsy, a sequential adoption of external and internal autopsy, or either external or internal autopsy according to diagnostic need for each case.

No (HIGH RISK): All or some of the cases received external autopsy only, and the lack of internal autopsy is not the result that the cause of death is so obvious (based on medical opinion) that an internal autopsy is not needed.

Examples:

- All fatalities received internal autopsy unless the cause of the death is obvious such as decapitation. The answer is: **Yes (LOW RISK)**.
- Five of 42 victims did not receive internal autopsy, due to the refusal of their family members. The answer is: **Yes (HIGH RISK)**.
- Internal autopsies were performed in 30 of the remaining 30 cases (. The answer is: **No (HIGH RISK)**.

9. **Was the length of the shortest prevalence period for the parameter of interest appropriate?**

   Yes (LOW RISK): The shortest prevalence period for the parameter of interest was appropriate (one year in our case).

   No (HIGH RISK): **No (HIGH RISK)**: The shortest prevalence period for the parameter of interest was not appropriate (less than one year in our case)

Examples:

- All cases during the period of 1980–1991 were included. The answer is: **Yes (LOW RISK)**.
- All cases during November 2023 were included. The answer is: **No (HIGH RISK)**.

10. **Were the numerator(s) and denominator(s) for the parameter of interest appropriate?**

    Yes (LOW RISK): The paper presented appropriate numerator(s) AND denominator(s) for the parameter of interest.

    No (HIGH RISK): The paper did present numerator(s) AND denominator(s) for the parameter of interest but one or more of these were inappropriate.

Examples:

- The authors used the number of all fatalities during the studied period as denominator and the number of all cases with a particular casue of death diagnosis as nominator, without any missing cause of death or non-investigated case. The answer is: **Yes (LOW RISK)**.
- There were cases with unclear causes of death. The answer is: **No (HIGH RISK)**.

11. **Summary item on the overall risk of study bias**

    LOW RISK OF BIAS: Further research is very unlikely to change our confidence in the estimate.

    MODERATE RISK OF BIAS: Further research is likely to have an important impact on our confidence in the estimate and may change the estimate.

    HIGH RISK OF BIAS: Further research is very likely to have an important impact on our confidence in the estimate and is likely to change the estimate.

Details on items most often rated as 'high risk of bias'"

Case definition (F) This item is defined as "*Was an acceptable case definition used in the study?*". In our meta-analysis, a case definition should be the definition of trauma, asphyxia, and hypothermia as cause of death and clarification of the forensic diagnostic criterion adopted to conclude the cause. In 24 cohorts for our meta-analysis, 15 did not disclose the forensic criterion, substantially increasing the risk of bias of the meta-analysis. See figure 2(d):item F (case definition).

Mode of data collection(H) This item is defined as "Was the same mode of data collection used for all subjects?". In our meta-analysis, the mode of data collection of a PCAD study can be inconsistent when autopsy was only performed on some cases or when the diagnosis for some cases went missing. The cohorts that had some non-autopsied cases, but clarified a reason for which no bias was likely to be introduced, were still considered as low risk of bias. For example, although autopsy is considered the gold standard for determining death causes, it is legitimate for a medical examiner to

not perform it on a case with certain signs for a particular cause, such as decapitation. Sixteen cohorts have high risk of bias on this item. See figure 2(d):item H (mode of data collection).

Numerators and denominator(J) This item is defined as "Were the numerator(s) and denominator(s) for the parameter of interest appropriate?" In our meta-analysis, denominator can be inappropriate when there is some unknown causes included or excluded due to non-random missing cases. For example, denial of autopsy by the fatalities' family member is considered as random missing case, leading to a low risk rating. Among the nine cohorts rated as high risk in this item, one [1] excluded all non-autopsied cases without clarifying the reason (the causes for their death was not disclosed); one [2] did not report if there are any missing cases (according to the instruction of the risk-of-bias tool, being unclear should be rated as high risk); one did not report the number of fatalities for some causes of death (numerator missing) [3]; six [4–9] reported some cases with missing or unidentified cause of death but did not give information for randomness evaluation.

References

1. d'Alnoncourt S. Étude des mécanismes de décès des victimes d'avalanche dans les Alpes françaises, à partir d'une série de 25 observations autopsiques, radiologiques et anatomopathologiques; 2017.

2. Eliakis E. La mort violente par avalanche. Mise au point medico-legale. Medicine Legale et Dommage Corporel. 1974; 7:83–87.

3. Gross M, Jackowski C, Schön. Fatalities associated with ski touring and freeriding: A retrospective analysis from 2001 to 2019. Forensic Science International: Reports. 2021; 4:100239.

4. Irwin D, MacQueen W, Owens IF. Avalanche accidents in Aotearoa-New Zealand. New Zealand Mountain Safety Council; 2002.

5. Bruce Jamieson PH, Gauthier D. Avalanche Accidents in Canada, Volume 5: 1996-2007. Canadian Avalanche Association; 2010.

6. Martínez ÍS, Ayala M, Casadesús JM, Domènech GM, Trullàs JC, Mariño RB. Main causes of accidental deaths due to avalanches in the Catalan Pyrenees: a review of 50 years. Emergencias. 2022; 34(6):483–485.

7. Moroder L, Mair B, Brugger H, Voelckel W, Mair P. Outcome of avalanche victims with out-of-hospital cardiac arrest. Resuscitation. 2015; 89:114–118.

8. Sheets A, Wang D, Logan S, Atkins D. Causes of Death Among Avalanche Fatalities in Colorado: A 21-Year Review. Wilderness & Environmental Medicine. 2018; 29(3):325–329.

9. Fieler J. North-Norwegian avalanche victims: a retrospective observational study. Universitetet i Tromsø; 2013.
