# Supplementary material for: Death of backcountry winter-sports practitioners in avalanches – A systematic review and meta-analysis of proportion of causes of avalanche death
Source: PLOS Glob Public Health. 2025 May 30;5(5):e0004551. doi: 10.1371/journal.pgph.0004551 (PMC12124587; doi:10.1371/journal.pgph.0004551)
Supplement: S3 Table — (PDF) [file pgph.0004551.s005.pdf]

S3 Table. Author contacting effort, dates and response

Study	Author	Reason for contact	Information to procure	Date and Response	Decision
Characteristics of fatal accidents due to exogenous causes at ski resorts in Japan over the past 13 years: a retrospective descriptive study	Tanaka, Shota and Sagisaka, Ryo and Nakagawa, Koshi and Tanaka, Hideharu	The authors reported causes of avalanche death in Japanese skiing resorts without giving specific number. The authors implicitly indicated none of the cause was due to asphyxia but it was unclear.	Ask to confirm if none died due to asphyxia; Ask to share the numbers for trauma and hypothermia non-survivors.	Contacted on March 29th, 2024; Response received on April 17th, 2024; Response includes description for each case	Included for qualitative systematic review only
Fatalities associated with ski touring and freeriding: A retrospective analysis from 2001 to 2019	Gross, M. and Jackowski, C. and Schön, C. A.	The authors reported the number of all non-survivors and the asphyxia victims among them, but numbers for other causes of death were unclear.	Ask to share the numbers for trauma and hypothermia non-survivors.	Contacted on March 29th, 2024. None response achieved	Included for meta-analysis of asphyxia and systematic review

Pathologie des ensevelis	Par A. Lapras	This study had inconsistencies between the reported number of fatalities (n=41) and the numbers summed across all fatalities' death causes (n=31, the categories included "unclear" cause of death), possibly indicating a typo and thus bias against the cause with mistakenly under-reported number.	We contacted the authors for procuring the diagnosis of the excluded cases	We did not find contact information of the author	Included for qualitative systematic review
