# Supplementary material for: Death of backcountry winter-sports practitioners in avalanches – A systematic review and meta-analysis of proportion of causes of avalanche death
Source: PLOS Glob Public Health. 2025 May 30;5(5):e0004551. doi: 10.1371/journal.pgph.0004551 (PMC12124587; doi:10.1371/journal.pgph.0004551)
Supplement: S4 Table — (PDF) [file pgph.0004551.s006.pdf]

## Appendix. Data screening record.

**This table records inclusion and exclusion decisions (with reasons for exclusion) for 1031 studies/registries (750 unique ones from database search and 281 unique ones from reference screening and expert consultation); Reasons to exclude correspond to Fig 1 in the paper**

| Index | Publication Year | Author | Title | If included | Reason for exclusion | URL for grey included paper |
|---|---|---|---|---|---|---|
| 1 | 2019 | 张俊才; 周保; 曹小岩; 魏赛拉加 | 阿尼玛卿山冰崩链生灾害基本特征分析 | No | Excluded at title/ abstract screening | |
| 2 | 2008 | Aboodi, Ron; Borer, Adi; Enoch, David | DEONTOLOGY, INDIVIDUALISM, AND UNCERTAINIY: A REPLY TO JACKSON AND SMITH | No | Excluded at title/ abstract screening | |
| 3 | 2015 | Abrahamsen, Håkon B. | A remotely piloted aircraft system in major incident management: concept and pilot, feasibility study | No | Excluded at title/ abstract screening | |
| 4 | 2006 | Accident statistics 1950–2007. | Boulder (CO): Colorado Avalanche Information Center; 2006 | No | Data already covered | |
| 5 | 2020 | Adam Stich, Jacob Blanco | Backcountry Triggered Avalanches: A Summary of Risk Factors, Causes of Death, and Wilderness Medical Management | No | All relevant statistics were cited elsewhere | |
| 6 | 1989 | Addiss DG, Baker SP" | Mountaineering and rock-climbing injuries in US National Parks | No | Irrelevant title/ abstract (reports from other methods) | |
| 7 | 1990 | age CE, Atkins D, Shockley LW | Avalanche deaths in the United States: a 45-year analysis. | No | No relevant data (full text examined, papers through other methods) | |
| 8 | 2015 | Ágústsdóttir, A. M. | Ecosystem approach for natural hazard mitigation of volcanic tephra in Iceland: building resilience and sustainability | No | Excluded at title/ abstract screening | |
| 9 | 2008 | Alain, Duclos; Julie, Laffon; Jerome, Chapelle | Avalanche Data: To Share or Not to Share? | No | Excluded at title/ abstract screening | |
| 10 | 2009 | Alberini, A.; Leiter, A. M.; Rheinberger, C. M.; McCormick, C.; Mizrahi, A. | Avaluation: Estimating the economic value of the avalanche bulletin | No | Excluded at title/ abstract screening | |
| 11 | 1988 | Alberta Recreation and Parks. | Estimates of provincial participation in recreational activities. | | Irrelevant title/ abstract (reports from other methods) | |
| 12 | 1964 | Allam, Bahgat F. | Studies on the function of the human adrenal cortex in obesity and Cushing's syndrome | No | Excluded at title/ abstract screening | |

| **Appendix. Data screening record.** | | | | | | |
|---|---|---|---|---|---|---|
| **This table records inclusion and exclusion decisions (with reasons for exclusion) for 1031 studies/registries (750 unique ones from database search and 281 unique ones from reference screening and expert consultation); Reasons to exclude correspond to Fig 1 in the paper** | | | | | | |
| **Index** | **Publication Year** | **Author** | **Title** | **If included** | **Reason for exclusion** | **URL for grey included paper** |
| **13** | **2017** | Alnoncourt, HAL | Étude des mécanismes de décès des victimes d'avalanche dans les Alpes françaises, à partir d'une série de 25 observations autopsiques, radiologiques et anatomopathologiques | Yes, for meta-analysis and systematic review | NA, since included | |
| **14** | **1982** | Althaus U, Aeberhard P, Schfipbach P, Nachbfir BH, Mfihlemann W | Management of profound accidental hypothermia with cardiorespiratory arrest. | No | Irrelevant title/ abstract (reports from other methods) | |
| **15** | **1991** | Ambach E | Fatal accidents on glaciers: forensic, criminological and glaciological conclusions | No | No relevant data (full text examined, papers through other methods) | |
| **16** | **2013** | Amdur, Neil | Extreme Grief | No | Excluded at title/ abstract screening | |
| **17** | **2020** | Amoroso, M. M.; Blazina, A. P. | Disturbance History and Dynamics of an Old-Growth Nothofagus Forest in Southern Patagonia | No | Excluded at title/ abstract screening | |
| **18** | **1996** | Anderson, Kristin | A man-made avalanche kills | No | Excluded at title/ abstract screening | |
| **19** | **2014** | Andruszkow, H.; Hildebrand, F. | Accidental Hypothermia | No | Excluded at title/ abstract screening | |
| **20** | | ANENA | Association Nationale pour l'Étude de la Neige et des Avalanches (ANENA)(anena.org) | No | Irrelevant title/ abstract (reports from other methods) | |
| **21** | **2018** | Annie, Frank H.; Bates, Mark C.; Uejio, Chris K.; Bhagat, Abhishek; Kochar, Tanureet; Embrey, Sarah | The Impact of the Drug Epidemic on the Incidence of Sepsis in West Virginia | No | Excluded at title/ abstract screening | |
| **22** | **2010** | Arbellay, E.; Stoffel, M.; Bollschweiler, M. | Dendrogeomorphic reconstruction of past debris-flow activity using injured broad-leaved trees | No | Excluded at title/ abstract screening | |
| **23** | **2012** | Arbellay, Estelle; Corona, Christophe; Stoffel, Markus; Fonti, Patrick; Decaulne, Armelle | Defining an Adequate Sample of Earlywood Vessels for Retrospective Injury Detection in Diffuse-Porous Species | No | Excluded at title/ abstract screening | |
| **24** | **2013** | Arbellay, Estelle; Stoffel, Markus; Decaulne, Armelle | Dating of snow avalanches by means of wound-induced vessel anomalies in sub-arctic Betula pubescens | No | Excluded at title/ abstract screening | |

**Appendix. Data screening record.**

**This table records inclusion and exclusion decisions (with reasons for exclusion) for 1031 studies/registries (750 unique ones from database search and 281 unique ones from reference screening and expert consultation); Reasons to exclude correspond to Fig 1 in the paper**

| Index | Publication Year | Author | Title | If included | Reason for exclusion | URL for grey included paper |
|---|---|---|---|---|---|---|
| 25 | 2017 | Ardeshir Tanha, Mandana; Brennan, Paul V.; Ash, Matthew; Kohler, Anselm; McElwaine, Jim | Overlapped Phased Array Antenna for Avalanche Radar | No | Excluded at title/ abstract screening | |
| 26 | 1987 | Armstrong | When things go wrong. | No | Irrelevant title/ abstract (reports from other methods) | |
| 27 | 1987 | Armstrong | Avalanche Accidents in The United States 1950 | No | Irrelevant title/ abstract (reports from other methods) | |
| 28 | 1987 | Armstrong RL, Armstrong BR | Snow and avalanche climates of the western United States: a comparison of maritime, intermountain and continental conditions. | No | Irrelevant title/ abstract (reports from other methods) | |
| 29 | 2015 | Arnold, P.; Dorren, L. | The importance of rockfall and landslide risks on swiss national roads | No | Excluded at title/ abstract screening | |
| 30 | 2007 | Ashour A, Cameron P, Bernard S, et al. | Could bystander first-aid prevent trauma deaths at the scene of injury? | No | Irrelevant title/ abstract (reports from other methods) | |
| 31 | 2000 | Atkins D | Human factors in avalanche accidents. | No | Irrelevant title/ abstract (reports from other methods) | |
| 32 | 2006 | Atkins D. | A history of avalanche accidents, 1895–2006. | No | Irrelevant title/ abstract (reports from other methods) | |
| 33 | 2010 | Atkins, Dale | Ten Years of Avalanche Deaths in the United States, 1999/00 to 2008/09 | No | Excluded at title/ abstract screening | |
| 34 | 2010 | Atkins, Dale | Avalanche Rescue: The United States Experience, 1999/00 to 2008/09 | No | Excluded at title/ abstract screening | |
| 35 | 2012 | Atkins, Dale | Skiers, Trees and Avalanches: A Murderous Triad | No | Excluded at title/ abstract screening | |
| 36 | 1956 | Atwater, Montgomery M. | Fighting the White Death | No | Excluded at title/ abstract screening | |
| 37 | 2003 | August, Melissa; Barovick, Harriet; Bland, Elizabeth L.; Crittle, Simon; Winters, Rebecca | MILESTONES | No | Excluded at title/ abstract screening | |
| 38 | Registry | avalanche.org | avalanche accidents across the United States (2023-2024) | No | Data already covered | |
| 39 | Registry | avalanche.org | avalanche accidents across the United States (2022-2023) | No | Data already covered | |

| **Appendix. Data screening record.** | | | | | | |
|---|---|---|---|---|---|---|
| **This table records inclusion and exclusion decisions (with reasons for exclusion) for 1031 studies/registries (750 unique ones from database search and 281 unique ones from reference screening and expert consultation); Reasons to exclude correspond to Fig 1 in the paper** | | | | | | |
| **Index** | **Publication Year** | **Author** | **Title** | **If included** | **Reason for exclusion** | **URL for grey included paper** |
| **40** | **Registry** | avalanche.org | avalanche accidents across the United States (2021-2022) | No | Data already covered | |
| **41** | **Registry** | avalanche.org | avalanche accidents across the United States (2020-2021) | No | Data already covered | |
| **42** | **Registry** | avalanche.org | avalanche accidents across the United States (2019-2020) | No | Data already covered | |
| **43** | **Registry** | avalanche.org | avalanche accidents across the United States (2018-2019) | No | Data already covered | |
| **44** | **2012** | Avellanas, M. L.; Ricart, A.; Botella, J.; Mengelle, F.; Soteras, I.; Veres, T.; Vidal, M. | Management of severe accidental hypothermia | No | Excluded at title/ abstract screening | |
| **45** | **2017** | Aydin, A.; Eker, R. | GIS-based snow avalanche hazard mapping: Bayburt-asagi dere catchment case | No | Excluded at title/ abstract screening | |
| **46** | **2014** | Ayoung-Chee P, McIntyre L, Ebel BE, et al | Long-term outcomes of ground-level falls in the elderly | No | Irrelevant title/ abstract (reports from other methods) | |
| **47** | **2007** | Ayuso, N.; Cuchí, J. A.; Lera, F.; Villarroel, J. L. | Avalanche beacon magnetic field calculations for rescue techniques improvement | No | Excluded at title/ abstract screening | |
| **48** | **2015** | Ayuso, N.; Cuchí, J. A.; Lera, F.; Villarroel, J. L. | A deep insight into avalanche transceivers for optimizing rescue | No | Excluded at title/ abstract screening | |
| **49** | **2009** | BÃ¼hler, Yves; HÃ¼ni, Andreas; Christen, Marc; Meister, Roland; Kellenberger, Tobias | Automated Detection and Mapping of Rough Snow Surfaces Including Avalanche Deposits Using Airborne Optical Remote Sensing | No | Excluded at title/ abstract screening | |
| **50** | **2005** | Badcock D, Kelly AM, Kerr D, Reade T | The quality of medical record review studies in the international emergency medicine literature. | No | Irrelevant title/ abstract (reports from other methods) | |
| **51** | **2016** | Badoux, A.; Andres, N.; Techel, F.; Hegg, C. | Natural hazard fatalities in Switzerland from 1946 to 2015 | No | No relevant statistics obtained | |
| **52** | **2020** | Bahun, M. V.; Zorn, M. | Avalanches in the municipality of TrŽiČ | No | Excluded at title/ abstract screening | |
| **53** | **2020** | Bahun, Manca Volk; Zorn, Matija | Snežni plazovi v Občini Tržič | No | Excluded at title/ abstract screening | |

**Appendix. Data screening record.**

**This table records inclusion and exclusion decisions (with reasons for exclusion) for 1031 studies/registries (750 unique ones from database search and 281 unique ones from reference screening and expert consultation); Reasons to exclude correspond to Fig 1 in the paper**

| Index | Publication Year | Author | Title | If included | Reason for exclusion | URL for grey included paper |
|---|---|---|---|---|---|---|
| 54 | 2009 | Bair, E. H.; Dozier, J.; Davis, R. E.; Kaempfer, T. U.; Colee, M. T.; Mielke, R.; Blackford, J. R. | Observations of two seasons of sintering in a mountain snowpack | No | Excluded at title/ abstract screening | |
| 55 | 2011 | Baird, Jim | Dome, Sweet Dome | No | Excluded at title/ abstract screening | |
| 56 | 1974 | Baker S.P., O'Neill B., Haddon W., Jr., and Long W.B. | The injury severity score: a method for describing patients with multiple injuries and evaluating emergency care | No | Irrelevant title/ abstract (reports from other methods) | |
| 57 | 2016 | Baker, J.; McGee, T. K. | Backcountry Snowmobilers' Avalanche-Related Information-Seeking and Preparedness Behaviors | No | Excluded at title/ abstract screening | |
| 58 | 2015 | Bakke HK, Steinvik T, Eidissen S-I, et al. | Bystander first aid in trauma - prevalence and quality: a prospective observational study | No | Irrelevant title/ abstract (reports from other methods) | |
| 59 | 2010 | Baldursdotir S, Sigvaldason K, Karason S, Valsson F, Sigurdsson GH. | Induced hypothermia in comatose survivors of asphyxia: a case series of 14 consecutive cases. | No | Irrelevant title/ abstract (reports from other methods) | |
| 60 | 2006 | Baldwin, Sam | JAPAN'S Winter Weather EXPOSED | No | Excluded at title/ abstract screening | |
| 61 | 2004 | Ballard, Henry; Atkins, Dale; Ballard, Lin | Probing for Avalanche Victims | No | Excluded at title/ abstract screening | |
| 62 | 2015 | Ballesteros-Canovas, J. A.; Czajka, B.; Janecka, K.; Lempa, M.; Kaczka, R. J.; Stoffel, M. | Flash floods in the Tatra Mountain streams: Frequency and triggers | No | Excluded at title/ abstract screening | |
| 63 | 2004 | Barbolini, M.; Cappabianca, F.; Sailer, R. | Empirical estimate of vulnerability relations for use in snow avalanche risk assessment | No | Irrelevant title/ abstract (reports from other methods) | |
| 64 | 2012 | Bass, S. A. M. | COME ALONG | No | Excluded at title/ abstract screening | |
| 65 | 2008 | Bastone, Kelly | A KILLER SEASON | No | Excluded at title/ abstract screening | |
| 66 | 2020 | Bates, Josiah | Avalanche at Lake Tahoe Ski Resort Leaves 1 Person Dead, Another Seriously Injured | No | Excluded at title/ abstract screening | |
| 67 | 2003 | Batin, Christopher | How To SURVIVE At 30 Below | No | Excluded at title/ abstract screening | |
| 68 | 2012 | Baugher, Paul | Proceedings, 2012 International Snow Science Workshop, Anchorage, Alaska | No | Irrelevant title/ abstract (reports from other methods) | |

**Appendix. Data screening record.**

**This table records inclusion and exclusion decisions (with reasons for exclusion) for 1031 studies/registries (750 unique ones from database search and 281 unique ones from reference screening and expert consultation); Reasons to exclude correspond to Fig 1 in the paper**

| Index | Publication Year | Author | Title | If included | Reason for exclusion | URL for grey included paper |
|---|---|---|---|---|---|---|
| 69 | 2009 | Baumann, Frank W. | Avalanche fatalities | No | No relevant statistics obtained | |
| 70 | 1987 | Baust, J. G.; Lee Jr, R. E. | Multiple stress tolerance in an antarctic terrestrial arthropod: Belgica antarctica | No | Excluded at title/ abstract screening | |
| 71 | 1997 | Beard, Jonathan | Balloons lift skiers out of danger | No | Excluded at title/ abstract screening | |
| 72 | 2012 | Bebi, P.; Teich, M.; Hagedorn, F.; Zurbriggen, N.; Brunner, S. H.; Grêt-Regamey, A. | Changes in forest cover and ecosystem services in Davos under climate change | No | Excluded at title/ abstract screening | |
| 73 | 2007 | Becker, J. S.; Johnston, D. M.; Paton, D.; Hancox, G. T.; Davies, T. R.; McSaveney, M. J.; Manville, V. R. | Response to landslide dam failure emergencies: Issues resulting from the October 1999 Mount Adams landslide and dam-break flood in the Poerua River, Westland, New Zealand | No | Excluded at title/ abstract screening | |
| 74 | 2003 | Beech, Mark; Kennedy, Kostya | Death of a Free Rider | No | Excluded at title/ abstract screening | |
| 75 | 2014 | Beekman, Kimberly | CAROLINE GLEICH | No | Excluded at title/ abstract screening | |
| 76 | 2004 | Bell, R.; Glade, T. | Multi-hazard analysis in natural risk assessments | No | Excluded at title/ abstract screening | |
| 77 | 2010 | Berg, Emmett | CINEMA VERTIGO | No | Excluded at title/ abstract screening | |
| 78 | 1982 | Berghold, F. | Avalanche disasters | No | Excluded at title/ abstract screening | |
| 79 | 1991 | Berghold, F.; Seidl, A. M. | Snowboard accidents in the Alps. Risks, accident analysis and injury profile | No | Excluded at title/ abstract screening | |
| 80 | 1982 | Berghold, F.. et al.: | Medical Symposium on Avalanche-Caused Accidents. | No | Irrelevant title/ abstract (reports from other methods) | |
| 81 | 2003 | Bergman, Brian | A SEASON OF GRIEF | No | Excluded at title/ abstract screening | |
| 82 | 2019 | Berlin, Claudia; Techel, Frank; Moor, Beat Kaspar; Zwahlen, Marcel; Hasler, Rebecca Maria; Swiss National Cohort study, group | Snow avalanche deaths in Switzerland from 1995 to 2014- Results of a nation-wide linkage study | No | No relevant statistics obtained | |
| 83 | 2019 | Biancalana, R. C.; Vincenti, S. A. F.; da Silva, R. H. A.; Pires-de-Souza, F. C. P. | Analysis of the surface roughness and microhardness of dental restorative materials exposed to heat sources and cold temperatures for human identification purposes | No | Excluded at title/ abstract screening | |

| Appendix. Data screening record. | | | | | | |
|---|---|---|---|---|---|---|
| This table records inclusion and exclusion decisions (with reasons for exclusion) for 1031 studies/registries (750 unique ones from database search and 281 unique ones from reference screening and expert consultation); Reasons to exclude correspond to Fig 1 in the paper | | | | | | |
| **Index** | **Publication Year** | **Author** | **Title** | **If included** | **Reason for exclusion** | **URL for grey included paper** |
| 84 | 2017 | Bianchi G, Brügger O, Niemann S | Skiing and snowboarding in Switzerland: trends in injury and fatality rates over time. | No | Excluded at title/abstract screening | |
| 85 | 2012 | Binger, Jonathan; Babineau-Z, Chelan | Partner Rescue Skills: An Organizational and Individual Assessment Over the 2010-11 and 2011-12 Winter Seasons at Big Sky Resort | No | Excluded at title/abstract screening | |
| 86 | 2022 | Bjertnæs, Lars J.; Næsheim, Torvind O.; Reierth, Eirik; Suborov, Evgeny V.; Kirov, Mikhail Y.; Lebedinskii, Konstantin M.; Tveita, Torkjel | Physiological Changes in Subjects Exposed to Accidental Hypothermia: An Update | No | Excluded at title/abstract screening | |
| 87 | 1977 | Bjornsson, H. | Snow avalanche studies in Iceland | No | Excluded at title/abstract screening | |
| 88 | 2018 | Blancher, Marc; Albasini, François; Elsensohn, Fidel; Zafren, Ken; Hölzl, Natalie; McLaughlin, Kyle; Wheeler, Albert R.; Roy, Steven; Brugger, Hermann; Greene, Mike; Paal, Peter | Management of Multi-Casualty Incidents in Mountain Rescue: Evidence-Based Guidelines of the International Commission for Mountain Emergency Medicine (ICAR MEDCOM) | No | Excluded at title/abstract screening | |
| 89 | 2017 | Blancher, Marc; Bauvent, Yann; Baré, Stéphane; Wuyts, Ben; Fillet, Yann; Brun, Julien; Albasini, François; Bouzat, Pierre | Multiple casualty incident in the mountain: Experience from the Valfrejus avalanche | Yes, only for systematic review | NA, since included | |
| 90 | 2013 | Blanco, R.; Peman, J.; Rodriguez, F.; Aunos, A. | Comparative state of the status of pinus uncinata ram. potentially protective against snow of a zone andorra and catalonia (NE of Spain) | No | Excluded at title/abstract screening | |
| 91 | 2017 | Blattenberger, G.; Fowles, R. | Treed Avalanche Forecasting: Mitigating Avalanche Danger Utilizing Bayesian Additive Regression Trees | No | Excluded at title/abstract screening | |
| 92 | 2021 | Blondin, S. | Staying despite disaster risks: Place attachment, voluntary immobility and adaptation in Tajikistan's Pamir Mountains | No | Excluded at title/abstract screening | |

**Appendix. Data screening record.**

**This table records inclusion and exclusion decisions (with reasons for exclusion) for 1031 studies/registries (750 unique ones from database search and 281 unique ones from reference screening and expert consultation); Reasons to exclude correspond to Fig 1 in the paper**

| Index | Publication Year | Author | Title | If included | Reason for exclusion | URL for grey included paper |
|---|---|---|---|---|---|---|
| 93 | 2010 | Bogle, Lee B.; Boyd, Jeff J.; McLaughlin, Kyle A. | Triaging multiple victims in an avalanche setting: the Avalanche Survival Optimizing Rescue Triage algorithmic approach | No | No relevant statistics obtained | |
| 94 | 2011 | Bohm K, Vaillancourt C, Charette ML, et al | In patients with out-of-hospital cardiac arrest, does the provision of dispatch cardiopulmonary resuscitation instructions as opposed to NO instructions improve outcome: a systematic review of the literature. | No | Irrelevant title/ abstract (reports from other methods) | |
| 95 | 2019 | Bonini, N.; Pighin, S.; Rettore, E.; Savadori, L.; Schena, F.; Tonini, S.; Tosi, P. | Overconfident people are more exposed to "black swan" events: a case study of avalanche risk | No | Irrelevant title/ abstract (reports from other methods) | |
| 96 | 2009 | Boucher, Dominic; HÃ©tu, Bernard | La â€˜mort blancheâ€™ au QuÃ©bec (Canada) depuis 1825 : de la prise de conscience du pr oblÃ¨me Ã la gestion du risque | No | Excluded at title/ abstract screening | |
| 97 | 2014 | Boucher, Dominic; LeBlanc, Julie; Janssen, Laurent | Centre D'avalanche De La Haute Gaspasie: 15 Years of Avalanche Safety in Quebec | No | Excluded at title/ abstract screening | |
| 98 | 2014 | Boué Y, Payen J-F, Torres J-P, Blancher M, Bouzat P | Full neurologic recovery after prolonged avalanche burial and cardiac arrest | No | Irrelevant title/ abstract (reports from other methods) | |
| 99 | 2014 | Boue Y, Payer JF, Brun J, et al | Survival after avalanche-induced cardiac arrest. | No | Irrelevant title/ abstract (reports from other methods) | |
| 100 | 2014 | Boué, Y.; Brun, J.; Thomas, S.; Levrat, A.; Blancher, M.; Debaty, G.; Payen, J.-F.; Bouzat, P. | Analyse de la survie des patients avalanchés en arrêt cardiaque dans les Alpes du Nord | No | Irrelevant title/ abstract (reports from other methods) | |
| 101 | 2019 | Bourne, J. R.; Leang, K. K. | Bayesian estimation of snow-avalanche victim pose: A method to assist human and/ or robot first responders to quickly locate a buried victim | No | Excluded at title/ abstract screening | |
| 102 | 1994 | Bowers, Vivien | Avalanche! | No | Excluded at title/ abstract screening | |
| 103 | 2010 | Boyd, J.; Brugger, H.; Shuster, M. | Prognostic factors in avalanche resuscitation: A systematic review | No | Excluded at title/ abstract screening | |

**Appendix. Data screening record.**

**This table records inclusion and exclusion decisions (with reasons for exclusion) for 1031 studies/registries (750 unique ones from database search and 281 unique ones from reference screening and expert consultation); Reasons to exclude correspond to Fig 1 in the paper**

| Index | Publication Year | Author | Title | If included | Reason for exclusion | URL for grey included paper |
|---|---|---|---|---|---|---|
| 104 | 2009 | Boyd, Jeff; Haegeli, Pascal; Abu-Laban, Rryad B.; Shuster, Michael; John, C. Butt | Patterns of death among avalanche fatalities: a 21-year review | Yes, for meta-analysis and systematic review | NA, since included | |
| 105 | 2021 | Branch, John | Virus Rules May Factor Into Avalanche Deaths | No | Excluded at title/ abstract screening | |
| 106 | 2004 | Brang, P.; Schönenberger, W.; Fischer, A. | Reforestation in Central Europe: Lessons from multi-disciplinary field experiments | No | Excluded at title/ abstract screening | |
| 107 | 2020 | Bratosin, I. A.; Pavaloiu, I. B.; Vasilateanu, A.; Goga, N.; Dragoi, G.; Gavajuc, D. | Virtual reality therapy for pain | No | Excluded at title/ abstract screening | |
| 108 | 1976 | Braun P. Probleme der Ersten Hilfe beim Lawinenunfall. | Tagung Uber Medizinische Aspekte des Lawinenunfalls | No | Irrelevant title/ abstract (reports from other methods) | |
| 109 | 2012 | Breien, Hedda; HÃ¸ydal, Ã˜yvind A. | Influence of Forest on Snow Avalanche Hazard â€“ Norwegian Challenges | No | Excluded at title/ abstract screening | |
| 110 | 2010 | Bright, L. Shay | Group Dynamics and Decision Making: Backcountry Recreationists in Avalanche Terrain | No | Excluded at title/ abstract screening | |
| 111 | 2016 | Brigo, F.; Strapazzon, G.; Otte, W. M.; Igwe, S. C.; Brugger, H. | Web search behavior for snow avalanches: an Italian study | No | Excluded at title/ abstract screening | |
| 112 | 1998 | Brogdon BG. | The scope of forensic radiology. | No | Irrelevant title/ abstract (reports from other methods) | |
| 113 | 1996 | Brooke, James | Abundance of snowfall, and daring, proves deadly | No | Excluded at title/ abstract screening | |
| 114 | 2014 | Brown, Chip | On what would be the darkest day in the history of the world's highest mountain | No | Excluded at title/ abstract screening | |
| 115 | 2009 | Brugger H | Should strategies for care of avalanche victims change? | No | Irrelevant title/ abstract (reports from other methods) | |
| 116 | 2002 | Brugger H, Durrer B | International Commission for Mountain Emergency Medicine. On-site treatment of avalanche victims ICAR-MEDCOM-recommendation. | No | Irrelevant title/ abstract (reports from other methods) | |
| 117 | 1996 | Brugger H, Durrer B, Adler-Kastner L | On-site triage of avalanche victims with asystole by the emergency doctor | No | Irrelevant title/ abstract (reports from other methods) | |

**Appendix. Data screening record.**

**This table records inclusion and exclusion decisions (with reasons for exclusion) for 1031 studies/registries (750 unique ones from database search and 281 unique ones from reference screening and expert consultation); Reasons to exclude correspond to Fig 1 in the paper**

| Index | Publication Year | Author | Title | If included | Reason for exclusion | URL for grey included paper |
|---|---|---|---|---|---|---|
| 118 | 1997 | Brugger H, Falk M, Adler-Kastner L. | Der Lawinennotfall Neue Aspekte zur Pathophysiologie und Therapie von Lawinenvershüttete n. | No | Irrelevant title/ abstract (reports from other methods) | |
| 119 | 2002 | Brugger H, Flora G, Falk M | Self-rescue-techniques and post-traumatic stress disorders in avalanche accidents | No | Irrelevant title/ abstract (reports from other methods) | |
| 120 | 1997 | Brugger H., Falk M., and Adler-Kastner L. (1997). | New aspects of the pathophysiology and therapy of buried avalanche victims | No | Irrelevant title/ abstract (reports from other methods) | |
| 121 | 2001 | Brugger, H.; Durrer, B.; Adler-Kastner, L.; Falk, M.; Tschirky, F. | Field management of avalanche victims | No | Excluded at title/ abstract screening | |
| 122 | 1992 | Brugger, H.; Falk, M. | New perspectives of avalanche disasters. Phase classification using pathophysiologic considerations | No | Excluded at title/ abstract screening | |
| 123 | 1997 | Brugger, H.; Falk, M.; Adler-Kastner, L. | Avalanche emergencies. New perspectives on the pathophysiology and management of persons buried in an avalanche | No | Excluded at title/ abstract screening | |
| 124 | 2008 | Brugger, H.; Paal, P.; Hohlrieder, M.; Sumann, G. | Medical aspects of avalanche accidents | No | Excluded at title/ abstract screening | |
| 125 | 2009 | Brugger, Hermann | Causes of Death From Avalanche | No | Excluded at title/ abstract screening | |
| 126 | 2013 | Brugger, Hermann; Durrer, Bruno; Elsensohn, Fidel; Paal, Peter; Strapazzon, Giacomo; Winterberger, Eveline; Zafren, Ken; Boyd, Jeff | Resuscitation of avalanche victims: Evidence-based guidelines of the international commission for mountain emergency medicine (ICAR MEDCOM): Intended for physicians and other advanced life support personnel | No | Excluded at title/ abstract screening | |
| 127 | 2007 | Brugger, Hermann; Etter, Hans Jürg; Zweifel, Benjamin; Mair, Peter; Hohlrieder, Matthias; Ellerton, John; Elsensohn, Fidel; Boyd, Jeff; Sumann, Günther; Falk, Markus | The impact of avalanche rescue devices on survival | No | No relevant statistics obtained | |

| Appendix. Data screening record. | | | | | | |
|---|---|---|---|---|---|---|
| **This table records inclusion and exclusion decisions (with reasons for exclusion) for 1031 studies/registries (750 unique ones from database search and 281 unique ones from reference screening and expert consultation); Reasons to exclude correspond to Fig 1 in the paper** | | | | | | |
| **Index** | **Publication Year** | **Author** | **Title** | **If included** | **Reason for exclusion** | **URL for grey included paper** |
| **128** | **2003** | Brugger, Hermann; Sumann, Günther; Meister, Roland; Adler-Kastner, Liselotte; Mair, Peter; Gunga, Hanns Christian; Schobersberger, Wolfgang; Falk, Markus; Sumann, Günther | Hypoxia and hypercapnia during respiration into an artificial air pocket in snow: implications for avalanche survival | No | Excluded at title/ abstract screening | |
| **129** | **2013** | Bruijns SR, Guly HR, Bouamra O, et al. | The value of traditional vital signs, shock index, and age-based markers in predicting trauma mortality. | No | Excluded at title/ abstract screening | |
| **130** | **2004** | Bründl, M.; Etter, H. J.; Steiniger, M.; Klingler, Ch; Rhyner, J.; Ammann, W. J. | IFKIS - A basis for managing avalanche risk in settlements and on roads in Switzerland | No | Excluded at title/ abstract screening | |
| **131** | **1989** | Bryant, C. L.; Butler, D. R.; Vitek, J. D. | A statistical analysis of tree-ring dating in conjunction with snow avalanches: Comparison of on-path versus off-path responses | No | Excluded at title/ abstract screening | |
| **132** | **2003** | Buchanan, Rob | Quest One | No | Excluded at title/ abstract screening | |
| **133** | **2001** | Buchanan, Rob | Where East Meets West | No | Excluded at title/ abstract screening | |
| **134** | **2006** | Burrows, Robert A.; McClung, David M. | Snow Cornice Development and Failure Monitoring | No | Excluded at title/ abstract screening | |
| **135** | **2012** | Burton, Charles Dustin | Lateral Snow Transport, Fire and Changing Treelines in Mount San Gorgonio, California, U.S.A | No | Excluded at title/ abstract screening | |
| **136** | **1995** | Burtscher M, Nachbauer W, Likar R” | The risk of death to trekkers and hikers in the mountains | No | No relevant data (full text examined, papers through other methods) | |
| **137** | **2010** | Burtscher M, Ponchia A | The risk of cardiovascular events during leisure time activities at altitude | No | Irrelevant title/ abstract (reports from other methods) | |
| **138** | **2007** | Burtscher M. | Risk of cardiovascular events during mountain activities. | No | Irrelevant title/ abstract (reports from other methods) | |
| **139** | **1999** | Burtscher, M.; Nachbauer, W. | Effects of training on the risk of avalanche fatality | No | Excluded at title/ abstract screening | |
| **140** | **2009** | Butler, D. R.; Malanson, G. P.; Resler, L. M.; Walsh, S. J.; Wilkerson, F. D.; Schmid, G. L.; Sawyer, C. F. | Geomorphic Patterns and Processes at Alpine Treeline | No | Excluded at title/ abstract screening | |

| Appendix. Data screening record. | | | | | | |
|---|---|---|---|---|---|---|
| This table records inclusion and exclusion decisions (with reasons for exclusion) for 1031 studies/registries (750 unique ones from database search and 281 unique ones from reference screening and expert consultation); Reasons to exclude correspond to Fig 1 in the paper | | | | | | |
| **Index** | **Publication Year** | **Author** | **Title** | **If included** | **Reason for exclusion** | **URL for grey included paper** |
| **141** | **2014** | CAA | Observation guidelines and recording standards for weather, snowpack and avalanches | No | Irrelevant title/ abstract (reports from other methods) | |
| **142** | **2021** | Caballero Merlo, Javier Numan | La ciudadanía en Paraguay no existe: la pandemia como despliegue del biopoder | No | Excluded at title/ abstract screening | |
| **143** | **1999** | Cadman, R. | How to stay alive in deep powder snow: avoiding tree-well accidents | No | Excluded at title/ abstract screening | |
| **144** | **1999** | Cadman, R. | Eight nonavalanche snow-immersion deaths - A 6-year series from British Columbia ski areas | No | Excluded at title/ abstract screening | |
| **145** | **2022** | Cainelli, Elisa; Vedovelli, Luca; Gregori, Dario; Suppiej, Agnese; Padalino, Massimo; Cogo, Paola; Bisiacchi, Patrizia | Embrace the Complexity: Agnostic Evaluation of Children's Neuropsychological Performances Reveals Hidden Neurodevelopment Patterns | No | Excluded at title/ abstract screening | |
| **146** | **2015** | Callaghan, Anna | Bombs over Bald Mountain | No | Excluded at title/ abstract screening | |
| **147** | **2007** | Campbell C, Bakermans L, Jamieson B, et al | Current and future snow avalanche threats and mitigation measures in Canada. | | Irrelevant title/ abstract (reports from other methods) | |
| **148** | **2010** | Campbell, Cam; MacDonald, Matt | A Recipe for Widespread Persistent Deep Slab Avalanche Characteristics in Western Canada | No | Excluded at title/ abstract screening | |
| **149** | **2010** | Campbell, Cam; Marshall, Peter | Mapping Exposure to Avalanche Terrain | No | Excluded at title/ abstract screening | |
| **150** | **2007** | Canadian Avalanche Association. | Reporting avalanche incidents. In: Observation guidelines and recording standards for weather, snowpack and avalanches | No | Data already covered | |
| **151** | **2008** | Canadian Avalanche Centre | Youth programs. Revelstoke (BC): Canadian Avalanche Centre | No | Data already covered | |
| **152** | **2022** | Cao, Y.; Song, M. S. | Crowding perception predicts subway passengers' behavioral decision making during COVID-19 via risk awareness and safety perception | No | Excluded at title/ abstract screening | |

**Appendix. Data screening record.**

**This table records inclusion and exclusion decisions (with reasons for exclusion) for 1031 studies/registries (750 unique ones from database search and 281 unique ones from reference screening and expert consultation); Reasons to exclude correspond to Fig 1 in the paper**

| Index | Publication Year | Author | Title | If included | Reason for exclusion | URL for grey included paper |
|---|---|---|---|---|---|---|
| 153 | 2019 | Carceller, Anna; Javierre, Casimiro; Ríos, Martín; Viscor, Ginés | Amputation Risk Factors in Severely Frostbitten Patients | No | Excluded at title/ abstract screening | |
| 154 | 2002 | Cardwell, T. | What to do in an avalanche | No | Excluded at title/ abstract screening | |
| 155 | 2005 | Carey, Mark | Living and dying with glaciers: people's historical vulnerability to avalanches and outburst floods in Peru | No | Excluded at title/ abstract screening | |
| 156 | 1993 | Carlson, Lee; Lerman, Josh | A beautiful balloon | No | Excluded at title/ abstract screening | |
| 157 | 2006 | Carrara, P.; Freppaz, M.; Maggioni, M.; Piccini, P.; Zanini, E. | Fuzzy Modeling of the Snowpack Evolution | No | Excluded at title/ abstract screening | |
| 158 | 1999 | Carton, Barbara | Spate of Avalanches Sparks Demand for Survival Gear | No | Excluded at title/ abstract screening | |
| 159 | 2008 | Casteller, A.; Christen, M.; Villalba, R.; Martínez, H.; Stöckli, V.; Leiva, J. C.; Bartelt, P. | Validating numerical simulations of snow avalanches using dendrochronology: The Cerro Ventana event in Northern Patagonia, Argentina | No | Excluded at title/ abstract screening | |
| 160 | 1978 | Central Bureau of Statistics of Norway | Environmental statistics 1978. Natural Resoursces and Pollution | No | Irrelevant title/ abstract (reports from other methods) | |
| 161 | | Cesar, G. Soriano | Alps see climb in avalanche fatalities | No | Excluded at title/ abstract screening | |
| 162 | 1990 | Champion HR, Copes WS, Sacco WJ, et al | Trauma Outcome Study: establishing national norms for trauma care | No | Irrelevant title/ abstract (reports from other methods) | |
| 163 | 1990 | Champion HR, Copes WS, Sacco WJ, et al. | The major trauma outcome study: establishing national norms for trauma care. | No | Irrelevant title/ abstract (reports from other methods) | |
| 164 | 2023 | Champion, Nikki; Paradis, Andy; Kobernik, Brett | EVALUATING UTAH AVALANCHE CENTER FORECASTS: COMPARING REPORTED AVALANCHE ACTIVITY WITH THE FORECASTED AVALANCHE PROBLEMS. | No | Excluded at title/ abstract screening | |
| 165 | 2016 | Chan, Sewell; Sandomir, Richard | SPORTS BRIEFING | No | Excluded at title/ abstract screening | |
| 166 | 2011 | Chang, E. I.; Carlson, G. A.; Vose, J. G.; Huang, E. J.; Yang, G. P. | Comparative Healing of Rat Fascia Following Incision with Three Surgical Instruments | No | Excluded at title/ abstract screening | |

| Appendix. Data screening record. | | | | | | |
|---|---|---|---|---|---|---|
| **This table records inclusion and exclusion decisions (with reasons for exclusion) for 1031 studies/registries (750 unique ones from database search and 281 unique ones from reference screening and expert consultation); Reasons to exclude correspond to Fig 1 in the paper** | | | | | | |
| **Index** | **Publication Year** | **Author** | **Title** | **If included** | **Reason for exclusion** | **URL for grey included paper** |
| **167** | **2007** | Chardon, G.; Bonneterre, V.; Bernardet, C.; De Gaudemaris, R. | Interest of airbag backpack as right assistance for snow avalanches | No | Excluded at title/ abstract screening | |
| **168** | **2021** | Charles, Krista | Russian mystery solved by unusual avalanche | No | Excluded at title/ abstract screening | |
| **169** | **2011** | Chen, F.; Deng, J.; Wei, J.; Tai, J. | Behaviour of slope protecting and retaining structures during Wenchuan earthquake on May 12 2008 | No | Excluded at title/ abstract screening | |
| **170** | **2011** | Chen, X. L.; Li, J. H.; Xin, X.; Zhang, Z. E.; Xin, P. P.; Lu, X. X. | Cryopreservation of in vitro-grown apical meristems of Lilium by droplet-vitrification | No | Excluded at title/ abstract screening | |
| **171** | **2015** | Chenthil, T. R.; Suman, M.; Srinivasan, M. | Design and implementation of detection of buried human and animal bodies under debris victims and localization them using ground penetrating radars (GPR) using CW signals | No | Excluded at title/ abstract screening | |
| **172** | **2017** | Chevalier M | Locate an avalanche victim using the DVA. | No | Excluded at title/ abstract screening | |
| **173** | **2015** | Chigira, M.; Hiraishi, N.; Ching-Ying, T.; Matsushi, Y. | Catastrophic Landslides and Their Precursory Deep-Seated Gravitational Slope Deformation Induced by the River Rejuvenation in the Kii Mountains, Central Japan | No | Excluded at title/ abstract screening | |
| **174** | **2003** | Child, Greg | BURIED IN THE PAST | No | Excluded at title/ abstract screening | |
| **175** | **2012** | Chin, Jimmy | Caught in an Avalanche | No | Excluded at title/ abstract screening | |
| **176** | **1999** | Christensen, E. D.; Lacsina, E. Q. | Mountaineering fatalities on Mount Rainier, Washington, 1977-1997 - Autopsy and investigative findings | Yes, for meta-analysis and systematic review | NA, since included | |
| **177** | **2009** | Christina, E. R. B. | Avalanche Deaths and Hazards Rattling Ski Country in the West | No | Excluded at title/ abstract screening | |
| **178** | **2009** | Chrustek, P.; Wezyk, P. | Using high resolution LiDAR data to estimate potential avalanche release areas on the example of Polish mountain regions | No | Excluded at title/ abstract screening | |

| **Appendix. Data screening record.** | | | | | | |
|---|---|---|---|---|---|---|
| **This table records inclusion and exclusion decisions (with reasons for exclusion) for 1031 studies/registries (750 unique ones from database search and 281 unique ones from reference screening and expert consultation); Reasons to exclude correspond to Fig 1 in the paper** | | | | | | |
| **Index** | **Publication Year** | **Author** | **Title** | **If included** | **Reason for exclusion** | **URL for grey included paper** |
| **179** | **2013** | Cierco, FranÃ§ois-Xavier; Debouck, Frank | Human Factors in Decision Making in Avalanche Terrain | No | Excluded at title/abstract screening | |
| **180** | **2005** | Clarke Jr, John | Search Turns To Recovery In Avalanche Near Ski Area | No | Excluded at title/abstract screening | |
| **181** | **1977** | Coe JI | Postmortem chemistry of blood, cerebrospinal fluid, and vitreous humor | No | Irrelevant title/abstract (reports from other methods) | |
| **182** | **1984** | Coe JI | Hypothermia: autopsy findings and vitreous glucose | No | Irrelevant title/abstract (reports from other methods) | |
| **183** | **1967** | Cohen J. | Weighted kappa — nominal scale agreement with provision for degrees of disagreement. | No | Excluded at title/abstract screening | |
| **184** | **1992** | Collopy B.T., Tulloh B.R., Rennie G.C., Fink R.L., Rush J.H., and Trinca G.W. | Correlation between injury severity scores and subjective ratings of injury severity: a basis for trauma audit. | No | Irrelevant title/abstract (reports from other methods) | |
| **185** | **2014** | Conlan, Michael; Jamieson, Bruce | An Expert Opinion Survey for the Development of a Decision Support Tool for Persistent Deep Slab Avalanche Forecasting | No | Excluded at title/abstract screening | |
| **186** | **2018** | Conner, Mary M.; Stephenson, Thomas R.; German, David W.; Monteith, Kevin L.; Few, Alexandra P.; Bair, Edward H. | Survival analysis: Informing recovery of Sierra Nevada bighorn sheep | No | No relevant statistics obtained | |
| **187** | **1980** | Conover WJ. | Practical nonparametric statistics | No | Irrelevant title/abstract (reports from other methods) | |
| **188** | **1999** | Cook, Kevin; Mravic, Mark | Death in the Himalayas | No | No relevant statistics obtained | |
| **189** | **1972** | Cooley DA, Reul GJ, Wukasch DC | Ischemic contracture of the heart: "stone heart" | No | Irrelevant title/abstract (reports from other methods) | |
| **190** | **2013** | Copil, Paul Bonhomme and | Training (Formation) of the Followers and the Professionals | No | Excluded at title/abstract screening | |
| **191** | **2011** | Cui, P.; Chen, X. Q.; Zhu, Y. Y.; Su, F. H.; Wei, F. Q.; Han, Y. S.; Liu, H. J.; Zhuang, J. Q. | The Wenchuan Earthquake (May 12, 2008), Sichuan Province, China, and resulting geohazards | No | Excluded at title/abstract screening | |

| Appendix. Data screening record. | | | | | | |
|---|---|---|---|---|---|---|
| **This table records inclusion and exclusion decisions (with reasons for exclusion) for 1031 studies/registries (750 unique ones from database search and 281 unique ones from reference screening and expert consultation); Reasons to exclude correspond to Fig 1 in the paper** | | | | | | |
| **Index** | **Publication Year** | **Author** | **Title** | **If included** | **Reason for exclusion** | **URL for grey included paper** |
| **192** | **2003** | Dale, Virginia H.; Adams, Wendy M. | Plant reestablishment 15 years after the debris avalanche at Mount St. Helens, Washington | No | Excluded at title/ abstract screening | |
| **193** | **2004** | Daley, Jason | I WILL SURVIVE: OUT OF THE VOID | No | Excluded at title/ abstract screening | |
| **194** | **2014** | Dallo, F.; Gabrieli, J.; Diverio, S.; Iaboni, M.; Santoro, M. M.; Di Mari, W.; Barbante, C. | Which human odor cues do avalanche search dogs perceive? | No | Excluded at title/ abstract screening | |
| **195** | **2012** | Dan, Bilefsky | Avalanche Kills at Least 9 Climbers in France's Mont Blanc Mountain Range | No | Excluded at title/ abstract screening | |
| **196** | **2009** | Dana, Sullivan | Surviving an Avalanche | No | Excluded at title/ abstract screening | |
| **197** | **2021** | Daniel, N.; Weinberg, N.; Carus, F.; Church, B.; Zafren, K. | Witnessed Cardiac Arrest in a Hypothermic Avalanche Victim Completely Buried for 2 Hours | No | Study a single cause of death | |
| **198** | **2016** | David, B.; Pivoru, M.; Pivoru, W.; Green, M.; Barker, B.; Weiner, J. F.; Simala, D.; Kokents, T.; Araho, L.; Dop, J. | Living landscapes of the dead: Archaeology of the afterworld among the rumu of Papua New Guinea | No | Excluded at title/ abstract screening | |
| **199** | **2008** | Davidson, Lee | Tragedy in the Adventure Playground: Media Representations of Mountaineering Accidents in New Zealand | No | Excluded at title/ abstract screening | |
| **200** | **2006** | Davies, Charlotte | WHITE DEATH | No | Excluded at title/ abstract screening | |
| **201** | **2021** | Decaulne, A.; Bhiry, N.; Faucher-Roy, J.; Boily, C. P. | The development of Kangiqsualujjuaq and the threat of snow avalanches in a permafrost degradation context, Nunavik, Canada | No | Excluded at title/ abstract screening | |
| **202** | **2019** | Decaulne, Armelle; Bhiry, Najat | Past, present and future slope dynamics under changing climate and society: case studies in Nunavik, northern Québec, Canada | No | Excluded at title/ abstract screening | |
| **203** | **2006** | Dedouit F, Otal P, Costagliola R, Loubes Lacroix F, Telmon N, Rouge D, et al. | Role of modern cross-sectional imaging in thanatology: a pictorial essay. | No | Irrelevant title/ abstract (reports from other methods) | |

**Appendix. Data screening record.**

**This table records inclusion and exclusion decisions (with reasons for exclusion) for 1031 studies/registries (750 unique ones from database search and 281 unique ones from reference screening and expert consultation); Reasons to exclude correspond to Fig 1 in the paper**

| Index | Publication Year | Author | Title | If included | Reason for exclusion | URL for grey included paper |
|---|---|---|---|---|---|---|
| 204 | 2023 | Degawa, Azusa | CHARACTERISTICS AND TRENDS OF AVALANCHE FATALITIES IN JAPAN （1991-2020） | Yes, for meta-analysis and systematic review | NA, since included | |
| 205 | 2006 | Degawa, Azusa; Ikeda, Shinji; Saotome, Yukinori | Case Report: Avalanche Accidents for Recreational Users 2005-6 Winter in Japan | No | Excluded at title/ abstract screening | |
| 206 | 2022 | DeLoughery, E. P.; DeLoughery, T. G. | Review and Analysis of United States Mountaineering Accidents 1947-2018 | No | No relevant statistics obtained | |
| 207 | 2022 | DeLoughery, Emma P.; DeLoughery, Thomas G. | Review and Analysis of Mountaineering Accidents in the United States from 1947-2018 | No | No relevant statistics obtained | |
| 208 | 2010 | Delunel, R.; Hantz, D.; Braucher, R.; Bourlès, D. L.; Schoeneich, P.; Deparis, J. | Surface exposure dating and geophysical prospecting of the Holocene Lauvitel rock slide (French Alps) | No | Irrelevant title/ abstract (reports from other methods) | |
| 209 | 1997 | deMaglia, J. B. | Basic cardiopulmonary resuscitation at the mountains and other isolation situations | No | Irrelevant title/ abstract (reports from other methods) | |
| 210 | 1998 | Demers, Jim | A thundering white death | No | Excluded at title/ abstract screening | |
| 211 | 2008 | Denoth, A. | 40 Years of Snow Physics at the University of Innsbruck, Austria: An Overview of Basic Studies, Development of Instruments and Results | No | Excluded at title/ abstract screening | |
| 212 | 2009 | Derose, R. J.; Long, J. N. | Wildfire and spruce beetle outbreak: Simulation of interacting disturbances in the central rocky mountains | No | Excluded at title/ abstract screening | |
| 213 | 2017 | Dickson TJ, Trathen S, Terwiel FA, et al. | Head injury trends and helmet use in skiers and snowboarders in western Canada, 2008-2009 to 2012–2013: an ecological study. | No | Irrelevant title/ abstract (reports from other methods) | |
| 214 | 2009 | Diggins, Mark | The Challenges for Scottish Avalanche Forecasters Observing a Maritime Snowpack | No | Excluded at title/ abstract screening | |

**Appendix. Data screening record.**

**This table records inclusion and exclusion decisions (with reasons for exclusion) for 1031 studies/registries (750 unique ones from database search and 281 unique ones from reference screening and expert consultation); Reasons to exclude correspond to Fig 1 in the paper**

| Index | Publication Year | Author | Title | If included | Reason for exclusion | URL for grey included paper |
|---|---|---|---|---|---|---|
| 215 | 2006 | Dirnhofer R, Jackowski C, Vock P, Potter K, Thali MJ | VIRTOPSY: Minimally Invasive, Imaging-guided Virtual Autopsy. | No | Irrelevant title/abstract (reports from other methods) | |
| 216 | 2016 | Diverio, S.; Barbato, O.; Cavallina, R.; Guelfi, G.; Iaboni, M.; Zasso, R.; Di Mari, W.; Santoro, M. M.; Knowles, T. G. | A simulated avalanche search and rescue mission induces temporary physiological and behavioural changes in military dogs | No | Excluded at title/abstract screening | |
| 217 | 1999 | Dixon, R. W.; Butler, D. R.; DeChano, L. M.; Henry, J. A. | Avalanche hazard in glacier national park: An EL NiÑo connection? | No | Excluded at title/abstract screening | |
| 218 | 1996 | Dobson J, Burgess J. R | Resuscitation of severe hypothermia by extracorporeal rewarming in a child | No | Irrelevant title/abstract (reports from other methods) | |
| 219 | 2022 | Dorren, L.; Moos, C. | Towards quantitative evidence of Eco-DRR in mountains: A concise review | No | Excluded at title/abstract screening | |
| 220 | 2004 | Dubé, S.; Filion, L.; Hétu, B. | Tree-ring reconstruction of high-magnitude snow avalanches in the northern Gaspé Peninsula, Québec, Canada | No | Excluded at title/abstract screening | |
| 221 | 2013 | Duclos, Alain; VallÃ©e, Thierry | New Technologies: What Roles in the Education and Training? Feedback and Examples | No | Excluded at title/abstract screening | |
| 222 | 1987 | Duffaut, Pierre | Earthquake and Landslide at Mont Ontake (Japan) | No | Excluded at title/abstract screening | |
| 223 | 2006 | Dümbgen L, Freitag-Wolf S, Jonbloed G | Estimating a unimodal distribution from interval-censored data. | No | Irrelevant title/abstract (reports from other methods) | |
| 224 | 1997 | Eckart K | Injured climber flown to safety: partner's body still on Rainier | No | Irrelevant title/abstract (reports from other methods) | |
| 225 | 2013 | Eckert, Nicolas; Lavigne, Aurore; Castebrunet, HÃ©lÃ¨ne; Giraud, GÃ©rald; Naaim, Mohamed | Recent Changes in Avalanche Activity in the French Alps and Their Links With Climatic Drivers: An Overview | No | Excluded at title/abstract screening | |
| 226 | 1994 | Edgerly B | THE ABC's (and D) OF DIGGING: Avalanche Shoveling Distilled to the Basics | No | Irrelevant title/abstract (reports from other methods) | |
| 227 | 2010 | Edgerly B. | Under the radar: using "new school" media to capture unreported avalanche incidents. | No | Irrelevant title/abstract (reports from other methods) | |

| **Appendix. Data screening record.** | | | | | | |
|---|---|---|---|---|---|---|
| **This table records inclusion and exclusion decisions (with reasons for exclusion) for 1031 studies/registries (750 unique ones from database search and 281 unique ones from reference screening and expert consultation); Reasons to exclude correspond to Fig 1 in the paper** | | | | | | |
| **Index** | **Publication Year** | **Author** | **Title** | **If included** | **Reason for exclusion** | **URL for grey included paper** |
| 228 | 2003 | Egan, Timothy | Courting Disaster, in Search of a Snowy Rush. (Cover story) | No | Excluded at title/ abstract screening | |
| 229 | 2020 | Egger A, Niederer M, Tscherny K, et al. | Influence of physical strain at high altitude on the quality of cardiopulmonary resuscitation | No | Irrelevant title/ abstract (reports from other methods) | |
| 230 | 2021 | Eidenbenz, David; Techel, Frank; Kottmann, Alexandre; Rousson, Valentin; Carron, Pierre-Nicolas; Albrecht, Roland; Pasquier, Mathieu | Survival probability in avalanche victims with long burial (>=60min): A retrospective study | Yes, only for systematic review | NA, since included | |
| 231 | 2001 | Eisenburger, P.; Czappek, G.; Sterz, F.; Vergeiner, G.; Losert, H.; Holzer, M.; Laggner, A. N. | Cardiac arrest patients in an alpine area during a six year period | No | Excluded at title/ abstract screening | |
| 232 | 2012 | Ekeland A, Rødven A, Heir S | Injury trends in recreational skiers and boarders in the 16-year period 1996-2012. | No | Irrelevant title/ abstract (reports from other methods) | |
| 233 | 2010 | Ekker, Ragnar; Engeset, Rune; Taurisano, Andrea; Schuler, Dagrun V.; Seierstad, Ivar AmbjÃ¸rn; Humstad, Tore; MyrabÃ¸, Steinar | Testing and Developing Methods for Avalanche Forecasting in Norway | No | Excluded at title/ abstract screening | |
| 234 | 2013 | Ekker, Ragnar; KvÃ¦rne, Kjetil; Os, Aslak; Humstad, Tore; Wartiainen, Anders; Eide, Vidar; Hansen, Ravi Kjell | RegObs - Public Database for Submitting and Sharing Observations | No | Excluded at title/ abstract screening | |
| 235 | 1974 | Eliakis E | La mort violente par avalanche. Mise au point m6dico16gale | No | Data already covered | |
| 236 | 1974 | Eliakis, E. | Violent death by avalanche. The medico legal aspects | No | No relevant statistics obtained | |
| 237 | 1974 | Elikas | LA MORT VIOLENTE PAR AVALANCHE | Yes, for meta-analysis and systematic review | NA, since included | |
| 238 | 2009 | Elnitsky, M. A.; Benoit, J. B.; Lopez-Martinez, G.; Denlinger, D. L.; Lee Jr, R. E. | Osmoregulation and salinity tolerance in the antarctic midge, belgica antarctica: seawater exposure confers enhanced tolerance to freezing and dehydration | No | Excluded at title/ abstract screening | |
| 239 | 2016 | Elsensohn, F. | Treatment of casualties in hostile environments: Emergency medicine in mountain sports | No | Excluded at title/ abstract screening | |

| Appendix. Data screening record. | | | | | | |
|---|---|---|---|---|---|---|
| **This table records inclusion and exclusion decisions (with reasons for exclusion) for 1031 studies/registries (750 unique ones from database search and 281 unique ones from reference screening and expert consultation); Reasons to exclude correspond to Fig 1 in the paper** | | | | | | |
| **Index** | **Publication Year** | **Author** | **Title** | **If included** | **Reason for exclusion** | **URL for grey included paper** |
| **240** | **2009** | Elsensohn, Fidel; Niederklapfer, Thomas; Ellerton, John; Swangard, Michael; Brugger, Hermann; Paal, Peter | Current status of medical training in mountain rescue in America and Europe | No | Excluded at title/ abstract screening | |
| **241** | **2012** | Emsden, Christopher | WORLD WATCH | No | Excluded at title/ abstract screening | |
| **242** | **2018** | Engeset, R. V.; Pfuhl, G.; Landrø, M.; Mannberg, A.; Hetland, A. | Communicating public avalanche warnings-what works? | No | Excluded at title/ abstract screening | |
| **243** | **2001** | Enrich, David | Digging out. (Cover story) | No | Excluded at title/ abstract screening | |
| **244** | **2014** | Enrione, G.; Roero, S.; Catuzzo, B.; Giardini, G.; Visetti, E. | Cardiac arrest in mountain setting (above 1800 M.A.S.L.): Two years' hems experience review in Aosta Valley, Italy | No | Excluded at title/ abstract screening | |
| **245** | **2009** | Erb, Christina | Avalanches Don't Discriminate | No | Excluded at title/ abstract screening | |
| **246** | **2001** | Erschbamer, B.; Kneringer, E.; Schlag, R. N. | Seed rain, soil seed bank, seedling recruitment, and survival of seedlings on a glacier foreland in the Central Alps | No | Irrelevant title/ abstract (reports from other methods) | |
| **247** | **2010** | Escande, SÃ©ba stien; LÃ©tang, Dominique | Is Going From Snow Avalanche Analysis to Decision Making Different for Snow Scientists or Practitioners? Overview of the Two Contexts. | No | Excluded at title/ abstract screening | |
| **248** | **1995** | Essberger, R. | Military surprise and the environment | No | Irrelevant title/ abstract (reports from other methods) | |
| **249** | **2006** | Evans, S. G. | Single-event landslides resulting from massive rock slope failure: Characterising their frequency and impact on society | No | Irrelevant title/ abstract (reports from other methods) | |
| **250** | **1997** | Evans, S. G. | Fatal landslides and landslide risk in Canada | No | Irrelevant title/ abstract (reports from other methods) | |
| **251** | **2009** | Evans, S. G.; Roberts, N. J.; Ischuk, A.; Delaney, K. B.; Morozova, G. S.; Tutubalina, O. | Landslides triggered by the 1949 Khait earthquake, Tajikistan, and associated loss of life | No | Excluded at title/ abstract screening | |
| **252** | **2021** | Evtushenko, N. A.; Beilin, A. K.; Kosykh, A. V.; Vorotelyak, E. A.; Gurskaya, N. G. | Keratins as an Inflammation Trigger Point in Epidermolysis Bullosa Simplex | No | Excluded at title/ abstract screening | |

**Appendix. Data screening record.**

**This table records inclusion and exclusion decisions (with reasons for exclusion) for 1031 studies/registries (750 unique ones from database search and 281 unique ones from reference screening and expert consultation); Reasons to exclude correspond to Fig 1 in the paper**

| Index | Publication Year | Author | Title | If included | Reason for exclusion | URL for grey included paper |
|---|---|---|---|---|---|---|
| 253 | 2009 | FÃ¶rster, M. | How Do Leadership Styles Cope With the Impact of Human Factors in Decision Making in Risky Terrain? | No | Excluded at title/ abstract screening | |
| 254 | 2014 | Facchetti, G.; Pozone, T.; Bianchi, G.; Bianchi, V.; Taraschi, F.; Marinangeli, F. | Rapid response system and avalanche: An innovative educational project based on the continuous quality improvement in abruzzo (Italy) | No | Excluded at title/ abstract screening | |
| 255 | 2021 | Faldesai, M.; Savoikar, P. P. | Monitoring, Control and Mapping of Landslides in Goa | No | Irrelevant title/ abstract (reports from other methods) | |
| 256 | 1994 | Falk M, Brugger H, Adler-Kastner L | Avalanche survival chances | No | No relevant data (full text examined, papers through other methods) | |
| 257 | 2012 | Fallon, Sean; Latosuo, Eeva | Know Before You Go: Adapting Youth Avalanche Education for Alaska | No | Excluded at title/ abstract screening | |
| 258 | 2019 | Fan, Xuanmei; Xu, Qiang; Scaringi, Gianvito; Zheng, Guang; Huang, Runqiu; Dai, Lanxin; Ju, Yuanzhen | The "long" runout rock avalanche in Pusa, China, on August 28, 2017: a preliminary report | No | Irrelevant title/ abstract (reports from other methods) | |
| 259 | 2020 | Farnell, Shauna | The Avalanche Didn't Kill Him. It Only Made Him Stronger | No | Irrelevant title/ abstract (reports from other methods) | |
| 260 | 2014 | Favier, P.; Eckert, N.; Bertrand, D.; Naaim, M. | Sensitivity of avalanche risk to vulnerability relations | No | Excluded at title/ abstract screening | |
| 261 | 2013 | Feistl, T.; Bebi, P.; Bartelt, P. | The Role of Slope Angle, Ground Roughness and Stauchwall Strength in the Formation of Glide-Snow Avalanches in Forest Gaps | No | Excluded at title/ abstract screening | |
| 262 | 2012 | Feistl, T.; Bebi, P.; Ì'uhler, Y. B.; Christen, M.; Teich, M.; Bartelt, P. | Stopping Behavior of Snow Avalanches in Forests | No | Excluded at title/ abstract screening | |
| 263 | 1990 | Fierro MF, Ongley JP | Blunt forces injuries. | No | Irrelevant title/ abstract (reports from other methods) | |
| 264 | 1996 | Filey B | The fact book about Mount Rainier | No | Irrelevant title/ abstract (reports from other methods) | |
| 265 | 2000 | Fink, K. S.; Willi, M. J.; Grissom, C. K.; Radwin, M. I.; Harmston, C. H. | Improving survival during snow burial in avalanches [5] (multiple letters) | No | No relevant statistics obtained | |

| **Appendix. Data screening record.** | | | | | | |
|---|---|---|---|---|---|---|
| **This table records inclusion and exclusion decisions (with reasons for exclusion) for 1031 studies/registries (750 unique ones from database search and 281 unique ones from reference screening and expert consultation); Reasons to exclude correspond to Fig 1 in the paper** | | | | | | |
| **Index** | **Publication Year** | **Author** | **Title** | **If included** | **Reason for exclusion** | **URL for grey included paper** |
| **266** | **1993** | Finkel, Michael | Six feet under | No | Excluded at title/ abstract screening | |
| **267** | **2013** | Flaherty, Erin J. | Media, Mountain Culture and the Identity Politics of Risk Recreation: A Media Discourse Analysis of Snowmobiling Avalanche Deaths in Western Canada | No | Excluded at title/ abstract screening | |
| **268** | **2012** | Flegenheimer, Matt | Winter Weather Brings Deaths and Disrupts Travel in Europe | No | Excluded at title/ abstract screening | |
| **269** | **2021** | Fleming, Z. D.; Pavlis, T. L.; Canalda, S. | Superposition of two kinematically distinct extensional phases in southern Death Valley: Implications for extensional tectonics | No | Excluded at title/ abstract screening | |
| **270** | **1996** | Fliri, F. | Hans Kinzl and the Innsbruck School of Population Geography | No | Irrelevant title/ abstract (reports from other methods) | |
| **271** | **2017** | Foster, T. O. M. | Cory Richards | No | Excluded at title/ abstract screening | |
| **272** | **2004** | Frangoulis, C.; Christou, E. D.; Hecq, J. H. | Comparison of marine copepod outfluxes: Nature, rate, fate and role in the carbon and nitrogen cycles | No | Excluded at title/ abstract screening | |
| **273** | **1990** | Fredenhagen, H. | Death in an avalanche | No | Excluded at title/ abstract screening | |
| **274** | **2016** | Fredlund, Beau | GRAVITY IN MIDDLE EARTH | No | Excluded at title/ abstract screening | |
| **275** | **2013** | Fredriksen | North-Norwegian avalanche victims: a retrospective observational study | Yes, for meta-analysis and systematic review | NA, since included | |
| **276** | **2006** | Fredston, Jill | Snow Falling on Skiers | No | Excluded at title/ abstract screening | |
| **277** | **2009** | Frolking, S.; Palace, M. W.; Clark, D. B.; Chambers, J. Q.; Shugart, H. H.; Hurtt, G. C. | Forest disturbance and recovery: A general review in the context of spaceborne remote sensing of impacts on aboveground biomass and canopy structure | No | Excluded at title/ abstract screening | |
| **278** | **2009** | Fruehauf, Florian; Heilig, Achim; Schneebeli, Martin; Fellin, Wolfgang; Scherzer, Otmar | Experiments and Algorithms to Detect Snow Avalanche Victims Using Airborne Ground-Penetrating Radar | No | Excluded at title/ abstract screening | |
| **279** | **2019** | Fuchs, S.; Keiler, M.; Sokratov, S. | Snow avalanches | No | Irrelevant title/ abstract (reports from other methods) | |
| **280** | **2022** | Fuchs, S.; Wenk, M.; Keiler, M. | Geomorphic Hazards in Austria | No | Excluded at title/ abstract screening | |

| Appendix. Data screening record. | | | | | | |
|---|---|---|---|---|---|---|
| **This table records inclusion and exclusion decisions (with reasons for exclusion) for 1031 studies/registries (750 unique ones from database search and 281 unique ones from reference screening and expert consultation); Reasons to exclude correspond to Fig 1 in the paper** | | | | | | |
| **Index** | **Publication Year** | **Author** | **Title** | **If included** | **Reason for exclusion** | **URL for grey included paper** |
| 281 | 1997 | Fukuoka, H.; Sassa, K.; Scarascia-Mugnozza, G. | Distribution of landslides triggered by the 1995 Hyogo-ken Nanbu earthquake and long runout mechanism of the Takarazuka golf course landslide | No | Excluded at title/ abstract screening | |
| 282 | 2020 | Furdada, Glòria; Margalef, Aina; Trapero, Laura; Pons, Marc; Areny, Francesc; Baró, Margaret; Reyes, Albert; Guinau, Marta | The Avalanche of Les Fonts d'Arinsal (Andorra): An Example of a Pure Powder, Dry Snow Avalanche | No | Excluded at title/ abstract screening | |
| 283 | 2010 | Furman, Nate; Shooter, Wynn; Schumann, Scott | The Roles of Heuristics, Avalanche Forecast, and Risk Propensity in the Decision Making of Backcountry Skiers | No | Excluded at title/ abstract screening | |
| 284 | 1995 | Furrer, M.; Erhart, S.; Frutiger, A.; Bereiter, H.; Leutenegger, A.; Rüedi, T. | Severe skiing injuries: a retrospective analysis of 361 patients including mechanism of trauma, severity of injury, and mortality | No | Excluded at title/ abstract screening | |
| 285 | 2013 | G, MartÃ; S, OrguÃ©; J, GavaldÃ; P, Oller; F, Carola; H, Serred; T, Arteta | Snow and Avalanche Association in Spain: A Merging of Professional and Amateur Experiences | No | Excluded at title/ abstract screening | |
| 286 | 2010 | Galvin, John | Survival Chronicles | No | Excluded at title/ abstract screening | |
| 287 | 2020 | Gao, Y.; Li, B.; Gao, H. Y.; Chen, L. C.; Wang, Y. F. | Dynamic characteristics of high-elevation and long-runout landslides in the Emeishan basalt area: a case study of the Shuicheng "7.23"landslide in Guizhou, China | No | Irrelevant title/ abstract (reports from other methods) | |
| 288 | 2017 | Gao, Y.; Yin, Y. P.; Li, B.; Feng, Z.; Wang, W. P.; Zhang, N.; Xing, A. G. | Characteristics and numerical runout modeling of the heavy rainfall-induced catastrophic landslide-debris flow at Sanxicun, Dujiangyan, China, following the Wenchuan Ms 8.0 earthquake | No | Irrelevant title/ abstract (reports from other methods) | |

| Appendix. Data screening record. | | | | | | |
|---|---|---|---|---|---|---|
| **This table records inclusion and exclusion decisions (with reasons for exclusion) for 1031 studies/registries (750 unique ones from database search and 281 unique ones from reference screening and expert consultation); Reasons to exclude correspond to Fig 1 in the paper** | | | | | | |
| **Index** | **Publication Year** | **Author** | **Title** | **If included** | **Reason for exclusion** | **URL for grey included paper** |
| 289 | 2017 | García Sellés C. | Variabilidad espacio-temporal de grandes aludes en el Pirineo Oriental según la estructura del manto nivoso y la circulación atmosférica. | No | Irrelevant title/ abstract (reports from other methods) | |
| 290 | 2018 | García-Hernández, C.; Ruiz-Fernández, J.; Sánchez-Posada, C.; Pereira, S.; Oliva, M. | An extreme event between the little ice age and the 20th century: The snow avalanche cycle of 1888 in the Asturian Massif (Northern Spain) | No | Excluded at title/ abstract screening | |
| 291 | 1986 | Gardner, J. S. | SNOW AS A RESOURCE AND HAZARD IN EARLY-TWENTIETH-CENTURY MINING, SELKIRK MOUNTAINS, BRITISH COLUMBIA | No | Excluded at title/ abstract screening | |
| 292 | 2007 | Gardner, J. S.; Dekens, J. | Mountain hazards and the resilience of social-ecological systems: Lessons learned in India and Canada | No | Excluded at title/ abstract screening | |
| 293 | 2021 | Garg, R. K.; Bhola, J.; Soni, S. K. | Healthcare monitoring of mountaineers by low power Wireless Sensor Networks | No | Excluded at title/ abstract screening | |
| 294 | 2020 | Gasser, B. | Y Equipment Became Better in Backcountry Skiing-Did Severity of Injuries Decrease? An Analysis from the Swiss Alps | No | Excluded at title/ abstract screening | |
| 295 | 2022 | Gasteiger, Lukas; Putzer, Gabriel; Unterpertinger, Regina; Cardini, Benno; Schneeberger, Stefan; Eschertzhuber, Stephan; Mair, Peter | Solid Organ Donation From Brain-dead Donors With Cardiorespiratory Arrest After Snow Avalanche Burial: A Retrospective Single-center Study | No | Excluded at title/ abstract screening | |
| 296 | 2021 | Gaume, J.; Puzrin, A. M. | Mechanisms of slab avalanche release and impact in the Dyatlov Pass incident in 1959 | No | Excluded at title/ abstract screening | |
| 297 | 2015 | Geisenberger, D.; Kramer, L.; Pircher, R.; Pollak, S. | Death by avalanche in the minor mountain range | Yes, only for systematic review | NA, since included | |
| 298 | 2015 | Geisenberger, Dorothee; Kramer, Lena; Pircher, Rebecca; Pollak, Stefan | Lawinentod im Mittelgebirge | No | Excluded at title/ abstract screening | |

| Appendix. Data screening record. | | | | | | |
|---|---|---|---|---|---|---|
| **This table records inclusion and exclusion decisions (with reasons for exclusion) for 1031 studies/registries (750 unique ones from database search and 281 unique ones from reference screening and expert consultation); Reasons to exclude correspond to Fig 1 in the paper** | | | | | | |
| **Index** | **Publication Year** | **Author** | **Title** | **If included** | **Reason for exclusion** | **URL for grey included paper** |
| 299 | 2014 | Geisler, Karl R. | Explaining Human Factors with Behavioral Economics | No | Excluded at title/abstract screening | |
| 300 | 2007 | Gennarelli TA, Wodzin E, editors. | Abbreviated injury scale 2005 | No | Irrelevant title/abstract (reports from other methods) | |
| 301 | 2002 | Genswein M, Harvey S | Search tactics in the presence of multiple burials. | No | Irrelevant title/abstract (reports from other methods) | |
| 302 | 2009 | Genswein, M. | Will a guest ever be able to save your life? | No | Excluded at title/abstract screening | |
| 303 | 2022 | Genswein, M.; Macias, D.; McIntosh, S.; Reiweger, I.; Hetland, A.; Paal, P. | AvaLife—A New Multi-Disciplinary Approach Supported by Accident and Field Test Data to Optimize Survival Chances in Rescue and First Aid of Avalanche Patients | No | Excluded at title/abstract screening | |
| 304 | 2018 | Germain, D.; Dagenais-Du-Fort, É; Lajeunesse, P.; Simard, M. | Dendrogeomorphic reconstruction of the seasonal timing and rainfall threshold for debris slide occurrence in eastern Canada | No | Excluded at title/abstract screening | |
| 305 | 2010 | Germain, D.; Hétu, B.; Filion, L. | Tree-Ring Based Reconstruction of Past Snow Avalanche Events and Risk Assessment in Northern Gaspé Peninsula (Québec, Canada) | No | Excluded at title/abstract screening | |
| 306 | 2014 | Germany, J. O. | Snowbound navigation: Mobile device interfaces for avalanche rescue | No | Excluded at title/abstract screening | |
| 307 | 2011 | Gibbins, Tim | Graveyard Shift | No | Excluded at title/abstract screening | |
| 308 | 2011 | Giesbrecht, G. G.; McDonald, G. K. | Exit Strategies and Safety Concerns for Machinery Occupants Following Ice Failure and Submersion | No | Excluded at title/abstract screening | |
| 309 | 2000 | Gilbert M, Busund R, Skagseth A, Nilsen PA, Solbø JP. | Resuscitation from accidental hypothermia of 13.7 degrees C with circulatory arrest. | No | Irrelevant title/abstract (reports from other methods) | |
| 310 | 2004 | Giolo SR | Turnbull's nonparametric estimator for intervalcensored data [technical report] | No | Irrelevant title/abstract (reports from other methods) | |

| **Appendix. Data screening record.** | | | | | | |
|---|---|---|---|---|---|---|
| **This table records inclusion and exclusion decisions (with reasons for exclusion) for 1031 studies/registries (750 unique ones from database search and 281 unique ones from reference screening and expert consultation); Reasons to exclude correspond to Fig 1 in the paper** | | | | | | |
| **Index** | **Publication Year** | **Author** | **Title** | **If included** | **Reason for exclusion** | **URL for grey included paper** |
| **311** | **2023** | Glòria Martí, Ricard Cabré, Íñigo Soteras, Ramón Arqué, Carles García-Sellés, Santiago Manguan | ANALYSIS OF 50 YEARS OF FATAL AVALANCHE ACCIDENTS IN THE CATALAN PYRENEES (1970-2020) | No | Data already covered | |
| **312** | **2018** | Goetz, Daniel; Latu, Vincent; Dufour, Anne; ColÃ©ou, CÃ©cile | On the use of forecasting tools during a numerous slab avalanches event in the northern French Alps in March 2018 | No | Excluded at title/abstract screening | |
| **313** | **2013** | Goodale, Gloria | Colorado avalanche: Unstable snow is a peril, even for the experienced | No | Excluded at title/abstract screening | |
| **314** | **2013** | Goode, J. R.; Buffington, J. M.; Tonina, D.; Isaak, D. J.; Thurow, R. F.; Wenger, S.; Nagel, D.; Luce, C.; Tetzlaff, D.; Soulsby, C. | Potential effects of climate change on streambed scour and risks to salmonid survival in snow-dominated mountain basins | No | Irrelevant title/abstract (reports from other methods) | |
| **315** | **2000** | Goodman, David | When the Mountain Falls | No | Irrelevant title/abstract (reports from other methods) | |
| **316** | **2014** | Grab, S. W.; Linde, J. H. | Mapping exposure to snow in a developing African context: implications for human and livestock vulnerability in Lesotho | No | Excluded at title/abstract screening | |
| **317** | **2016** | Grasegger, Katharina; Strapazzon, Giacomo; Procter, Emily; Brugger, Hermann; Soteras, Inigo | Avalanche Survival After Rescue With the RECCO Rescue System: A Case Report | No | Excluded at title/abstract screening | |
| **318** | **2021** | Grathwohl, A.; Hinz, P.; Burr, R.; Steiner, M.; Waldschmidt, C. | Experimental Study on the Detection of Avalanche Victims using an Airborne Ground Penetrating Synthetic Aperture Radar | No | Excluded at title/abstract screening | |
| **319** | **1987** | Gray D | Survival after burial in an avalanche | No | Excluded at title/abstract screening | |
| **320** | **2014** | Greene, Ethan M.; Jamieson, Bruce; Logan, Spencer | Fatal Occupational Injuries of Avalanche Workers in North America | No | Excluded at title/abstract screening | |
| **321** | **2006** | Greene, Ethan; Wiesinger, Thomas; Birkeland, Karl; ColÃ©ou, CÃ©cile; Jones, Alan; Statham, Grant | Fatal Avalanche Accidents and Forecasted Danger Levels: Patterns in the United States, Canada, Switzerland and France | No | Excluded at title/abstract screening | |

| **Appendix. Data screening record.** | | | | | | |
|---|---|---|---|---|---|---|
| **This table records inclusion and exclusion decisions (with reasons for exclusion) for 1031 studies/registries (750 unique ones from database search and 281 unique ones from reference screening and expert consultation); Reasons to exclude correspond to Fig 1 in the paper** | | | | | | |
| **Index** | **Publication Year** | **Author** | **Title** | **If included** | **Reason for exclusion** | **URL for grey included paper** |
| **322** | | Greene, K.; Hendrikx, J.; Johnson, J. | The Impact of Avalanche Education on Risk Perception, Confidence, and Decision-Making among Backcountry Skiers | No | Excluded at title/ abstract screening | |
| **323** | **2004** | Grímsdóttir, Harpa | Avalanche risk management in backcountry skiing operations | No | Irrelevant title/ abstract (reports from other methods) | |
| **324** | **2006** | Grissom CK, Thomas F, James B. | Medical helicopters in wilderness search and rescue operations. | No | Irrelevant title/ abstract (reports from other methods) | |
| **325** | **2011** | Grissom, C. K. | Rewarming" an Important Issue from the Cold: Simulated Avalanche Survival and the Physiology of Afterdrop Reply | No | Excluded at title/ abstract screening | |
| **326** | **2011** | Grissom, C. K. | Lessons learned from avalanche survival patterns | No | All relevant statistics were cited elsewhere | |
| **327** | **2008** | Grissom, C. K.; McAlpine, J. C.; Harmston, C. H.; Radwin, M. I.; Giesbrecht, G. G.; Scholand, M. B.; Morgan, J. S. | Hypercapnia effect on core cooling and shivering threshold during snow burial | No | Excluded at title/ abstract screening | |
| **328** | **2000** | Grissom, C. K.; Radwin, M. I.; Harmston, C. H. | Improving survival during snow burial in avalanches | No | No relevant statistics obtained | |
| **329** | **2000** | Grissom, C. K.; Radwin, M. I.; Harmston, C. H. | Improving survival during snow burial in avalanches - Reply | No | Excluded at title/ abstract screening | |
| **330** | **2004** | Grissom, C. K.; Radwin, M. I.; Scholand, M. B.; Harmston, C. H.; Muetterties, M. C.; Bywater, T. J. | Hypercapnia increases core temperature cooling rate during snow burial | No | Excluded at title/ abstract screening | |
| **331** | **2004** | Grissom, Colin K.; Harmston, Chris H.; Radwin, Martin I.; Scholand, Mary Beth; McAlpine, John C.; Morgan, J. Scott; Bywater, Tim J.; Grissom, Abigail W. | Hypothermia During Avalanche Burial | No | Excluded at title/ abstract screening | |
| **332** | **2000** | Grissom, Colin K.; Radwin, Martin I.; Harmston, Chris H.; Hirshberg, Ellie L.; Crowley, Thomas J.; Grissom, C. K.; Radwin, M. I.; Harmston, C. H.; Hirshberg, E. L.; Crowley, T. J. | Respiration during snow burial using an artificial air pocket | No | Irrelevant title/ abstract (reports from other methods) | |

**Appendix. Data screening record.**

**This table records inclusion and exclusion decisions (with reasons for exclusion) for 1031 studies/registries (750 unique ones from database search and 281 unique ones from reference screening and expert consultation); Reasons to exclude correspond to Fig 1 in the paper**

| Index | Publication Year | Author | Title | If included | Reason for exclusion | URL for grey included paper |
|---|---|---|---|---|---|---|
| 333 | 2021 | Gross, M.; Jackowski, C.; Schön, C. A. | Fatalities associated with ski touring and freeriding: A retrospective analysis from 2001 to 2019 | Yes, for meta-analysis and systematic review | NA, since included | |
| 334 | 2007 | Grosse, Alexandra B.; Grosse, Claudia A.; Steinbach, Lynne S.; Zimmermann, Heinz; Anderson, Suzanne | Imaging findings of avalanche victims | Yes, only for systematic review | NA, since included | |
| 335 | 1989 | Grossman, M. D.; Saffle, J. R.; Thomas, F.; Tremper, B. | Avalanche trauma | Yes, for meta-analysis and systematic review | NA, since included | |
| 336 | 2020 | Groves, Matthew R.; Varley, Peter J. | Critical mountaineering decisions: technology, expertise and subjective risk in adventurous leisure | No | Excluded at title/abstract screening | |
| 337 | 2001 | Gruber, U.; Margreth, S. | Winter 1999: A valuable test of the avalanche-hazard mapping procedure in Switzerland | No | Excluded at title/abstract screening | |
| 338 | 2007 | Guest, Bernard; Niemi, Nathan; Wernicke, Brian | Stateline fault system: A new component of the Miocene-Quaternary Eastern California shear zone | No | Excluded at title/abstract screening | |
| 339 | 2011 | Guly, Henry | History of accidental hypothermia | No | Excluded at title/abstract screening | |
| 340 | 1982 | Gupta SM, Chandra J, Dogra TD | Blunt force lesions related to the heights of a fall | No | Irrelevant title/abstract (reports from other methods) | |
| 341 | 1995 | Gürer, I.; Tunçel, H.; Yavaş, O. M.; Erenbilge, T.; Sayin, A. | Snow avalanche incidents in north-western Anatolia, Turkey during December 1992 | No | Irrelevant title/abstract (reports from other methods) | |
| 342 | 2013 | Guy, Zachary M.; Birkeland, Karl W. | Relating complex terrain to potential avalanche trigger locations | No | Excluded at title/abstract screening | |
| 343 | 2008 | Habermann, Marijke; Schweizer, Jürg; Jamieson, J. Bruce | Influence of snowpack layering on human-triggered snow slab avalanche release | No | Excluded at title/abstract screening | |
| 344 | 2015 | Haeberli, Wilfried; Whiteman, Colin A.; Shroder, John F. | Snow and ice-related hazards, risks and disasters | No | Irrelevant title/abstract (reports from other methods) | |
| 345 | 2005 | Haegeli P | Winter backcountry use trend estimates for western Canada. | | Irrelevant title/abstract (reports from other methods) | |

| Appendix. Data screening record. | | | | | | |
|---|---|---|---|---|---|---|
| This table records inclusion and exclusion decisions (with reasons for exclusion) for 1031 studies/registries (750 unique ones from database search and 281 unique ones from reference screening and expert consultation); Reasons to exclude correspond to Fig 1 in the paper | | | | | | |
| **Index** | **Publication Year** | **Author** | **Title** | **If included** | **Reason for exclusion** | **URL for grey included paper** |
| 346 | 2020 | Haegeli, P.; Rupf, R.; Karlen, B. | Do avalanche airbags lead to riskier choices among backcountry and out-of-bounds skiers? | No | Excluded at title/ abstract screening | |
| 347 | 2020 | Haegeli, P.; Strong-Cvetich, L. R. | Using discrete choice experiments to examine the stepwise nature of avalanche risk management decisions-An example from mountain snowmobiling | No | Excluded at title/ abstract screening | |
| 348 | 2010 | Haegeli, Pascal; Falk, Markus; Brugger, Hermann; Etter, Hans-JÃ¼rg; Boyd, Jeff | A Comparison of Avalanche Survival Patterns in Canada and Switzerland | No | Excluded at title/ abstract screening | |
| 349 | 2011 | Haegeli, Pascal; Falk, Markus; Brugger, Hermann; Etter, Hans-Jürg; Boyd, Jeff | Comparison of avalanche survival patterns in Canada and Switzerland | Yes, only for systematic review | NA, since included | |
| 350 | 2012 | Haegeli, Pascal; Falk, Markus; Klassen, Karl | Linking Avalanche Problems to Avalanche Dangerâ€" A First Statistical Examination of the Conceptual Model of Avalanche Hazard | No | Excluded at title/ abstract screening | |
| 351 | 2014 | Haladuick, Shane; Schirmer, Michael; Jamieson, Bruce | What do Field Observations tell us about Avalanche Danger? | No | Excluded at title/ abstract screening | |
| 352 | 2016 | Hallandvik, Linda; Aadland, Eivind; Vikene, Odd Lennart | Terrain Classification of Norwegian Slab Avalanche Accidents | No | Excluded at title/ abstract screening | |
| 353 | 1996 | Haller, H. | Predation and accidents among alpine ibex Capra ibex in the Engadine | No | Irrelevant title/ abstract (reports from other methods) | |
| 354 | 2018 | Hancock, Holt; Prokop, Alexander; Eckerstorfer, Markus; Hendrikx, Jordy | Combining high spatial resolution snow mapping and meteorological analyses to improve forecasting of destructive avalanches in Longyearbyen, Svalbard | No | Excluded at title/ abstract screening | |
| 355 | 2011 | Hanno Bilek, Wurtl | Zum Tode verurteilt! Aktuelle Daten zum Traumatod in der Lawine | No | Excluded at title/ abstract screening | |
| 356 | 2011 | Hanno Bilek, Wurtl | Zum Tode verurteilt! Aktuelle Daten zum Traumatod in der Lawine | Yes, for meta-analysis and systematic review | NA, since included | |

**Appendix. Data screening record.**

**This table records inclusion and exclusion decisions (with reasons for exclusion) for 1031 studies/registries (750 unique ones from database search and 281 unique ones from reference screening and expert consultation); Reasons to exclude correspond to Fig 1 in the paper**

| Index | Publication Year | Author | Title | If included | Reason for exclusion | URL for grey included paper |
|---|---|---|---|---|---|---|
| 357 | 2014 | Hansen, B. B.; Isaksen, K.; Benestad, R. E.; Kohler, J.; Pedersen, Å Ø; Loe, L. E.; Coulson, S. J.; Larsen, J. O.; Varpe, Ø | Warmer and wetter winters: Characteristics and implications of an extreme weather event in the High Arctic | No | Excluded at title/ abstract screening | |
| 358 | 1986 | Harries M | Drowning and near drowning | No | Irrelevant title/ abstract (reports from other methods) | |
| 359 | 2002 | Harvey S, Signorell C, Genswein M. | avalanche accident | | Irrelevant title/ abstract (reports from other methods) | |
| 360 | 2002 | Harvey S, Signorell C. | Avalanche incidents in backcountry terrain of the Swiss Alps: New investigations with a 30 years database. | No | Irrelevant title/ abstract (reports from other methods) | |
| 361 | 2017 | Hatchett, Benjamin J.; Burak, Susan; Rutz, Jonathan J.; Oakley, Nina S.; Bair, Edward H.; Kaplan, Michael L. | Avalanche Fatalities during Atmospheric River Events in the Western United States | No | Excluded at title/ abstract screening | |
| 362 | 2021 | Hatwal, P.; Bhaisora, C. P.; Devassy, S. | White death: Avalanche fatality in nanda devi mountain | Yes, only for systematic review | NA, since included | |
| 363 | 1986 | Haug AN, Schroen IL, Berntsen P, Tellefsen T, Husby S, Due J, Lied K, Hansen G, Collett M | Skredulykken i Vassdalen 5. | No | Irrelevant title/ abstract (reports from other methods) | |
| 364 | 2006 | Haupt, Jennifer | Trapped! | No | Excluded at title/ abstract screening | |
| 365 | 2018 | Hauser, Christine | Friends Save Boarder Caught in Avalanche | No | Excluded at title/ abstract screening | |
| 366 | 2018 | Haverkamp FJC, Giesbrecht GG, Tan ECTH, . | The prehospital management of hypothermia - An up-to-date overview | No | Irrelevant title/ abstract (reports from other methods) | |
| 367 | 2008 | Hawley, Patrick | MORAL ABSOLUTISM DEFENDED | No | Excluded at title/ abstract screening | |
| 368 | 2018 | Haye, L.; Boutroy, E.; Soulé, B. | Effectiveness of avalanche airbag facing the risk of burial : a systematic review (1996-2016) | No | No relevant statistics obtained | |
| 369 | 2008 | He, F.; Xu, Y. N.; Chen, H. Q.; Zhang, J. H. | Present status of mine geohazards in the northwest region of China and characteristics of their temporal-spatial distribution | No | Irrelevant title/ abstract (reports from other methods) | |

## Appendix. Data screening record.

**This table records inclusion and exclusion decisions (with reasons for exclusion) for 1031 studies/registries (750 unique ones from database search and 281 unique ones from reference screening and expert consultation); Reasons to exclude correspond to Fig 1 in the paper**

| Index | Publication Year | Author | Title | If included | Reason for exclusion | URL for grey included paper |
|---|---|---|---|---|---|---|
| 370 | 2022 | He, Kun; Liu, Bo; Hu, Xiewen; Zhou, Ruichen; Xi, Chuanjie; Ma, Guotao; Han, Mei; Li, You; Luo, Gang | Rapid Characterization of Landslide-Debris Flow Chains of Geologic Hazards Using Multi-method Investigation: Case Study of the Tiejiangwan LDC | No | Irrelevant title/ abstract (reports from other methods) | |
| 371 | 2003 | Hearns S | The Scottish mountain rescue casualty study | No | Irrelevant title/ abstract (reports from other methods) | |
| 372 | 2020 | Heimtun, B.; Jacobsen, J. K. S. | Withstanding winter vulnerabilities: A way of life in a northern seaside community | No | Excluded at title/ abstract screening | |
| 373 | 2021 | Heinrich, Bernd | Redpoll Winter Games | No | Excluded at title/ abstract screening | |
| 374 | 2008 | Heller, V. | Landslide generated impulse waves: Prediction of near field characteristics | No | Excluded at title/ abstract screening | |
| 375 | 1992 | Hendricks, P.; Norment, C. J. | EFFECTS OF A SEVERE SNOWSTORM ON SUB-ALPINE AND ALPINE POPULATIONS OF NESTING AMERICAN PIPITS | No | Irrelevant title/ abstract (reports from other methods) | |
| 376 | 2016 | Hendrikx, J.; Johnson, J.; Shelly, C. | Using GPS tracking to explore terrain preferences of heli-ski guides | No | Excluded at title/ abstract screening | |
| 377 | 2014 | Hendrikx, Jordy; Johnson, Jerry | Using Global Crowd-Sourced Data to Understand Travel Behavior in Avalanche Terrain | No | Excluded at title/ abstract screening | |
| 378 | 2022 | Hendrikx, Jordy; Johnson, Jerry; Mannberg, Andrea | Tracking decision-making of backcountry users using GPS tracks and participant surveys | No | Excluded at title/ abstract screening | |
| 379 | 2011 | Hermann, Brugger; Peter, Paal; Jeff, Boyd | Prehospital Resuscitation of the Buried Avalanche Victim | No | All relevant statistics were cited elsewhere | |
| 380 | 2014 | Hermanns, R. L.; Oppikofer, T.; Roberts, N. J.; Sandøy, G. | Catalogue of historical displacement waves and landslide-triggered tsunamis in norway | No | Excluded at title/ abstract screening | |
| 381 | 2011 | Hétu, B.; Brown, K.; Germain, D. | Fatal avalanche accidents in Québec (Canada) 1825 to 2009 | No | No relevant statistics obtained | |
| 382 | 1976 | Hewel, T.; Mang, W. R.; Maurer, P. C.; Zimmermann, Th | Fatal and severe skiing accidents | No | Irrelevant title/ abstract (reports from other methods) | |
| 383 | 2003 | Hewitt, Ben | Frostbite Nation | No | Excluded at title/ abstract screening | |

**Appendix. Data screening record.**

**This table records inclusion and exclusion decisions (with reasons for exclusion) for 1031 studies/registries (750 unique ones from database search and 281 unique ones from reference screening and expert consultation); Reasons to exclude correspond to Fig 1 in the paper**

| Index | Publication Year | Author | Title | If included | Reason for exclusion | URL for grey included paper |
|---|---|---|---|---|---|---|
| 384 | 1998 | Hewitt, K. | Catastrophic landslides and their effects on the Upper Indus streams, Karakoram Himalaya, northern Pakistan | No | Excluded at title/ abstract screening | |
| 385 | 1992 | Hewitt, K. | Mountain hazards | No | Irrelevant title/ abstract (reports from other methods) | |
| 386 | 2014 | Hewitt, K. | Landforms of the Karakoram Glacierised Areas | No | Irrelevant title/ abstract (reports from other methods) | |
| 387 | 1985 | Heywood, D. I.; Tufnell, L. | Snow avalanche hazards in the Glen Nevis and Glen Coe areas of Scotland | No | Excluded at title/ abstract screening | |
| 388 | 2012 | Hidding, B.; Klaassen, M.; de Boer, T.; de Vries, P. P.; Nolet, B. A. | Aquatic plant shows flexible avoidance by escape from tuber predation by swans | No | Excluded at title/ abstract screening | |
| 389 | 2013 | Highland, L.; Sun, P. | Environmental impact of the landslides caused by the 12 May 2008, Wenchuan, China earthquake | No | Irrelevant title/ abstract (reports from other methods) | |
| 390 | 2000 | Hilderbrand, G. V.; Lewis, L. L.; Larrivee, J.; Farley, S. D. | A denning Brown Bear, Ursus arctos, sow and two cubs killed in an avalanche on the Kenai Peninsula, Alaska | No | Irrelevant title/ abstract (reports from other methods) | |
| 391 | 2011 | Hirota, T.; Usuki, K.; Hayashi, M.; Nemoto, M.; Iwata, Y.; Yanai, Y.; Yazaki, T.; Inoue, S. | Soil frost control: Agricultural adaptation to climate variability in a cold region of Japan | No | Excluded at title/ abstract screening | |
| 392 | 2021 | Höch A, Zeidler S, Pieroh P, et al. | Trends and efficacy of external emergency stabilization of pelvic ring fractures: results from the German pelvic trauma registry. | No | Irrelevant title/ abstract (reports from other methods) | |
| 393 | 2016 | Hogg, A. J.; Jóhannesson, T. | Avalanche defence schemes | No | Excluded at title/ abstract screening | |
| 394 | 2007 | Hohlrieder, M.; Brugger, H.; Schubert, H. M.; Pavlic, M.; Ellerton, J.; Mair, P. | Pattern and severity of injury in avalanche victims | Yes, for meta-analysis and systematic review | NA, since included | |
| 395 | 2005 | Hohlrieder, Matthias; Mair, Peter; Wuertl, Walter; Brugger, Hermann | The impact of avalanche transceivers on mortality from avalanche accidents | No | No relevant statistics obtained | |

**Appendix. Data screening record.**

**This table records inclusion and exclusion decisions (with reasons for exclusion) for 1031 studies/registries (750 unique ones from database search and 281 unique ones from reference screening and expert consultation); Reasons to exclude correspond to Fig 1 in the paper**

| Index | Publication Year | Author | Title | If included | Reason for exclusion | URL for grey included paper |
|---|---|---|---|---|---|---|
| 396 | 2007 | Holler, P. | Avalanche hazards and mitigation in Austria: a review | No | Excluded at title/ abstract screening | |
| 397 | 2017 | Höller, P. | Avalanche accidents and fatalities in Austria since 1946/47 with special regard to tourist avalanches in the period 1981/82 to 2015/16 | No | No relevant statistics obtained | |
| 398 | 2009 | Höller, P. | Avalanche cycles in Austria: An analysis of the major events in the last 50 years | No | Excluded at title/ abstract screening | |
| 399 | 2012 | Holtmeier, F. K.; Broll, G. | Landform influences on treeline patchiness and dynamics in a changing climate | No | Excluded at title/ abstract screening | |
| 400 | 2019 | Horáková, L.; Sýkora, K.; Sieger, L.; Roubík, K. | Breathing experiments into the simulated avalanche snow: Medical and technical issues of the outdoor breathing trials | No | Irrelevant title/ abstract (reports from other methods) | |
| 401 | 2010 | Horgen A | Kulturhistoriske perspektiver på vinterfriluftslivet, i boka Friluftslivsveiledning vinterstid. Høgskoleforlaget | No | Irrelevant title/ abstract (reports from other methods) | |
| 402 | 2020 | Huey, Raymond B.; Carroll, Cody; Salisbury, Richard; Wang, Jane-Ling | Mountaineers on Mount Everest: Effects of age, sex, experience, and crowding on rates of success and death | No | Excluded at title/ abstract screening | |
| 403 | 2009 | Huggel, Christian | Recent extreme slope failures in glacial environments: effects of thermal perturbation | No | Excluded at title/ abstract screening | |
| 404 | 2018 | Hughes, P. D. | Little ice age glaciers and climate in the mediterranean mountains: A new analysis | No | Excluded at title/ abstract screening | |
| 405 | 2010 | Hughes, P. D. | Little Ice Age glaciers in the Balkans: Low altitude glaciation enabled by cooler temperatures and local topoclimatic controls | No | Irrelevant title/ abstract (reports from other methods) | |

**Appendix. Data screening record.**

**This table records inclusion and exclusion decisions (with reasons for exclusion) for 1031 studies/registries (750 unique ones from database search and 281 unique ones from reference screening and expert consultation); Reasons to exclude correspond to Fig 1 in the paper**

| Index | Publication Year | Author | Title | If included | Reason for exclusion | URL for grey included paper |
|---|---|---|---|---|---|---|
| 406 | 2016 | Hull, Claire M.; Rajendran, Dévan; Fernandez Barnes, Arturo | Deep Vein Thrombosis and Pulmonary Embolism in a Mountain Guide: Awareness, Diagnostic Challenges, and Management Considerations at Altitude | No | Excluded at title/ abstract screening | |
| 407 | 2013 | Hungerer, S.; Ebenhoch, M.; Geiser, T.; Buhren, V. | Accidental, profound hypothermia in mountain rescue emergencies | No | No relevant statistics obtained | |
| 408 | 2014 | Hüsler, F.; Jonas, T.; Riffler, M.; Musial, J. P.; Wunderle, S. | A satellite-based snow cover climatology (1985–2011) for the European Alps derived from AVHRR data | No | Irrelevant title/ abstract (reports from other methods) | |
| 409 | 1979 | Hutchins GM, Silverman KJ | Pathology of the stone heart syndrome | No | Irrelevant title/ abstract (reports from other methods) | |
| 410 | 1991 | Huzinec, M.; Hauser, S. | Nine perish as a fatal avalanche turns heli-skiing into a skiing hell | No | Excluded at title/ abstract screening | |
| 411 | 2021 | Ibor, C. S.; Belles, J. F. M.; Garcia, A. R. | Reconstruction of a debris flow: the disaster of Marines (Valencia) of October 1957 | No | Excluded at title/ abstract screening | |
| 412 | 2016 | Idell-Sassi, L.; Stickel, J. R.; Murphy, M.; Carter, P. | Expanding the road weather information system for avalanche support | No | Excluded at title/ abstract screening | |
| 413 | 2008 | Ikeda, Shinji; Wakabayashi, Ryuzo | A study of snow climates in The Japan Alps | No | Irrelevant title/ abstract (reports from other methods) | |
| 414 | 2021 | İliklerden, Duygu Mergan; Kalaycı, Tolga; Karacaoglu, İsmail Can | The evaluation of traumas of victims in Avalanche disasters in Van province, Turkey on February 4-5, 2020 | No | Study a single cause of death | |
| 415 | 2003 | Intini, John | DEATH IN THE BACKCOUNTRY | No | No relevant statistics obtained | |
| 416 | 2015 | Iribarren Anacona, P.; Mackintosh, A.; Norton, K. P. | Hazardous processes and events from glacier and permafrost areas: Lessons from the Chilean and Argentinean Andes | No | Irrelevant title/ abstract (reports from other methods) | |
| 417 | 2002 | Irvin, Dave | Avalanche accidents in aotearoa | Yes, for meta-analysis and systematic review | NA, since included | |
| 418 | 2016 | Ita, Kevin | Percutaneous penetration of anticancer agents: Past, present and future | No | Excluded at title/ abstract screening | |

**Appendix. Data screening record.**

**This table records inclusion and exclusion decisions (with reasons for exclusion) for 1031 studies/registries (750 unique ones from database search and 281 unique ones from reference screening and expert consultation); Reasons to exclude correspond to Fig 1 in the paper**

| Index | Publication Year | Author | Title | If included | Reason for exclusion | URL for grey included paper |
|---|---|---|---|---|---|---|
| 419 | 2012 | Iverson, R. M. | Mechanics of debris flows and rock avalanches | No | Excluded at title/ abstract screening | |
| 420 | 2010 | Izumi | Japanese Avalanche Disaster Database | No | Irrelevant title/ abstract (reports from other methods) | |
| 421 | 1997 | Izumi, K.; Kobayashi, S.; Yano, K.; Endo, Y.; Ohzeki, Y.; Watanabe, S. | Statistics on avalanche accidents in the central part of Japan (1900-1989) | No | No relevant statistics obtained | |
| 422 | 1994 | J.G | Lofty agenda | No | Excluded at title/ abstract screening | |
| 423 | 2009 | Jaedicke, C.; Lied, K.; Kronholm, K. | Integrated database for rapid mass movements in Norway | No | Excluded at title/ abstract screening | |
| 424 | 2008 | Jaedicke, C.; Solheim, A.; Blikra, L. H.; Stalsberg, K.; Sorteberg, A.; Aaheim, A.; Kronholm, K.; Vikhamar-Schuler, D.; Isaksen, K.; Sletten, K.; Kristensen, K.; Barstad, I.; Melchiorre, C.; Høydal, Ø A.; Mestl, H. | Spatial and temporal variations of Norwegian geohazards in a changing climate, the GeoExtreme Project | No | Excluded at title/ abstract screening | |
| 425 | 2015 | Jakubis, M. | Winter recreation and avalanche danger in the western tatras | No | Irrelevant title/ abstract (reports from other methods) | |
| 426 | 2017 | Jakubis, M. | DEVELOPMENT OF FATAL ACCIDENTS DURING THE LEISURE ACTIVITIES IN THE HIGH TATRAS (SLOVAKIA) IN TWENTIETH CENTURY | No | Irrelevant title/ abstract (reports from other methods) | |
| 427 | 1996 | Jamieson B, Geldsetzer T. | Avalanche Accidents in Canada, Vol. 4 | No | Data already covered | |
| 428 | 1990 | Jamieson B, Geldsetzer T. | Avalanche Accidents in Canada, Vol. 3 | No | Data already covered | |
| 429 | 1980 | Jamieson B, Geldsetzer T. | Avalanche Accidents in Canada, Vol. 2 | No | Data already covered | |
| 430 | 1979 | Jamieson B, Geldsetzer T. | Avalanche Accidents in Canada, Vol. 1 | No | Data already covered | |
| 431 | 2015 | Jamieson, B.; Jones, A. S. T. | The effect of under-reporting of non-fatal involvements in snow avalanches on vulnerability | No | Excluded at title/ abstract screening | |
| 432 | 2009 | Jamieson, B.; Schweizer, J.; Shea, C. | Simple calculations of avalanche risk for backcountry skiing | No | Excluded at title/ abstract screening | |

**Appendix. Data screening record.**

**This table records inclusion and exclusion decisions (with reasons for exclusion) for 1031 studies/registries (750 unique ones from database search and 281 unique ones from reference screening and expert consultation); Reasons to exclude correspond to Fig 1 in the paper**

| Index | Publication Year | Author | Title | If included | Reason for exclusion | URL for grey included paper |
|---|---|---|---|---|---|---|
| 433 | 2002 | Jamieson, B.; Stethem, C. | Snow avalanche hazards and management in Canada: Challenges and progress | No | Excluded at title/ abstract screening | |
| 434 | 2012 | Jamieson, Bruce; Jones, Alan S. T. | Vulnerability: Caught in an Avalanche â€“ Then What are the Odds? | No | Excluded at title/ abstract screening | |
| 435 | 2009 | Jamieson, Bruce; Schweizer, JÃ¼rg; Shea, Cora | Simple Calculations of Avalanche Risk for Backcountry Skiing | No | Excluded at title/ abstract screening | |
| 436 | 2007 | Jamieson, C. J.; Schaerer, P. A.; Jamieson, Bruce; Haegeli, Pascal; Gauthier, Dave | Avalanche accidents in Canada, vol 5 | Yes, for meta-analysis and systematic review | NA, since included | |
| 437 | 1992 | Jamieson, J. B.; Johnston, C. D. | Snowpack characteristics associated with avalanche accidents | No | Excluded at title/ abstract screening | |
| 438 | 2000 | Jamieson, J. B.; Schweizer, J. | Texture and strength changes of buried surface-hoar layers with implications for dry snow-slab avalanche release | No | Excluded at title/ abstract screening | |
| 439 | 2023 | Japan Association for Skiing Safety | Ski Resort Injury Report | No | Irrelevant title/ abstract (reports from other methods) | |
| 440 | 2016 | Jekich, Brian M.; Drake, Brandy D.; Nacht, Jacob Y.; Nichols, Andrew; Ginde, Adit A.; Davis, Christopher B. | Avalanche Fatalities in the United States: A Change in Demographics | No | No relevant statistics obtained | |
| 441 | 2016 | Jenkins, M.; Young, D.; Gale, K.; Ieee | Enhancing Hazard Awareness with a Mobile Application for High Risk, High Consequence Avalanche Terrain Decisions | No | Irrelevant title/ abstract (reports from other methods) | |
| 442 | 2000 | Jenkins, McKay | Avalanche | No | Irrelevant title/ abstract (reports from other methods) | |
| 443 | 2021 | Jiang, Ruochen; Zhang, Limin; Peng, Dalei; He, Xin; He, Jian | The Landslide Hazard Chain in the Tapovan of the Himalayas on 7 February 2021 | No | Excluded at title/ abstract screening | |
| 444 | 1994 | Jibson, R. W.; Prentice, C. S.; Borissoff, B. A.; Rogozhin, E. A.; Langer, C. J. | SOME OBSERVATIONS OF LANDSLIDES TRIGGERED BY THE 29-APRIL-1991 RACHA EARTHQUAKE, REPUBLIC-OF-GEORGIA | No | Irrelevant title/ abstract (reports from other methods) | |

**Appendix. Data screening record.**

**This table records inclusion and exclusion decisions (with reasons for exclusion) for 1031 studies/registries (750 unique ones from database search and 281 unique ones from reference screening and expert consultation); Reasons to exclude correspond to Fig 1 in the paper**

| Index | Publication Year | Author | Title | If included | Reason for exclusion | URL for grey included paper |
|---|---|---|---|---|---|---|
| 445 | 2009 | Jindal, A. K. | The Highest Battlefield of the World : Medical Problems and Solutions | No | Excluded at title/ abstract screening | |
| 446 | 2013 | Johnson, A. C.; Yeakley, J. A. | Wood microsites at timberline-alpine meadow borders: Implications for conifer seedling regeneration and alpine meadow conifer invasion | No | Excluded at title/ abstract screening | |
| 447 | 1988 | Johnson, B.; Cooper, J. | The avalanche death of a friend has Prince | No | Excluded at title/ abstract screening | |
| 448 | 1987 | Johnson, E. A. | The relative importance of snow avalanche disturbance and thinning on canopy plant populations | No | Excluded at title/ abstract screening | |
| 449 | 2020 | Johnson, J.; Mannberg, A.; Hendrikx, J.; Hetland, A.; Stephensen, M. | Rethinking the heuristic traps paradigm in avalanche education: Past, present and future | No | Excluded at title/ abstract screening | |
| 450 | 2021 | Johnson, Jerry; Hendrikx, Jordy | Using Citizen Science to Document Terrain Use and Decision-Making of Backcountry Users | No | Excluded at title/ abstract screening | |
| 451 | 2005 | Johnson, Kirk | Skiers Risk Answering the Call of Their Wild Side. (Cover story) | No | Excluded at title/ abstract screening | |
| 452 | 2007 | Johnson, Rich | Swept Away | No | Excluded at title/ abstract screening | |
| 453 | 2001 | Johnson, S. M.; Johnson, A. C.; Barton, R. G. | Avalanche trauma and closed head injury: adding insult to injury | Yes, for meta-analysis and systematic review | NA, since included | |
| 454 | 2008 | Jonas, T.; Geiger, F.; Jenny, H. | Mortality pattern of the Alpine chamois: The influence of snow-meteorological factors | No | No relevant statistics obtained | |
| 455 | 2019 | Jones, Joshua N.; Boulton, Sarah J.; Bennett, Georgina L.; Whitworth, Michael R. Z.; Stokes, Martin | Investigating the landslide susceptibility of a glacial/periglacial landscape, Langtang Valley, Nepal | No | Excluded at title/ abstract screening | |
| 456 | 2014 | Joshi, P. K.; Sachdeva, K.; Joshi, A. K. | Adaptation frameworks for climate change-Eloquent to himalayan ecosystems | No | Excluded at title/ abstract screening | |

**Appendix. Data screening record.**

**This table records inclusion and exclusion decisions (with reasons for exclusion) for 1031 studies/registries (750 unique ones from database search and 281 unique ones from reference screening and expert consultation); Reasons to exclude correspond to Fig 1 in the paper**

| Index | Publication Year | Author | Title | If included | Reason for exclusion | URL for grey included paper |
|---|---|---|---|---|---|---|
| 457 | 2001 | Joyce, Peter; Poff, Raymond | Daring To Be Different! Proceedings of the Annual International Conference on Outdoor Recreation and Education (ICORE) (15th, Pocatello, Idaho, November 6-11, 2001) | No | Excluded at title/ abstract screening | |
| 458 | 1996 | Joyce, Peter; Watters, Ron; Idaho State Univ, Pocatello; Association of Outdoor, Recreation; Education, Boulder C. O. | Proceedings of the 1992 and 1993 Conferences on Outdoor Recreation (Calgary, Alberta, Canada, November 12-14, 1992; Corvallis, Oregon, November 11-13, 1993) | No | Excluded at title/ abstract screening | |
| 459 | 2016 | Kaczka, Ryszard J.; Spyt, Barbara; Janecka, Karolina; Lempa, MichaŁ; RĄCzkowska, Zofia | WHAT CAN WE LEARN FROM ARCHIVE RECORDS OF SNOW AVALANCHES IN THE TATRA MOUNTAINS? | No | Excluded at title/ abstract screening | |
| 460 | 2004 | Kajimoto, T.; Daimaru, H.; Okamoto, T.; Otani, T.; Onodera, H. | Effects of snow avalanche disturbance on regeneration of subalpine Abies mariesii forest, Northern Japan | No | Excluded at title/ abstract screening | |
| 461 | 2017 | Kalsnes, B.; Nadim, F.; Hermanns, R. L.; Hygen, H. O.; Petkovic, G.; Dolva, B. K.; Berg, H.; HØgvold, D. O. | Landslide risk management in Norway | No | Excluded at title/ abstract screening | |
| 462 | 2007 | Kariya, Y.; Sato, G.; Mokudai, K.; Komori, J.; Ishii, M.; Nishii, R.; Miyazawa, Y.; Tsumura, N. | Rockfall hazard in the Daisekkei Valley, the northern Japanese Alps, on 11 August 2005 | No | Irrelevant title/ abstract (reports from other methods) | |
| 463 | 2009 | Karsli, F.; Atasoy, M.; Yalcin, A.; Reis, S.; Demir, O.; Gokceoglu, C. | Effects of land-use changes on landslides in a landslide-prone area (Ardesen, Rize, NE Turkey) | No | Excluded at title/ abstract screening | |
| 464 | 2006 | Kaufmann, Marc; Moser, Berthold; Lederer, Wolfgang | Changes in injury patterns and severity in a helicopter air-rescue system over a 6-year period | No | No relevant statistics obtained | |
| 465 | 2022 | Kazakova, E. N.; Podolskiy, E. A.; Kazakov, N. A. | Catastrophic avalanches on Sakhalin and the Kuril Islands (1910-2020) | No | No relevant statistics obtained | |
| 466 | 2015 | Kehoe A, Smith JE, Edwards A, et al. | The changing face of major trauma in the UK. | No | Irrelevant title/ abstract (reports from other methods) | |

**Appendix. Data screening record.**

**This table records inclusion and exclusion decisions (with reasons for exclusion) for 1031 studies/registries (750 unique ones from database search and 281 unique ones from reference screening and expert consultation); Reasons to exclude correspond to Fig 1 in the paper**

| Index | Publication Year | Author | Title | If included | Reason for exclusion | URL for grey included paper |
|---|---|---|---|---|---|---|
| 467 | 2004 | Kempainen RR, Brunette DD. | The evaluation and management of accidental hypothermia. | No | Irrelevant title/ abstract (reports from other methods) | |
| 468 | 2005 | Kent, H. | Snow man: Civil engineer Bruce Jamieson studies the threat of avalanches | No | Excluded at title/ abstract screening | |
| 469 | 2007 | Kent, Heather | AVALANCHE ASSESSOR | No | Excluded at title/ abstract screening | |
| 470 | 2001 | Kern M. T.F., and Schweizer J. | Feldversuch zur Wirksamkeit einiger neuer Lawinen-Rettungsgeräte. | No | Irrelevant title/ abstract (reports from other methods) | |
| 471 | 2001 | Keylock, Christopher J.; Barbolini, Massimiliano | Snow avalanche impact pressure - vulnerability relations for use in risk assessment | No | No relevant statistics obtained | |
| 472 | 2004 | Khanizadeh, S.; Rekika, D.; Levasseur, A.; Groleau, Y.; Richer, C.; Fisher, H. | Growing grapes in a cold climate with winter temperature below-25 degrees C | No | Excluded at title/ abstract screening | |
| 473 | 1994 | Kizer, K. W.; Macquarrie, M. B.; Kuhn, B. J.; Scannell, P. D. | DEEP SNOW IMMERSION DEATHS - A SNOWBOARDING DANGER | No | No relevant statistics obtained | |
| 474 | 2004 | Knight B, Saukko PJ. | Knight's forensic pathology | No | Irrelevant title/ abstract (reports from other methods) | |
| 475 | 2016 | Kobek, M.; Skowronek, R.; Jabłoński, C.; Jankowski, Z.; Pałasz, A. | Histopathological changes in lungs of the mountain snow avalanche victims and its potential usefulness in determination of cause and mechanism of death | Yes, only for systematic review | NA, since included | |
| 476 | 2007 | Koçyigit, Önder; Gürer, Ibrahim | Effect of the Voellmy Coefficients on Determining Run-out Distance: A Case Study at Uzungöl, Turkey | No | Irrelevant title/ abstract (reports from other methods) | |
| 477 | 2010 | Kodas, Michael | Avalanche Survival: Know Your Snow | No | Irrelevant title/ abstract (reports from other methods) | |
| 478 | 1998 | Koerner, Brendan I. | A mountaineer's final summit | No | Irrelevant title/ abstract (reports from other methods) | |

| **Appendix. Data screening record.** | | | | | | |
|---|---|---|---|---|---|---|
| **This table records inclusion and exclusion decisions (with reasons for exclusion) for 1031 studies/registries (750 unique ones from database search and 281 unique ones from reference screening and expert consultation); Reasons to exclude correspond to Fig 1 in the paper** | | | | | | |
| **Index** | **Publication Year** | **Author** | **Title** | **If included** | **Reason for exclusion** | **URL for grey included paper** |
| 479 | 2011 | Koike S, Ogawa T, Tanabe S, et al. | Collapse-to-emergency medical service cardiopulmonary resuscitation interval and outcomes of outof-hospital cardiopulmonary arrest: a nationwide observational study. | No | Irrelevant title/ abstract (reports from other methods) | |
| 480 | 2016 | Kornhall, Daniel K.; Logan, Spencer; Dolven, Thomas | Body Positioning of Buried Avalanche Victims | No | No relevant statistics obtained | |
| 481 | 2016 | Kornhall, Daniel K.; Martens-Nielsen, Julie | The prehospital management of avalanche victims | No | Excluded at title/ abstract screening | |
| 482 | 2015 | Kortiev, L. I.; Kortiev, A. L.; Tskhovrebov, I. P.; Margiev, E. A. | Theory and practice of anti-avalanche protection of communication lines and areas in mountainous regions | No | Excluded at title/ abstract screening | |
| 483 | 2013 | Kosiński, Sylweriusz; Jasiński, Jakub; Krzeptowski-Sabała, Stanisław; Gąsienica-Roj, Jan, Jr.; Górka, Andrzej | Deep snow immersion suffocation--the deadly threat | No | Excluded at title/ abstract screening | |
| 484 | 2014 | Kottmann, A.; Blancher, M.; Spichiger, T.; Boyd, J.; Brugger, H. | Development of a checklist for prehospital management of avalanche victims | No | No relevant statistics obtained | |
| 485 | 2018 | Kottmann, Alexandre; Carron, Pierre-Nicolas; Theiler, Lorenz; Albrecht, Roland; Tissi, Mario; Pasquier, Mathieu | Identification of the technical and medical requirements for HEMS avalanche rescue missions through a 15-year retrospective analysis in a HEMS in Switzerland: a necessary step for quality improvement | No | No relevant data (full text examined, papers through other methods) | |
| 486 | 2021 | Kottmann, Alexandre; Strapazzon, Giacomo; Pasquier, Mathieu; Blancher, Marc; Brugger, Hermann | Reply to letter: Adaptation to the 2017 ICAR MEDCOM Avalanche Victim Resuscitation Checklist | No | Excluded at title/ abstract screening | |
| 487 | 2008 | Kraas, F. | Megacities as global risk areas | No | Irrelevant title/ abstract (reports from other methods) | |
| 488 | 2012 | Kristensen, Ida; Kristensen, Krister | Human Error Revisited | No | Excluded at title/ abstract screening | |
| 489 | 1998 | Kristensen, K. | A survey of snow avalanche accidents in Norway | No | No relevant statistics obtained | |

| **Appendix. Data screening record.** | | | | | | |
|---|---|---|---|---|---|---|
| **This table records inclusion and exclusion decisions (with reasons for exclusion) for 1031 studies/registries (750 unique ones from database search and 281 unique ones from reference screening and expert consultation); Reasons to exclude correspond to Fig 1 in the paper** | | | | | | |
| **Index** | **Publication Year** | **Author** | **Title** | **If included** | **Reason for exclusion** | **URL for grey included paper** |
| **490** | **2016** | Kulakowski, D.; Barbeito, I.; Casteller, A.; Kaczka, R. J.; Bebi, P. | NOT ONLY CLIMATE: INTERACTING DRIVERS OF TREELINE CHANGE IN EUROPE | No | Excluded at title/ abstract screening | |
| **491** | **2016** | Kumar, S.; Snehmani; Srivastava, P. K.; Gore, A.; Singh, M. K. | Fuzzy-frequency ratio model for avalanche susceptibility mapping | No | Excluded at title/ abstract screening | |
| **492** | **2017** | Kumar, S.; Srivastava, P. K.; Snehmani | GIS-based MCDA–AHP modelling for avalanche susceptibility mapping of Nubra valley region, Indian Himalaya | No | Excluded at title/ abstract screening | |
| **493** | **1973** | L. J. Lugger | Obduktionsergebnisse bei Lawinenverunfallten | Yes, for meta-analysis and systematic review | NA, since included | |
| **494** | **1977** | Laatsch, W. | The origin of avalanche tracks in high mountain forests | No | Irrelevant title/ abstract (reports from other methods) | |
| **495** | **2009** | Lambrose, Pat | Using Geospatial Tools for Mapping Utah Avalanches | No | Irrelevant title/ abstract (reports from other methods) | |
| **496** | **2020** | Landrø, Markus; Hetland, Audun; Engeset, Rune Verpe; Pfuhl, Gerit | Avalanche decision-making frameworks: Factors and methods used by experts | No | Excluded at title/ abstract screening | |
| **497** | **1980** | Lapras | Pathologie des ensevelis | Yes, only for systematic review | NA, since included | |
| **498** | **2014** | Largent, Emily | Is it ethical to hire sherpas when climbing Mount Everest? | No | Excluded at title/ abstract screening | |
| **499** | **1999** | Larkin, Leah | White Death in the Alps | No | No relevant statistics obtained | |
| **500** | **1999** | Laskowski-Jones, L. | Responding to winter emergencies | No | Excluded at title/ abstract screening | |
| **501** | **2014** | Laura, Maguire | The Human Behind The Factor: A Brief Look at How Context Informs Practice in Recreational Backcountry Users | No | Excluded at title/ abstract screening | |
| **502** | **1978** | LaValla, Patrick; Emergency Response Inst, Tacoma W. A.; Survival Education Association, Tacoma W. A. | Resource Guide for Search and Rescue Training Materials | No | Irrelevant title/ abstract (reports from other methods) | |
| **503** | **2006** | Lazar, Brian; Williams, Mark | Climate Change in Western Ski Areas: Timing of Wet Avalanches in Aspen Ski Area in the Years 2030 and 2100 | No | Excluded at title/ abstract screening | |

**Appendix. Data screening record.**

**This table records inclusion and exclusion decisions (with reasons for exclusion) for 1031 studies/registries (750 unique ones from database search and 281 unique ones from reference screening and expert consultation); Reasons to exclude correspond to Fig 1 in the paper**

| Index | Publication Year | Author | Title | If included | Reason for exclusion | URL for grey included paper |
|---|---|---|---|---|---|---|
| 504 | 2005 | Legriel S, Bouyon A, Nekhili N, et al | Therapeutic hypothermia for coma after cardiorespiratory arrest caused by hanging. | No | Irrelevant title/ abstract (reports from other methods) | |
| 505 | 2021 | Lei, Y. B.; Yao, T. D.; Tian, L. D.; Sheng, Y. W.; Liao, J. J.; Zhao, H. B.; Yang, W.; Yang, K.; Berthier, E.; Brun, F.; Gao, Y.; Zhu, M. L.; Wu, G. J. | Response of downstream lakes to Aru glacier collapses on the western Tibetan Plateau | No | Excluded at title/ abstract screening | |
| 506 | 2011 | Leiter, Andrea M. | The sense of snow – Individuals' perception of fatal avalanche events | No | Excluded at title/ abstract screening | |
| 507 | 2014 | Leiter, Andrea M.; Pruckner, Gerald J. | Timing effects in health valuations | No | Irrelevant title/ abstract (reports from other methods) | |
| 508 | 2013 | Leonardi, A.; Wittel, F. K.; Mendoza, M.; Herrmann, H. J. | Multiphase debris flow simulations with the discrete element method coupled with a lattice-boltzmann fluid | No | Irrelevant title/ abstract (reports from other methods) | |
| 509 | 2013 | Li, W. L.; Huang, R. Q.; Tang, C.; Xu, Q.; van Westen, C. | Co-seismic landslide inventory and susceptibility mapping in the 2008 Wenchuan earthquake disaster area, China | No | Excluded at title/ abstract screening | |
| 510 | 2020 | Li, Xingyue; Sovilla, Betty; Jiang, Chenfanfu; Gaume, Johan | The mechanical origin of snow avalanche dynamics and flow regime transitions | No | Excluded at title/ abstract screening | |
| 511 | 2021 | Li, Xingyue; Sovilla, Betty; Jiang, Chenfanfu; Gaume, Johan | Three-dimensional and real-scale modeling of flow regimes in dense snow avalanches | No | Irrelevant title/ abstract (reports from other methods) | |
| 512 | 2012 | Lipsitz, George | In an Avalanche Every Snowflake Pleads Not Guilty": The Collateral Consequences of Mass Incarceration and Impediments to Women's Fair Housing Rights | No | Excluded at title/ abstract screening | |
| 513 | 2012 | Lischke, V.; Berner, A.; Kopp, K. H.; Mann, A. | Mountain rescue services by the German Red Cross Organization and technical rescue principles | No | No relevant statistics obtained | |
| 514 | 2014 | Lischke, V.; Berner, A.; Pietsch, U.; Mann, A. | Cardiac arrest in mountain areas during winter season: Different causes with different outcomes | No | No relevant statistics obtained | |

| Appendix. Data screening record. | | | | | | |
|---|---|---|---|---|---|---|
| This table records inclusion and exclusion decisions (with reasons for exclusion) for 1031 studies/registries (750 unique ones from database search and 281 unique ones from reference screening and expert consultation); Reasons to exclude correspond to Fig 1 in the paper | | | | | | |
| **Index** | **Publication Year** | **Author** | **Title** | **If included** | **Reason for exclusion** | **URL for grey included paper** |
| 515 | 2020 | Lischke, V.; Berner, A.; Schiffer, J.; Muller, U.; Dehne, M.; Pietsch, U. | Mountain Rescue Service in Germany | No | No relevant statistics obtained | |
| 516 | 2001 | Lischke, V.; Byhahn, C.; Halbig, S.; Kessler, P. | Emergency management of avalanche accidents | No | No relevant statistics obtained | |
| 517 | 2002 | Lischke, V.; Byhahn, C.; Halbig, S.; Westphal, K.; Mann, A.; Kessler, P. | Helicopter supported emergency medicine in mountain areas | No | No relevant statistics obtained | |
| 518 | 2001 | Lischke, V.; Byhahn, C.; Halbig, S.; Westphal, K.; Wirth, S. O.; Mann, A.; Kessler, P. | Special challenge for emergency physicians in alpine areas | No | No relevant statistics obtained | |
| 519 | | Lischner, B. | Lifeless avalanche victims probably beyond saving | No | Irrelevant title/ abstract (reports from other methods) | |
| 520 | 2015 | Liu, G. J.; Lv, C. Y.; Zhang, X. M.; Wei, J.; Lu, Y. | EFFECT OF WATER SUPPLY AND SOWING DEPTH ON SEEDLING EMERGENCE IN TWO HALOXYLON SPECIES IN THE JUNGAR BASIN | No | Irrelevant title/ abstract (reports from other methods) | |
| 521 | 2018 | Liu, W.; He, S. M. | Dynamic simulation of a mountain disaster chain: landslides, barrier lakes, and outburst floods | No | Excluded at title/ abstract screening | |
| 522 | 1999 | Lloyd, Jillian | Winter playground holds pitfalls for unwary | No | Excluded at title/ abstract screening | |
| 523 | 1996 | Locher, T.; Walpoth, B. H. | Differential diagnosis of circulatory failure in hypothermic avalanche victims: retrospective analysis of 32 avalanche accidents | Yes, only for systematic review | NA, since included | |
| 524 | 2014 | Logan, Spencer; Greene, Ethan | The Distribution of Fatalities by Avalanche Problem in Colorado, 1998-99 to 2012-13 | No | Excluded at title/ abstract screening | |
| 525 | 2014 | Lombardo, L.; Cama, M.; Maerker, M.; Rotigliano, E. | A test of transferability for landslides susceptibility models under extreme climatic events: Application to the Messina 2009 disaster | No | Excluded at title/ abstract screening | |
| 526 | 2003 | Lomoschitz FM, Eisenhuber E, Linnau KF, Peloschek P, Schoder M, Bankier A | Imaging of chest trauma: radiological patterns of injury and diagnostic algorithms | No | Irrelevant title/ abstract (reports from other methods) | |

| Appendix. Data screening record. | | | | | | |
|---|---|---|---|---|---|---|
| **This table records inclusion and exclusion decisions (with reasons for exclusion) for 1031 studies/registries (750 unique ones from database search and 281 unique ones from reference screening and expert consultation); Reasons to exclude correspond to Fig 1 in the paper** | | | | | | |
| **Index** | **Publication Year** | **Author** | **Title** | **If included** | **Reason for exclusion** | **URL for grey included paper** |
| **527** | **2020** | López-Moreno, J. I.; Navarro, F.; Izagirre, E.; Alonso, E.; Rico, I.; Zabalza, J.; Revuelto, J. | Glacier and climate evolution in the pariacacÁ mountains, peru | No | Excluded at title/ abstract screening | |
| **528** | **2012** | Lovejoy, David W. | Avalanche Education in the United States: The Good, the Bad, and the Ugly | No | Excluded at title/ abstract screening | |
| **529** | **2014** | Lundberg, A. | Skredmjelt oxytropis campestris ssp. Scotica i noreg. Utbreiing, økologi og tilstand | No | Excluded at title/ abstract screening | |
| **530** | **2013** | Lunde, Albert; Kristensen, Krister | Avalanche rescue and mission risk in Norway 1996-2010 | No | Irrelevant title/ abstract (reports from other methods) | |
| **531** | **2019** | Lunde, Albert; Njå, Ove | Rescue performance in Norwegian road related avalanche incidents | No | Excluded at title/ abstract screening | |
| **532** | **2019** | Lunde, Albert; Tellefsen, Christen | Patient and rescuer safety: recommendations for dispatch and prioritization of rescue resources based on a retrospective study of Norwegian avalanche incidents 1996-2017 | No | Excluded at title/ abstract screening | |
| **533** | **2020** | Lupiano, V.; Chidichimo, F.; Machado, G.; Catelan, P.; Molina, L.; Calidonna, C. R.; Straface, S.; Crisci, G. M.; Di Gregorio, S. | From examination of natural events to a proposal for risk mitigation of lahars by a cellular-automata methodology: a case study for Vascim valley, Ecuador | No | Excluded at title/ abstract screening | |
| **534** | **2004** | Lutz, Eric R; Birkeland, Karl W; Kronholm, Kalle; Hansen, Kathy; Aspinall, Richard | CORRELATING SNOW MICRO-STRUCTURE WITH SNOW SHEAR STRENGTH | No | Irrelevant title/ abstract (reports from other methods) | |
| **535** | **2016** | Luzon, P. K.; Montalbo, K.; Galang, J.; Sabado, J. M.; Escape, C. M.; Felix, R.; Lagmay, A. M. F. | Hazard mapping related to structurally controlled landslides in Southern Leyte, Philippines | No | Excluded at title/ abstract screening | |
| **536** | **1999** | Lynch, T. A. | NARSID -- what is it? Non avalanche related snow immersion death | No | Excluded at title/ abstract screening | |
| **537** | **2014** | M, L. | WHAT WERE THEY THINKING? | No | Excluded at title/ abstract screening | |

**Appendix. Data screening record.**

**This table records inclusion and exclusion decisions (with reasons for exclusion) for 1031 studies/registries (750 unique ones from database search and 281 unique ones from reference screening and expert consultation); Reasons to exclude correspond to Fig 1 in the paper**

| Index | Publication Year | Author | Title | If included | Reason for exclusion | URL for grey included paper |
|---|---|---|---|---|---|---|
| 538 | 1996 | Mace, Richard D.; Waller, John S.; Manley, Timothy L.; Lyon, L. Jack; Zuuring, Hans | Relationships among grizzly bears, roads and habitat in the Swan Mountains, Montana | No | Excluded at title/ abstract screening | |
| 539 | 1999 | MacKenzie, Debora | Cold comfort | No | Excluded at title/ abstract screening | |
| 540 | 2012 | Macqueen, K. E. N. | BREAKING OUT OF BOUNDS | No | Excluded at title/ abstract screening | |
| 541 | 2005 | Macqueen, Ken | DEATH RAINS DOWN | No | Excluded at title/ abstract screening | |
| 542 | 2014 | Mair P, Brugger H, Mair B, Moroder L, Ruttmann E | Is extracorporeal rewarming indicated in avalanche victims with unwitnessed hypothermic cardiorespiratory arrest? | No | Irrelevant title/ abstract (reports from other methods) | |
| 543 | 1998 | Mair P., Kornberger E., Schwarz B., Baubin M., and Hoermann C | Forward blood flow during cardiopulmonary resuscitation in patients with severe accidental hypothermia | No | Irrelevant title/ abstract (reports from other methods) | |
| 544 | 1997 | Mair, P.; Voelckel, W.; Baubin, M.; Mutz, N. | Intensive care implications of mountain injuries | No | Excluded at title/ abstract screening | |
| 545 | 2013 | Mair, Peter; Frimmel, Christian; Vergeiner, Gernot; Hohlrieder, Matthias; Moroder, Luca; Hoesl, Peter; Voelckel, Wolfgang | Emergency medical helicopter operations for avalanche accidents | Yes, only for systematic review | NA, since included | |
| 546 | 1995 | Malanson, G. P.; Cairns, D. M. | EFFECTS OF INCREASED CLOUD-COVER ON A MONTANE FOREST LANDSCAPE | No | Excluded at title/ abstract screening | |
| 547 | 2015 | Malta Hansen C, Kragholm K, Pearson DA, et al. | Association of bystander and first-responder intervention with survival after out-of-hospital cardiac arrest in North Carolina | No | Irrelevant title/ abstract (reports from other methods) | |
| 548 | 2014 | Maltby, Emily | High Risk | No | Excluded at title/ abstract screening | |
| 549 | 1976 | Mang, W. R.; Maurer, P. C. | The fatal ski accident | No | No relevant statistics obtained | |
| 550 | 2018 | Mannberg, A.; Hendrikx, J.; Landro, M.; Stefan, M. A. | Who's at risk in the backcountry? Effects of individual characteristics on hypothetical terrain choices | No | No relevant statistics obtained | |
| 551 | 2021 | Mannberg, Andrea; Hendrikx, Jordy; Johnson, Jerry; Hetland, Audun | Powder Fever and Its Impact on Decision-Making in Avalanche Terrain | No | Excluded at title/ abstract screening | |

| Appendix. Data screening record. | | | | | | |
|---|---|---|---|---|---|---|
| **This table records inclusion and exclusion decisions (with reasons for exclusion) for 1031 studies/registries (750 unique ones from database search and 281 unique ones from reference screening and expert consultation); Reasons to exclude correspond to Fig 1 in the paper** | | | | | | |
| **Index** | **Publication Year** | **Author** | **Title** | **If included** | **Reason for exclusion** | **URL for grey included paper** |
| 552 | 2017 | Marengo, Davide; Monaci, Maria Grazia; Miceli, Renato | Winter recreationists' self-reported likelihood of skiing backcountry slopes: Investigating the role of situational factors, personal experiences with avalanches and sensation-seeking | No | Excluded at title/ abstract screening | |
| 553 | 1999 | Margreth, S.; Funk, M. | Hazard mapping for ice and combined snow/ice avalanches - two case studies from the Swiss and Italian Alps | No | Excluded at title/ abstract screening | |
| 554 | 2016 | Maringer, Janet; Ascoli, Davide; Küffer, Nicolas; Schmidtlein, Sebastian; Conedera, Marco | What drives European beech (Fagus sylvatica L.) mortality after forest fires of varying severity? | No | Excluded at title/ abstract screening | |
| 555 | 2020 | Marino, P.; Comegna, L.; Damiano, E.; Olivares, L.; Greco, R. | Monitoring the Hydrological Balance of a Landslide-Prone Slope Covered by Pyroclastic Deposits over Limestone Fractured Bedrock | No | Excluded at title/ abstract screening | |
| 556 | 2009 | Maris, Malou N. A.; Giraud, GÃ©rald; Durand, Yves; Navarre, Jean-Pierre; MÃ©rindol, Laurent | Results of 50 Years of Climate Reanalysis in the French Pyrenees (1958-2008) Using the Safran and Crocus Models | No | Excluded at title/ abstract screening | |
| 557 | 2004 | Markels, Alex | Pushing the Envelope | No | Excluded at title/ abstract screening | |
| 558 | 2021 | Markusoff, Jason | SAVED FROM A SNOWY TOMB | No | Excluded at title/ abstract screening | |
| 559 | 1970 | Markwalder | Medizinische Aspekte bei Lawinenunf/illen | Yes, only for systematic review | NA, since included | |
| 560 | 1976 | Markwalder K | Die Pathophysiologie des Lawinenunfalles. | No | Data already covered | |
| 561 | 1998 | Marshall, Jane | Guide's errors may have led to deaths | No | Excluded at title/ abstract screening | |
| 562 | 2021 | Martha, T. R.; Roy, P.; Jain, N.; Kumar, K. V.; Reddy, P. S.; Nalini, J.; Sharma, Svsp; Shukla, A. K.; Rao, Khvd; Narender, B.; Rao, P. V. N.; Muralikrishnan, S. | Rock avalanche induced flash flood on 07 February 2021 in Uttarakhand, India- a photogeological reconstruction of the event | No | Irrelevant title/ abstract (reports from other methods) | |
| 563 | 1999 | Martinelli M, Jr.; Leaf, C. F. | Historic avalanches in the northern front range and the central and northern mountains of Colorado | No | Excluded at title/ abstract screening | |

| **Appendix. Data screening record.** | | | | | | |
|---|---|---|---|---|---|---|
| **This table records inclusion and exclusion decisions (with reasons for exclusion) for 1031 studies/registries (750 unique ones from database search and 281 unique ones from reference screening and expert consultation); Reasons to exclude correspond to Fig 1 in the paper** | | | | | | |
| **Index** | **Publication Year** | **Author** | **Title** | **If included** | **Reason for exclusion** | **URL for grey included paper** |
| **564** | **2022** | Martínez, Íñigo Soteras; Ayala, Marc; Casadesús, Josep Maria; Domènech, Glòria Martí; Trullàs, Joan Carles; Mariño, Robert Blasco | Main causes of accidental deaths due to avalanches in the Catalan Pyrenees: a review of 50 years | Yes, for meta-analysis and systematic review | NA, since included | |
| **565** | **2010** | Matthias, Hohlrieder; Franz, Kroesslhuber; Wolfgang, Voelckel; Martin, Lutz; Peter, Mair | Experience with Helicopter Rescue Missions for Crevasse Accidents | No | Excluded at title/ abstract screening | |
| **566** | **2008** | Matthias, Hohlrieder; Stephanie, Thaler; Walter, Wuertl; Wolfgang, Voelckel; Hanno, Ulmer; Hermann, Brugger; Peter, Mair | Rescue Missions for Totally Buried Avalanche Victims: Conclusions from 12 Years of Experience | No | No relevant statistics obtained | |
| **567** | **2011** | McCafferty, Keith | White Death | No | No relevant statistics obtained | |
| **568** | **2008** | McCammon I, Diitolla M, McIntosh S. | Terrain and traumatic injury in US avalanche accidents. | No | Irrelevant title/ abstract (reports from other methods) | |
| **569** | **2008** | McCammon I, Haegeli P | Out-of-bounds avalanche awareness: assessment, current practices and future management. | No | Irrelevant title/ abstract (reports from other methods) | |
| **570** | **2009** | McCammon, Ian | Human Factors in Avalanche Accidents: Evolution and Interventions | No | Excluded at title/ abstract screening | |
| **571** | **2007** | McCammon, Ian; Hägeli, Pascal | An evaluation of rule-based decision tools for travel in avalanche terrain | No | Excluded at title/ abstract screening | |
| **572** | **2005** | McCarthy, Terry | How to Survive an Avalanche | No | No relevant statistics obtained | |
| **573** | **2006** | McClung D, Schaerer PA | The avalanche handbook | No | All relevant statistics were cited elsewhere | |
| **574** | **2016** | McClung, D. M. | Avalanche character and fatalities in the high mountains of Asia | No | No relevant statistics obtained | |
| **575** | **2014** | McClung, D. M. | Risk analyses for dry snow slab avalanches released by skier triggering | No | No relevant statistics obtained | |
| **576** | **2011** | McClung, D. M. | The strength and weight of evidence in backcountry avalanche forecasting | No | No relevant statistics obtained | |
| **577** | **2012** | McClung, D. M. | Effects of El NiÃ±o and La NiÃ±a on Snow Avalanche Patterns | No | Excluded at title/ abstract screening | |

| **Appendix. Data screening record.** | | | | | | |
|---|---|---|---|---|---|---|
| **This table records inclusion and exclusion decisions (with reasons for exclusion) for 1031 studies/registries (750 unique ones from database search and 281 unique ones from reference screening and expert consultation); Reasons to exclude correspond to Fig 1 in the paper** | | | | | | |
| **Index** | **Publication Year** | **Author** | **Title** | **If included** | **Reason for exclusion** | **URL for grey included paper** |
| 578 | 2000 | McFadden, Robert D. | Rare Avalanche Kills One On an Adirondack Slope | No | Excluded at title/ abstract screening | |
| 579 | 2008 | McIntosh SE, Campbell AD, Dow J, et al. | Mountaineering fatalities on Denali | No | Irrelevant title/ abstract (reports from other methods) | |
| 580 | 2019 | McIntosh, S. E.; Brant-Zawadzki, G.; Milliner, B. H.; Christensen, E. D.; Nyberg, A. A.; Grissom, C. K.; Olivares, C. R.; Kim, H. S.; Tremper, B. | Cause of Death in Utah Avalanche Fatalities, 2006–2007 through 2017–2018 Winter Seasons | Yes, for meta-analysis and systematic review | NA, since included | |
| 581 | 2015 | McIntosh, S. E.; Crouch, A. K.; Dorais, A.; McDevitt, M.; Wilson, C.; Harmston, C. H.; Radwin, M. I.; Grissom, C. K. | Effect of Head and Face Insulation on Cooling Rate During Snow Burial | No | Excluded at title/ abstract screening | |
| 582 | 2020 | McIntosh, S. E.; Little, C. E.; Seibert, T. D.; Polukoff, N. E.; Grissom, C. K. | Avalanche airbag post-burial active deflation — The ability to create an air pocket to delay asphyxiation and prolong survival | No | Excluded at title/ abstract screening | |
| 583 | 2007 | McIntosh, Scott E.; Grissom, Colin K.; Olivares, Christopher R.; Kim, Han S.; Tremper, Bruce | Cause of death in avalanche fatalities | Yes, for meta-analysis and systematic review | NA, since included | |
| 584 | 1999 | McLean, Candis | Ignorance kills | No | Excluded at title/ abstract screening | |
| 585 | 2006 | McManus, Reed | Survive This! | No | Excluded at title/ abstract screening | |
| 586 | 2023 | Md, David C. Fiore; Bs, Andrew P. Cobourn; Bs, Spencer J. H. Trivitt; PhD, Jordy Hendrikx; Da, Jerry D. Johnson; Md, Esteban A. Valle | HOW COVID AFFECTED BACKCOUNTRY SKIING IN THE 2020 - 2021 SEASON | No | Excluded at title/ abstract screening | |
| 587 | 1985 | Meier, Joel | Risk and Hazard Management in High Adventure Outdoor Pursuits | No | No relevant statistics obtained | |
| 588 | 2012 | Meiners, Theo; Grant, Kim; Sehnert, Sam; Belford, Matt; Baker, Jessica; Maris, Robb; Meiners, Aidan; Fischer, Craig; Houfek, Nicholas; Pope, Josh; Sanders, A. J.; Trombetta, Mike; Janjigian, Dan; Green, Sam | Practitioners View on Quick Study of Snowpack: How to Explain the Vocabulary for Pole Probe Tests and Slope Cutting | No | Excluded at title/ abstract screening | |

| Appendix. Data screening record. | | | | | | |
|---|---|---|---|---|---|---|
| This table records inclusion and exclusion decisions (with reasons for exclusion) for 1031 studies/registries (750 unique ones from database search and 281 unique ones from reference screening and expert consultation); Reasons to exclude correspond to Fig 1 in the paper | | | | | | |
| **Index** | **Publication Year** | **Author** | **Title** | **If included** | **Reason for exclusion** | **URL for grey included paper** |
| **589** | **1989** | Meister, R. | Influence of strong winds on snow distribution and avalanche activity | No | Excluded at title/ abstract screening | |
| **590** | **2023** | Meloche, Francis; Guillet, Louis; Gauthier, Francis; Langlois, Alexandre; Gaume, Johan | INFLUENCE OF SLAB DEPTH SPATIAL VARIABILITY ON THE PROBABILITY AND RELEASE SIZE OF SKIER-TRIGGERED AVALANCHES | No | Excluded at title/ abstract screening | |
| **591** | **2016** | Meng, Q. K.; Miao, F.; Zhen, J.; Huang, Y.; Wang, X. Y.; Peng, Y. | Impact of earthquake-induced landslide on the habitat suitability of giant panda in Wolong, China | No | Excluded at title/ abstract screening | |
| **592** | **2020** | Mergili, M.; Jaboyedoff, M.; Pullarello, J.; Pudasaini, S. P. | Back calculation of the 2017 Piz Cengalo-Bondo landslide cascade with r.avaflow: what we can do and what we can learn | No | Excluded at title/ abstract screening | |
| **593** | **2020** | Mergili, M.; Pudasaini, S. P.; Emmer, A.; Fischer, J. T.; Cochachin, A.; Frey, H. | Reconstruction of the 1941 GLOF process chain at Lake Palcacocha (Cordillera Blanca, Peru) | No | Excluded at title/ abstract screening | |
| **594** | **2009** | Mersch, Jan; Kühberger, Anton | Intuition, recognition and patterns – decision-making by mountain guides in avalanche situations | No | Irrelevant title/ abstract (reports from other methods) | |
| **595** | **2005** | Metternicht, Graciela; Hurni, Lorenz; Gogu, Radu | Remote sensing of landslides: An analysis of the potential contribution to geo-spatial systems for hazard assessment in mountainous environments | No | Excluded at title/ abstract screening | |
| **596** | **2022** | Michaelsen, Bjørn; Stewart-Patterson, Iain; Rolland, Carsten G.; Hetland, Audun; Engeset, Rune V. | Behavior in Avalanche Terrain: An Exploratory Study of Illegal Snowmobiling in Norway | No | Excluded at title/ abstract screening | |
| **597** | **2018** | Miller, Aubrey D.; Squires, John R.; Olson, Lucretia E.; Roberts, Elizabeth K. | Terrain selection and forecasted avalanche danger: Do recreationists select safer terrain when the forecasted danger increases? | No | Excluded at title/ abstract screening | |
| **598** | **2014** | Miller, Daniel A.; Binger, Josephine | Experimentally Derived Affected Snow Volumes and Dynamic Response of Hard Slab Snow From Explosives | No | Excluded at title/ abstract screening | |

| Appendix. Data screening record. | | | | | | |
|---|---|---|---|---|---|---|
| **This table records inclusion and exclusion decisions (with reasons for exclusion) for 1031 studies/registries (750 unique ones from database search and 281 unique ones from reference screening and expert consultation); Reasons to exclude correspond to Fig 1 in the paper** | | | | | | |
| **Index** | **Publication Year** | **Author** | **Title** | **If included** | **Reason for exclusion** | **URL for grey included paper** |
| **599** | **1984** | Minnich, R. A. | Snow drifting and timberline dynamics on Mount San Gorgonio, California, USA | No | Excluded at title/ abstract screening | |
| **600** | **2020** | Mitchell, A.; McDougall, S.; Nolde, N.; Brideau, M. A.; Whittall, J.; Aaron, J. B. | Rock avalanche runout prediction using stochastic analysis of a regional dataset | No | Irrelevant title/ abstract (reports from other methods) | |
| **601** | **1967** | Mithoefer JC, Mead G, Hughes JMB, Iliff LD, Campbell EJM | A method of distinguishingdeath due to cardiac arrest from asphyxia | No | Irrelevant title/ abstract (reports from other methods) | |
| **602** | **2021** | Mittermair, Christof; Foidl, Eva; Wallner, Bernd; Brugger, Hermann; Paal, Peter | Extreme Cooling Rates in Avalanche Victims: Case Report and Narrative Review | No | Excluded at title/ abstract screening | |
| **603** | **2021** | Mkhwanazi, E. | Does Covid-19 Rupture Theodicy? Theophilosophical Musings | No | Excluded at title/ abstract screening | |
| **604** | **1995** | Mock, C. J. | Avalanche climatology of the continental zone in the southern rocky mountains | No | No relevant statistics obtained | |
| **605** | **2004** | Modroo, J. J.; Olhoeft, G. R. | Avalanche rescue using Ground penetrating radar | No | No relevant statistics obtained | |
| **606** | **2016** | Möhle, S.; Beierle, C. | Supporting the forecast of snow avalanches in the canton of glarus in eastern switzerland: A case study | No | Excluded at title/ abstract screening | |
| **607** | **2014** | Möhle, S.; Bründl, M.; Beierle, C. | Modeling a system for decision support in snow avalanche warning using balanced random forest and weighted random forest | No | Excluded at title/ abstract screening | |
| **608** | **2010** | Molinski, Dan; Pokharel, Krishna | WORLD WATCH | No | Excluded at title/ abstract screening | |
| **609** | **2013** | Moner, Ivan; OrguÃ©, Sara; GavaldÃ, Jordi; Bacardit, Montse | How Big is Big: Results of the Avalanche Size Classification Survey | No | Excluded at title/ abstract screening | |
| **610** | **2020** | Moore, G. W. K.; Cristofanelli, P.; Bonasoni, P.; Verza, G. P.; Semple, J. L. | Was an Avalanche Swarm Responsible for the Devastation at Mount Everest Base Camp During the April 2015 Nepal Earthquake? | No | No relevant statistics obtained | |
| **611** | **2017** | Moore, G. W. K.; Cristofanelli, P.; Bonasoni, P.; Verza, G. P.; Semple, J. L. | Automatic weather station observations of the April 2014 Mount Everest avalanche | No | Excluded at title/ abstract screening | |

**Appendix. Data screening record.**

**This table records inclusion and exclusion decisions (with reasons for exclusion) for 1031 studies/registries (750 unique ones from database search and 281 unique ones from reference screening and expert consultation); Reasons to exclude correspond to Fig 1 in the paper**

| Index | Publication Year | Author | Title | If included | Reason for exclusion | URL for grey included paper |
|---|---|---|---|---|---|---|
| 612 | 2018 | Moore, K.; Semple, J.; Cristofanelli, P.; Bonasoni, P.; Verza, P. G. | Analysis of the avalanche at mount everest base camp during the april 2015 earthquake in Nepal | No | No relevant statistics obtained | |
| 613 | 2008 | Moore, Mark | Enso and Avalanche Fatalities: Is there a Correlation? | No | Excluded at title/ abstract screening | |
| 614 | 2012 | Morath, Eric | U.S. WATCH | No | Excluded at title/ abstract screening | |
| 615 | 2015 | Moroder, Luca; Mair, Birgit; Brugger, Hermann; Voelckel, Wolfgang; Mair, Peter | Outcome of avalanche victims with out-of-hospital cardiac arrest | Yes, for meta-analysis and systematic review | NA, since included | |
| 616 | 2000 | Morris, Holly | Snowball From Hell | No | No relevant statistics obtained | |
| 617 | 2002 | Morton, M. L. | The Mountain White-crowned Sparrow: Migration and reproduction at high altitude | No | Excluded at title/ abstract screening | |
| 618 | 2019 | Moser, J. | Strategies and tactics of economic survival: De-Industrialisation, work, and change in an alpine mining community | No | Excluded at title/ abstract screening | |
| 619 | 2019 | Mott, Rebecca; Wolf, Andreas; Kehl, Maximilian; Kunstmann, Harald; Warscher, Michael; Grünewald, Thomas | Avalanches and micrometeorology driving mass and energy balance of the lowest perennial ice field of the Alps: a case study | No | Excluded at title/ abstract screening | |
| 620 | 2020 | Mughal, Jalaluddin; Abi-Habib, Maria; Masood, Salman | Avalanches Ravage Towns, Burying Dozens in Kashmir | No | No relevant statistics obtained | |
| 621 | 1998 | Murr, Andrew; Alexander, Karen | `The trail just gave out.' | No | Excluded at title/ abstract screening | |
| 622 | 2012 | Mutrie, T. I. M. | Skiers 'Who Know Better' Increasingly Fall Victim to Avalanches | No | Excluded at title/ abstract screening | |
| 623 | 2001 | Myerburg RJ. | Sudden cardiac death: exploring the limits of our knowledge | No | Irrelevant title/ abstract (reports from other methods) | |
| 624 | 2019 | Nakamura, K. | Implementation and demonstration of a system for the forecasting of surface avalanche potential caused by snowfall from a cyclone | No | Excluded at title/ abstract screening | |
| 625 | 2006 | Nansen, Eric | THE WELL-OUTFITTED BACKCOUNTRY SKIER | No | Excluded at title/ abstract screening | |
| 626 | 1993 | Nash, J. Madeleine; Blackman, Joni H. | Eluding the white death | No | No relevant statistics obtained | |

## Appendix. Data screening record.

**This table records inclusion and exclusion decisions (with reasons for exclusion) for 1031 studies/registries (750 unique ones from database search and 281 unique ones from reference screening and expert consultation); Reasons to exclude correspond to Fig 1 in the paper**

| Index | Publication Year | Author | Title | If included | Reason for exclusion | URL for grey included paper |
|---|---|---|---|---|---|---|
| 627 | 1975 | Neureuther G. | Avalanches: Protection, Location, Rescue. | No | Irrelevant title/ abstract (reports from other methods) | |
| 628 | 2014 | Ney, L.; Werner, D.; Bayer, A.; Mair, P. | Abortion of alpine rescue missions: Results of a preliminary survey | No | No relevant statistics obtained | |
| 629 | 2015 | Ng, Pearlly; Smith, William R.; Wheeler, Albert; McIntosh, Scott E. | Advanced Avalanche Safety Equipment of Backcountry Users: Current Trends and Perceptions | No | No relevant statistics obtained | |
| 630 | 2018 | Nichols, Ty B.; Hawley, Alana C.; Smith, William R.; Wheeler, Albert R.; McIntosh, Scott E.; Wheeler, Albert R., 3rd | Avalanche Safety Practices Among Backcountry Skiers and Snowboarders in Jackson Hole in 2016 | No | No relevant statistics obtained | |
| 631 | 2019 | Niedermeier, Martin; Gatterer, Hannes; Pocecco, Elena; Frühauf, Anika; Faulhaber, Martin; Menz, Verena; Burtscher, Johannes; Posch, Markus; Ruedl, Gerhard; Burtscher, Martin | Mortality in Different Mountain Sports Activities Primarily Practiced in the Winter Season-A Narrative Review | No | No relevant statistics obtained | |
| 632 | 2015 | Niederseer, D.; Schmied, C.; Niebauer, J. | Cardiovascular risk-benefit ratio of alpine skiing in recreational skiers | No | No relevant statistics obtained | |
| 633 | 2022 | Niemann, Danielle; Paul, Subrata; Rahman, Humairat H. | Avalanche Preparedness and Accident Analysis Among Backcountry Skier, Sidecountry, and Snowmobile Fatalities in the United States: 2009 to 2019 | No | Excluded at title/ abstract screening | |
| 634 | 2021 | Nikolaeva, S. A; Savchuk, D. A | Assessment of Dendrogeomorphological Dating Methods of Past Geomorphic Processes in the Aktru Headwater (the Russian Altai Mountains) | No | Excluded at title/ abstract screening | |
| 635 | 2010 | Nikolskiy, P. A.; Basilyan, A. E.; Sulerzhitsky, L. D.; Pitulko, V. V. | Prelude to the extinction: Revision of the Achchagyi-Allaikha and Berelyokh mass accumulations of mammoth | No | Excluded at title/ abstract screening | |

| Appendix. Data screening record. | | | | | | |
|---|---|---|---|---|---|---|
| **This table records inclusion and exclusion decisions (with reasons for exclusion) for 1031 studies/registries (750 unique ones from database search and 281 unique ones from reference screening and expert consultation); Reasons to exclude correspond to Fig 1 in the paper** | | | | | | |
| **Index** | **Publication Year** | **Author** | **Title** | **If included** | **Reason for exclusion** | **URL for grey included paper** |
| 636 | 2006 | Nixdorf-Miller A, Hunsaker DM, Hunsaker JC | Hypothermia and hyperthermia medicolegal investigation of morbidity and mortality from exposure to environmental temperature extremes | No | Irrelevant title/ abstract (reports from other methods) | |
| 637 | 2015 | Nolasco-Javier, D.; Kumar, L.; Tengonciang, A. M. P. | Rapid appraisal of rainfall threshold and selected landslides in Baguio, Philippines | No | Excluded at title/ abstract screening | |
| 638 | 2010 | Nordland, Rod; Wafa, Abdul Waheed | More Bodies Found After Afghan Avalanches; Death Toll Exceeds 160 | No | No relevant statistics obtained | |
| 639 | 2010 | Nordland, Rod; Wafa, Abdul Waheed | Avalanches Kill Dozens on Mountain Highway in Afghanistan | No | No relevant statistics obtained | |
| 640 | 2022 | Norheim, A. J.; Rannestad, B.; Howes, R.; Rein, E. B.; Jorum, E.; Friedl, K.; Havenith, G.; Teien, H. K.; Mercer, J. B.; Melau, J.; de Weerd, L.; Smith, M.; Taylor, N.; Voie, O. A.; Bergan-Skar, P.; Andrews, S.; Naesheim, T.; Steinberg, T. | Abstracts from The Cold Weather Operations Conference 2021 | No | Excluded at title/ abstract screening | |
| 641 | 1999 | North, Mark | LIfe Vest | No | Excluded at title/ abstract screening | |
| 642 | 2013 | Nuome Y, Bando K, Nagashima N, Takamura N. | Chuo University Bulletin of the Institute of Health and Sports Science | No | Irrelevant title/ abstract (reports from other methods) | |
| 643 | 1996 | O'Brien, Richard; Lidz, Franz | Lopsang's last climb | No | Excluded at title/ abstract screening | |
| 644 | 1972 | O'Loughlin, Colin Lockhart | Investigation of the stability of the steepland forest soils in the coast mountains, southwest British Columbia | No | Excluded at title/ abstract screening | |
| 645 | 2008 | Oberhammer, Rosmarie; Beikircher, Werner; Hörmann, Christoph; Lorenz, Ingo; Pycha, Roger; Adler-Kastner, Liselotte; Brugger, Hermann | Full recovery of an avalanche victim with profound hypothermia and prolonged cardiac arrest treated by extracorporeal re-warming | No | Excluded at title/ abstract screening | |
| 646 | 2020 | Ochtyra, A. | Forest Disturbances in Polish Tatra Mountains for 1985-2016 in Relation to Topography, Stand Features, and Protection Zone | No | Excluded at title/ abstract screening | |

**Appendix. Data screening record.**

**This table records inclusion and exclusion decisions (with reasons for exclusion) for 1031 studies/registries (750 unique ones from database search and 281 unique ones from reference screening and expert consultation); Reasons to exclude correspond to Fig 1 in the paper**

| Index | Publication Year | Author | Title | If included | Reason for exclusion | URL for grey included paper |
|---|---|---|---|---|---|---|
| 647 | 1957 | Ogura, R. | Studies On the Deposition of Calcium Salt | No | Excluded at title/ abstract screening | |
| 648 | 1980 | Okusa, S.; Anma, S. | Slope failures and tailings dam damage in the 1978 Izu-Ohshima-Kinkai earthquake | No | Excluded at title/ abstract screening | |
| 649 | 2006 | Olhoeft, G. R.; Modroo, J. J. | Locating and identifying avalanche victims with GPR | No | Excluded at title/ abstract screening | |
| 650 | 2003 | Oliver, Peter; Hewitt, Ben | Underestimating the Beast | No | Excluded at title/ abstract screening | |
| 651 | 1988 | Oliver, Valerie Smith; Sumner, Jim; Anchorage School District, A. K. | Natural Science of Alaska Handbook. Revised. Anchorage School District Elementary Science Program | No | Excluded at title/ abstract screening | |
| 652 | 2012 | Olmedo, R.; Olmedo, A.; Rico, J.; Sancho, J.; Rueda, C.; Perez, J.; Fernandez, A.; Ares, F.; Aguilera, D.; de Miguel, P.; Cerezo, C.; Bataller, V.; Villarroel, J. L.; Ieee | SICRA: a GNSS Cooperative System for Avalanche Rescue | No | Irrelevant title/ abstract (reports from other methods) | |
| 653 | 2018 | Olson, E. J.; Townsend, C. G. | An ArcGIS Model for Avalanche Risk Assessment in the North Cascades, Washington | No | Excluded at title/ abstract screening | |
| 654 | 2020 | osch M, Schranz A, Lener M, Burtscher M, Ruedl G | Incidences of fatalities on Austrian ski slopes: a 10-year analysis | No | Irrelevant title/ abstract (reports from other methods) | |
| 655 | 2015 | Oshima, Toru; Yoshikawa, Hiroshi; Ohtani, Maki; Mimasaka, Sohtaro | Three cases of suprachoroidal hemorrhage associated with chest compression or asphyxiation and detected using postmortem computed tomography | No | Excluded at title/ abstract screening | |
| 656 | 2015 | Oshiro K, Murakami T, Nishimura K. | Hokkaido Police's hypothermia wrapping method: The reducing effect of heat loss and successful cases in mountain rescues. | No | Irrelevant title/ abstract (reports from other methods) | |
| 657 | 2022 | Oshiro, K.; Tanioka, Y.; Schweizer, J.; Zafren, K.; Brugger, H.; Paal, P. | Prevention of Hypothermia in the Aftermath of Natural Disasters in Areas at Risk of Avalanches, Earthquakes, Tsunamis and Floods | No | Study a single cause of death | |

## Appendix. Data screening record.

**This table records inclusion and exclusion decisions (with reasons for exclusion) for 1031 studies/registries (750 unique ones from database search and 281 unique ones from reference screening and expert consultation); Reasons to exclude correspond to Fig 1 in the paper**

| Index | Publication Year | Author | Title | If included | Reason for exclusion | URL for grey included paper |
|---|---|---|---|---|---|---|
| 658 | 2022 | Oshiro, Kazue; Murakami, Tomikazu | Causes of death and characteristics of non-survivors rescued during recreational mountain activities in Japan between 2011 and 2015: a retrospective analysis | Yes, for meta-analysis and systematic review | NA, since included | |
| 659 | 2008 | Osterhuber, Randall | Avalanche Fatalities in California's Sierra Nevada, Winters 1987-2008 | No | Excluded at title/ abstract screening | |
| 660 | 2016 | Paal P, Gordon L, Strapazzon G, Brodmann Maeder M, Putzer G, Walpoth B, et al. | Accidental hypothermia an update: The content of this review is endorsed by the International Commission for Mountain Emergency Medicine (ICAR MEDCOM). | No | Irrelevant title/ abstract (reports from other methods) | |
| 661 | 2006 | Paal, P.; Beikircher, W.; Brugger, H. | Avalanche emergencies, Review of the current situation | No | No relevant statistics obtained | |
| 662 | 2010 | Paal, P.; Braun, P.; Ellmauer, P. P.; Schroeder, D.; Sumann, G.; Werner, A.; Wenzel, V.; Strapazzon, G.; Falk, M.; Brugger, H. | Factors affecting survival from avalanche burial-A pilot study | No | No relevant statistics obtained | |
| 663 | 2012 | Paal, Peter; Milani, Mario; Brown, Douglas; Boyd, Jeff; Ellerton, John | Termination of Cardiopulmonary Resuscitation in Mountain Rescue | No | Irrelevant title/ abstract (reports from other methods) | |
| 664 | 1999 | Page, C. E.; Atkins, D.; Shockley, L. W.; Yaron, M. | Avalanche deaths in the United States: a 45-year analysis | No | No relevant statistics obtained | |
| 665 | 2017 | Pakkanen T, Kämäräinen A, Huhtala H, et al. | Physician-staffed helicopter emergency medical service has a beneficial impact on the incidence of prehospital hypoxia and secured airways on patients with severe traumatic brain injury. | No | Irrelevant title/ abstract (reports from other methods) | |
| 666 | 2011 | Palumbo, A.; Hauler, F.; Dziunycz, P.; Schwager, K.; Soltermann, A.; Pretto, F.; Alonso, C.; Hofbauer, G. F.; Boyle, R. W.; Neri, D. | A chemically modified antibody mediates complete eradication of tumours by selective disruption of tumour blood vessels | No | Excluded at title/ abstract screening | |

## Appendix. Data screening record.

**This table records inclusion and exclusion decisions (with reasons for exclusion) for 1031 studies/registries (750 unique ones from database search and 281 unique ones from reference screening and expert consultation); Reasons to exclude correspond to Fig 1 in the paper**

| Index | Publication Year | Author | Title | If included | Reason for exclusion | URL for grey included paper |
|---|---|---|---|---|---|---|
| 667 | | Papola, Davide; Purgato, Marianna; Gastaldon, Chiara; Bovo, Chiara; van Ommeren, Mark; Barbui, Corrado; Tol, Wietse A. | Psychological and social interventions for the prevention of mental disorders in people living in low- and middle-income countries affected by humanitarian crises | No | Excluded at title/ abstract screening | |
| 668 | 2007 | Parson, Walther; Brandstätter, Anita; Niederstätter, Harald; Grubwieser, Petra; Scheithauer, Richard | Unravelling the mystery of Nanga Parbat | No | Irrelevant title/ abstract (reports from other methods) | |
| 669 | 2010 | Pascal Haegeli, Markus Falk, Hermann Brugger, Hans-JÃ¼rg Etter, Jeff Boyd | A Comparison of Avalanche Survival Patterns in Canada and Switzerland (ISSW) | No | Data already covered | |
| 670 | 2023 | Pasquier M, Strapazzon G, Kottmann A, et al. | On-site treatment of avalanche victims: a scoping review and 2023 recommendations of the international commission for mountain emergency medicine (ICAR MedCom | No | Irrelevant title/ abstract (reports from other methods) | |
| 671 | 2017 | Pasquier, Mathieu; Hugli, Olivier; Kottmann, Alexandre; Techel, Frank | Avalanche Accidents Causing Fatalities: Are They Any Different in the Summer? | Yes, only for systematic review | NA, since included | |
| 672 | 2020 | Patil, A.; Singh, G.; Kumar, S.; Mani, S.; Bandyopadhyay, D.; Nela, B. R.; Musthafa, M.; Mohanty, S. | Snow Characterization and Avalanche Detection in the Indian Himalaya | No | Excluded at title/ abstract screening | |
| 673 | 2020 | Peitzsch, E.; Boilen, S.; Logan, S.; Birkeland, K.; Greene, E. | Research note: How old are the people who die in avalanches? A look into the ages of avalanche victims in the United States (1950-2018) | No | Excluded at title/ abstract screening | |
| 674 | 2010 | Peitzsch, Erich H.; Hendrikx, Jordy; Fagre, Daniel B.; Reardon, Blase | Characterizing Wet Slab and Glide Slab Avalanche Occurrence Along the Going-to-the-Sun Road, Glacier National Park, Montana, USA | No | Excluded at title/ abstract screening | |
| 675 | 2004 | Pennington, Bill | Learning to Prevent Avalanche Deaths | No | Excluded at title/ abstract screening | |
| 676 | 2019 | Perrigo, Billy | 1 Skier Dead, 1 Critically Injured After Being Buried by Avalanche at New Mexico Ski Resort | No | Excluded at title/ abstract screening | |

**Appendix. Data screening record.**

**This table records inclusion and exclusion decisions (with reasons for exclusion) for 1031 studies/registries (750 unique ones from database search and 281 unique ones from reference screening and expert consultation); Reasons to exclude correspond to Fig 1 in the paper**

| Index | Publication Year | Author | Title | If included | Reason for exclusion | URL for grey included paper |
|---|---|---|---|---|---|---|
| 677 | 2013 | Perrod, MichaÃ«l Anthoine et Jean | Formation et Information des Pratiquants de la Raquette Ã Neige: Une DÃ©marche de la FFRandonnÃ©e | No | Excluded at title/ abstract screening | |
| 678 | 2006 | Peruzzi, Marc | ANATOMY OF A BURIAL | No | No relevant statistics obtained | |
| 679 | 2003 | Petrone, P.; Kuncir, E. J.; Asensio, J. A. | Surgical management and strategies in the treatment of hypothermia and cold injury | No | Excluded at title/ abstract screening | |
| 680 | 2009 | Pfeifer, C. | On probabilities of avalanches triggered by alpine skiers. An empirically driven decision strategy for backcountry skiers based on these probabilities | No | No relevant statistics obtained | |
| 681 | 2018 | Pfeifer, C.; Höller, P.; Zeileis, A. | Spatial and temporal analysis of fatal off-piste and backcountry avalanche accidents in Austria with a comparison of results in Switzerland, France, Italy and the US | No | Excluded at title/ abstract screening | |
| 682 | 2019 | Pietsch U, Strapazzon G, Ambühl D, et al. | Challenges of helicopter mountain rescue missions by human external cargo: need for physicians onsite and comprehensive training | No | Irrelevant title/ abstract (reports from other methods) | |
| 683 | 2012 | Pigolkin, Iu I.; Mechukaev, A. A.; Mechukaev, A. M. | Analysis of violent deaths of mountaineers and tourists at high altitudes | No | Excluded at title/ abstract screening | |
| 684 | 1978 | Plafker, G.; Ericksen, G. E. | Nevados huascarán avalanches, peru | No | Excluded at title/ abstract screening | |
| 685 | 2014 | Platt, B. F.; Hasiotis, S. T. | Novel Neoichnology of Elephants: Nonlocomotive Interactions with Sediment, Locomotion Traces in Partially Snow-Covered Sediment, and Implications for Proboscidean Paleoichnology | No | Irrelevant title/ abstract (reports from other methods) | |
| 686 | 2014 | Podolskiy, Evgeny A.; Izumi, Kaoru; Suchkov, Vladimir E.; Eckert, Nicolas | Physical and societal statistics for a century of snow-avalanche hazards on Sakhalin and the Kuril Islands (1910-2010) | No | Excluded at title/ abstract screening | |

| **Appendix. Data screening record.** | | | | | | |
|---|---|---|---|---|---|---|
| **This table records inclusion and exclusion decisions (with reasons for exclusion) for 1031 studies/registries (750 unique ones from database search and 281 unique ones from reference screening and expert consultation); Reasons to exclude correspond to Fig 1 in the paper** | | | | | | |
| **Index** | **Publication Year** | **Author** | **Title** | **If included** | **Reason for exclusion** | **URL for grey included paper** |
| **687** | **2017** | Podsiadło, Paweł; Darocha, Tomasz; Kosiński, Sylweriusz; Sałapa, Kinga; Ziętkiewicz, Mirosław; Sanak, Tomasz; Turner, Rachel; Brugger, Hermann | Severe Hypothermia Management in Mountain Rescue: A Survey Study | No | No relevant statistics obtained | |
| **688** | **2014** | Pokharel, Krishna | Sherpas Discuss Canceling Climbs | No | Excluded at title/ abstract screening | |
| **689** | **1988** | Pollard A, Clarke | Death during mountaineering at extreme altitude | No | Irrelevant title/ abstract (reports from other methods) | |
| **690** | **1998** | Polloni, G.; Aleotti, P.; Presbitero, M. | Terraced slopes in mountain areas: Sliding risk and protective measures in Valtellina, Italy | No | Irrelevant title/ abstract (reports from other methods) | |
| **691** | **2015** | Popplewell, Brett | THE APOCALYPSE ON EVEREST | No | Excluded at title/ abstract screening | |
| **692** | **2022** | Posch M, Burtscher J, Ruedl G, Pococco E, Burtscher M. | Unchanged fatality rate on Austrian ski slopes during the COVID-19 lockdown. | No | Irrelevant title/ abstract (reports from other methods) | |
| **693** | **2017** | Povoledo, Elisabetta | Italy Faces More Sorrow As Helicopter Goes Down | No | Excluded at title/ abstract screening | |
| **694** | **1999** | Povoledo, Elizabeth | In the Alps, Precautions Against Avalanches | No | Excluded at title/ abstract screening | |
| **695** | **2014** | Prato, F.; Cavoretto, L.; Governo, E.; Giardini, G.; Visetti, E. | A new active device to breathe during snow burial: A pilot study | No | Excluded at title/ abstract screening | |
| **696** | **2016** | Procter E, Strapazzon G, Dal Cappello T. | Burial duration, depth and air pocket explain avalanche survival patterns in Austria and Switzerland. | No | No relevant statistics obtained | |
| **697** | **2014** | Procter, E.; Strapazzon, G.; Dal Cappello, T.; Castlunger, L.; Staffler, H. P.; Brugger, H. | Adherence of backcountry winter recreationists to avalanche prevention and safety practices in northern Italy | No | Excluded at title/ abstract screening | |
| **698** | **2014** | Procter, Emily; Strapazzon, Giacomo; Renner, Andreas; Würtele, Andreas; Bilek, Hanno; Falk, Markus; Brugger, Hermann | Proceedings, International Snow Science Workshop, Banff, 2014 | No | Irrelevant title/ abstract (reports from other methods) | |
| **699** | **2012** | Püntener, C.; Stoffel, M.; Schneuwly-Bollschweiler, M. | Frequency and flow heights in the Plattlaui avalanche couloir (Uri) - A reconstruction based on tree growth rings | No | Excluded at title/ abstract screening | |

| Appendix. Data screening record. | | | | | | |
|---|---|---|---|---|---|---|
| **This table records inclusion and exclusion decisions (with reasons for exclusion) for 1031 studies/registries (750 unique ones from database search and 281 unique ones from reference screening and expert consultation); Reasons to exclude correspond to Fig 1 in the paper** | | | | | | |
| **Index** | **Publication Year** | **Author** | **Title** | **If included** | **Reason for exclusion** | **URL for grey included paper** |
| **700** | **1995** | Purkayastha SS, Ray US, Arora BS, et al. | Acclimatization at high altitude in gradual and acute induction | No | Irrelevant title/ abstract (reports from other methods) | |
| **701** | **2008** | Purvis, Susan; Burnett, Patti | IMPROVING K-9 AVALANCHE TEAM PERFORMANCE AND COMMUNICATION IN BACKCOUNTRY AVALANCHE MISSIONS: A COLLABORATIVE APPROACH BETWEEN SKI PATROL AND K-9 SEARCH AND RESCUE (SAR) TEAMS | No | Irrelevant title/ abstract (reports from other methods) | |
| **702** | **2010** | Putzer G, Schmid S, Braun P, Brugger H, Paal P | Cooling of six centigrades in an hour during avalanche burial | No | Irrelevant title/ abstract (reports from other methods) | |
| **703** | **2015** | Pysny, L.; Petru, D.; Pysna, J. | Selected Aspects of The First Aid During Teaching of Physical Education and Sport in Winter Activities | No | Excluded at title/ abstract screening | |
| **704** | **2014** | Qiu, Jane | Avalanche hotspot revealed | No | Excluded at title/ abstract screening | |
| **705** | **2018** | Quéno, Louis; Vionnet, Vincent; Cabot, Frédéric; Vrécourt, Dominique; Dombrowski-Etchevers, Ingrid | Forecasting and modelling ice layer formation on the snowpack due to freezing precipitation in the Pyrenees | No | Excluded at title/ abstract screening | |
| **706** | **1976** | Quervain de M | Ffir den Lawinenunfallwese ntliche Lawineneigenschaft en. In: Lawinen. Tagung fiber die medizinischen Aspekte des Lawinenunfalles. Juris Druck und Vetlag, | No | Irrelevant title/ abstract (reports from other methods) | |
| **707** | **1998** | R. A. Massom, V. I. Lytle, • A. P. Worby, • and I. Allison • | Winter snow cover variability on East Antarctic sea ice | No | Irrelevant title/ abstract (reports from other methods) | |
| **708** | **2008** | Rabl, W.; Pavlic, M.; Sumann, G. | Avalanche accidents | No | No relevant statistics obtained | |
| **709** | **2001** | Radwin MI, Grissom CK, Scholand MB, Harmston CH. | Normal oxygenation and ventilation during snow burial by the exclusion of exhaled carbon dioxide. | No | Irrelevant title/ abstract (reports from other methods) | |
| **710** | **2008** | Radwin, M. I. | Unburying the facts about avalanche victim pathophysiology | No | All relevant statistics were cited elsewhere | |
| **711** | **2002** | Radwin, Martin I.; Grissom, Colin K. | Technological advances in avalanche survival | No | No relevant statistics obtained | |

| **Appendix. Data screening record.** | | | | | | |
|---|---|---|---|---|---|---|
| **This table records inclusion and exclusion decisions (with reasons for exclusion) for 1031 studies/registries (750 unique ones from database search and 281 unique ones from reference screening and expert consultation); Reasons to exclude correspond to Fig 1 in the paper** | | | | | | |
| **Index** | **Publication Year** | **Author** | **Title** | **If included** | **Reason for exclusion** | **URL for grey included paper** |
| 712 | 2014 | Rahman, M. A. | Enabling drone communications with WiMAX Technology | No | Excluded at title/ abstract screening | |
| 713 | 2019 | Rahmati, Omid; Yousefi, Saleh; Kalantari, Zahra; Uuemaa, Evelyn; Teimurian, Teimur; Keesstra, Saskia; Pham, Tien Dat; Tien Bui, Dieu | Multi-Hazard Exposure Mapping Using Machine Learning Techniques: A Case Study from Iran | No | Excluded at title/ abstract screening | |
| 714 | 2008 | Rainer, Bernhard; Frimmel, Christian; Sumann, Guenther; Brugger, Hermann; Kinzl, Johann F.; Lederer, Wolfgang | Correlation between avalanche emergencies and avalanche danger forecast in the alpine region of Tyrol | No | Excluded at title/ abstract screening | |
| 715 | 2015 | Raley, Brad F. | Encounters in Avalanche Country: A History of Survival in the Mountain West, 1821-1920 | No | Excluded at title/ abstract screening | |
| 716 | 2006 | Rammig, A.; Fahse, L.; Bugmann, H.; Bebi, P. | Forest regeneration after disturbance: A modelling study for the Swiss Alps | No | Excluded at title/ abstract screening | |
| 717 | 2021 | Rapisarda, Aurora; Pranzo, Andrea Marco Raffaele | Mapping the avalanche risk: from survey to cartographic production. The avalanche bulletin of the Meteomont Service of the Alpine Troops Command | No | Excluded at title/ abstract screening | |
| 718 | 2018 | Rauch S, Dal Cappello T, Strapazzon G, et al. | Pre-hospital times and clinical characteristics of severe trauma patients: a comparison between mountain and urban/ suburban areas. | No | Irrelevant title/ abstract (reports from other methods) | |
| 719 | 2021 | Rauch, S.; Strapazzon, G.; Brugger, H. | On-site medical management of avalanche victims—a narrative review | No | No relevant statistics obtained | |
| 720 | 2022 | Rauch, Simon; Koppenberg, Joachim; Josi, Dario; Meuli, Lorenz; Strapazzon, Giacomo; Pasquier, Mathieu; Albrecht, Roland; Brugger, Hermann; Zweifel, Benjamin; Pietsch, Urs | Avalanche survival depends on the time of day of the accident: A retrospective observational study | No | No relevant statistics obtained | |
| 721 | 2006 | Read, J.; Stokes, A. | Plant biomechanics in an ecological context | No | Excluded at title/ abstract screening | |
| 722 | 1986 | Reid WA, Doyle D, Richmond HG, Galbraith SL” | Necropsy study of mountaineering accidents in Scotland | No | Irrelevant title/ abstract (reports from other methods) | |

| Appendix. Data screening record. | | | | | | |
|---|---|---|---|---|---|---|
| This table records inclusion and exclusion decisions (with reasons for exclusion) for 1031 studies/registries (750 unique ones from database search and 281 unique ones from reference screening and expert consultation); Reasons to exclude correspond to Fig 1 in the paper | | | | | | |
| **Index** | **Publication Year** | **Author** | **Title** | **If included** | **Reason for exclusion** | **URL for grey included paper** |
| **723** | **2016** | Reilly, M. J.; Spies, T. A. | Disturbance, tree mortality, and implications for contemporary regional forest change in the Pacific Northwest | No | Excluded at title/ abstract screening | |
| **724** | **2017** | Reiweger, Ingrid; Genswein, Manuel; Paal, Peter; Schweizer, Jürg | A concept for optimizing avalanche rescue strategies using a Monte Carlo simulation approach | No | Excluded at title/ abstract screening | |
| **725** | **2006** | Resler, L. M. | Geomorphic controls of spatial pattern and process at alpine treeline | No | Excluded at title/ abstract screening | |
| **726** | **2023** | Reuter, Benjamin; Hagenmuller, Pascal; Eckert, Nicolas | 62 YEARS OF AVALANCHE PROBLEMS IN THE FRENCH ALPS - CLIMATE AND TRENDS | No | Excluded at title/ abstract screening | |
| **727** | **2009** | Reuter, Benjamin; Schweizer, JÃ¼rg | Avalanche Triggering by Sound: Myth and Truth | No | Excluded at title/ abstract screening | |
| **728** | **2010** | Rheam, Mike; Comey, Bob | The Big Loss: JHSPâ€™s Avalanche Mitigation Fatality and the Resulting Program Changes | No | Excluded at title/ abstract screening | |
| **729** | **2011** | Rheinberger, C. M. | A Mixed Logit Approach to Study Preferences for Safety on Alpine Roads | No | Irrelevant title/ abstract (reports from other methods) | |
| **730** | **2009** | Rheinberger, Christoph M.; Bründl, Michael; Rhyner, Jakob | Dealing with the white death: avalanche risk management for traffic routes | No | No relevant statistics obtained | |
| **731** | **2012** | Rico, J.; Bataller, V.; Rueda, C.; Olmedo, R.; Sancho, J.; Villarroel, J. L.; Fernández, A. J.; Pérez, J.; Diez, J. | Cooperative system for avalanche rescue | No | Excluded at title/ abstract screening | |
| **732** | **1983** | Ridden, J. M. C. | An estimate of the fatal accident frequency rate from mountaineering fatalities in Peru | No | All relevant statistics were cited elsewhere | |
| **733** | **1999** | Roach, Mary | Avalanche! | No | Excluded at title/ abstract screening | |
| **734** | **2014** | Robine, Keith | In Search of a Human Factors Decision Making Tool | No | Excluded at title/ abstract screening | |
| **735** | **1993** | Rostrup M, Gilbert M | Avalanche accidents | No | No relevant data (full text examined, papers through other methods) | |
| **736** | **1993** | Rostrup, M.; Gilbert, M. | Avalanche accidents | No | Excluded at title/ abstract screening | |

| **Appendix. Data screening record.** | | | | | | |
|---|---|---|---|---|---|---|
| **This table records inclusion and exclusion decisions (with reasons for exclusion) for 1031 studies/registries (750 unique ones from database search and 281 unique ones from reference screening and expert consultation); Reasons to exclude correspond to Fig 1 in the paper** | | | | | | |
| **Index** | **Publication Year** | **Author** | **Title** | **If included** | **Reason for exclusion** | **URL for grey included paper** |
| **737** | **1989** | Rostrup, M.; Gilbert, M.; Stalsberg, H. | A snow avalanche in Vassdalen. Medical experiences | No | No relevant statistics obtained | |
| **738** | **2022** | Roubik, K.; Sykora, K.; Sieger, L.; Ort, V.; Horakova, L.; Walzel, S. | Perlite is a suitable model material for experiments investigating breathing in high density snow | No | Excluded at title/ abstract screening | |
| **739** | **2020** | Roubik, K.; Walzel, S.; Horakova, L.; Refalo, A.; Sykora, K.; Ort, V.; Sieger, L. | Materials suitable to simulate snow during breathing experiments for avalanche survival research | No | Excluded at title/ abstract screening | |
| **740** | **2015** | Roubík, Karel; Sieger, Ladislav; Sykora, Karel | Work of Breathing into Snow in the Presence versus Absence of an Artificial Air Pocket Affects Hypoxia and Hypercapnia of a Victim Covered with Avalanche Snow: A Randomized Double Blind Crossover Study | No | Excluded at title/ abstract screening | |
| **741** | **2015** | Rudolf-Miklau, F.; Sauermoser, S.; Mears, A. I. | The Technical Avalanche Protection Handbook | No | Excluded at title/ abstract screening | |
| **742** | **2020** | Rudyi, R. M.; Kyselov, Y. O.; Domashenko, H. T.; Kravets, O. Y.; Husar, K. D. | Analysis of Mountain Relief for the Causes of Snow Avalanches | No | Excluded at title/ abstract screening | |
| **743** | **2011** | Ruedl G, Bilek H, Ebner H, et al. | Fatalities on Austrian ski slopes during a 5-year period. | No | Irrelevant title/ abstract (reports from other methods) | |
| **744** | **2018** | Rugg CD, Malzacher T, Ausserer J, et al. | Gender differences in snowboarding accidents in Austria: a 2005-2018 registry analysis. | No | Irrelevant title/ abstract (reports from other methods) | |
| **745** | **2011** | Ruidiaz, M. E.; Cortes-Mateos, M. J.; Sandoval, S.; Martin, D. T.; Wang-Rodriguez, J.; Hasteh, F.; Wallace, A.; Vose, J. G.; Kummel, A. C.; Blair, S. L. | Quantitative Comparison of Surgical Margin Histology Following Excision With Traditional Electrosurgery and a Low-Thermal-Injury Dissection Device | No | Irrelevant title/ abstract (reports from other methods) | |
| **746** | **2010** | Russell K, Christie J, Hagel BE. | The effect of helmets on the risk of head and neck injuries among skiers and snowboarders: a meta-analysis | No | Irrelevant title/ abstract (reports from other methods) | |

| Appendix. Data screening record. | | | | | | |
|---|---|---|---|---|---|---|
| This table records inclusion and exclusion decisions (with reasons for exclusion) for 1031 studies/registries (750 unique ones from database search and 281 unique ones from reference screening and expert consultation); Reasons to exclude correspond to Fig 1 in the paper | | | | | | |
| **Index** | **Publication Year** | **Author** | **Title** | **If included** | **Reason for exclusion** | **URL for grey included paper** |
| **747** | **2013** | Rybarczyk, Y.; Vernay, D.; Rybarczyk, P.; Lebret, M. C.; Duhaut, D.; Lemasson, G.; Pestye, S.; Lucidarme, P. | COCHISE project: An augmented service dog for disabled people | No | Excluded at title/ abstract screening | |
| **748** | **2014** | Sachs, J.; Helbig, M.; Herrmann, R.; Kmec, M.; Schilling, K.; Zaikov, E. | Remote vital sign detection for rescue, security, and medical care by ultra-wideband pseudo-noise radar | No | Excluded at title/ abstract screening | |
| **749** | **2019** | Sadeghiamirshahidi , M.; Vitton, S. J. | Tropical storm-induced landslide-dammed lakes and debris flow hazards at Ocotepeque, Western Honduras | No | Irrelevant title/ abstract (reports from other methods) | |
| **750** | **2007** | Safar P, Paradis NA, Weil MH. | Cardiac arrest – the science and practice of resuscitation medicine | No | Irrelevant title/ abstract (reports from other methods) | |
| **751** | **2010** | Sahn, Karen R. | Avalanche Risk Reduction in the Continental Climate: How to Implement an Effective Boot Packing Program | No | Excluded at title/ abstract screening | |
| **752** | **2010** | Salzer, Friedrich; Studeregger, Arnold | Climate Change in Lower Austria â€“ A Snow Cover Analysis of the Last 100 Years With a Special Emphasis on the Last Century and the Impact of the Avalanche Situation in Lower Austria | No | Excluded at title/ abstract screening | |
| **753** | **1993** | Sampalis JS, Lavoie A, Williams JI, et al. | Impact of on-site care, prehospital time, and level of in-hospital care on survival in severely injured patients. | No | Irrelevant title/ abstract (reports from other methods) | |
| **754** | **2017** | Sandoy, G.; Oppikofer, T.; Nilsen, B. | Why did the 1756 Tjellefonna rockslide occur? A back-analysis of the largest historic rockslide in Norway | No | Excluded at title/ abstract screening | |
| **755** | **1987** | Schaerer PA | Fatal accidents and property damage | No | Irrelevant title/ abstract (reports from other methods) | |
| **756** | **2014** | Schaffer, Grayson | SURFACING | No | Excluded at title/ abstract screening | |
| **757** | **1990** | Schaller, Marie-Denise; Fischer, Adam P.; Perret, Claude H.; Schaller, M. D.; Fischer, A. P.; Perret, C. H. | Hyperkalemia. A prognostic factor during acute severe hypothermia | No | No relevant statistics obtained | |
| **758** | **2003** | Schiermeier, Quirin | Disaster planning: Avalanche! | No | Excluded at title/ abstract screening | |

**Appendix. Data screening record.**

**This table records inclusion and exclusion decisions (with reasons for exclusion) for 1031 studies/registries (750 unique ones from database search and 281 unique ones from reference screening and expert consultation); Reasons to exclude correspond to Fig 1 in the paper**

| Index | Publication Year | Author | Title | If included | Reason for exclusion | URL for grey included paper |
|---|---|---|---|---|---|---|
| 759 | 1976 | Schild M | Problemstellung, Statistik, Standortbestimmung | No | Irrelevant title/ abstract (reports from other methods) | |
| 760 | 2017 | Schindelwig, Kurt; Hoffmann, Alexander; Mössner, Martin; Nachbauer, Werner | Does Avalanche Shovel Shape Affect Excavation Time: A Pilot Study | No | Excluded at title/ abstract screening | |
| 761 | 1981 | Schmid F | Zur Pathogenese des Lungen6dems nach Lawinenverschiittun | No | Irrelevant title/ abstract (reports from other methods) | |
| 762 | 1981 | Schmid F | The pathogenesis of pulmonary edema after being buried by an avalanche | No | Irrelevant title/ abstract (reports from other methods) | |
| 763 | 2012 | Schmutzhard, E.; Fischer, M.; Dietmann, A.; Helbok, R.; Broessner, G. | Rewarming: Facts and myths from the neurological perspectives | No | Excluded at title/ abstract screening | |
| 764 | 2011 | Schneider, D.; Huggel, C.; Haeberli, W.; Kaitna, R. | Unraveling driving factors for large rock-ice avalanche mobility | No | Irrelevant title/ abstract (reports from other methods) | |
| 765 | 1985 | Schnell, G.; Miüller, E.; Kern, H. | Beitrag zur Ätiologie des Triebsterbens junger Lärchen der subalpinen Stufe: II. Abiotische Ursachen | No | Excluded at title/ abstract screening | |
| 766 | 2009 | Schneuwly, Dominique M.; Stoffel, Markus; Bollschweiler, Michelle | Formation and spread of callus tissue and tangential rows of resin ducts in Larix decidua and Picea abies following rockfall impacts | No | Excluded at title/ abstract screening | |
| 767 | 2000 | Scholand, Mary Beth; Grissom, Colin K.; Radwin, Martin I.; Harmston, Chris H. | Hypothermia During Snow Burial | No | Excluded at title/ abstract screening | |
| 768 | 2020 | Schön CA, Gordon L, Hölzl N, et al | Determination of death in mountain rescue: recommendations of the International Commission for mountain emergency medicine (ICAR MedCom) | No | Irrelevant title/ abstract (reports from other methods) | |
| 769 | 2007 | Schuster, R. L.; Highland, L. M. | Overview of the effects of mass wasting on the natural environment | No | Excluded at title/ abstract screening | |
| 770 | 1996 | Schuster, R. L.; Nieto, A. S.; O'Rourke, T. D.; Crespo, E.; Plaza-Nieto, G. | Mass wasting triggered by the 5 March 1987 Ecuador earthquakes | No | Excluded at title/ abstract screening | |

| Appendix. Data screening record. | | | | | | |
|---|---|---|---|---|---|---|
| This table records inclusion and exclusion decisions (with reasons for exclusion) for 1031 studies/registries (750 unique ones from database search and 281 unique ones from reference screening and expert consultation); Reasons to exclude correspond to Fig 1 in the paper | | | | | | |
| **Index** | **Publication Year** | **Author** | **Title** | **If included** | **Reason for exclusion** | **URL for grey included paper** |
| 771 | 2002 | Schuster, R. L.; Salcedo, D. A.; Valenzuela, L. | Overview of catastrophic landslides of South America in the twentieth century | No | Excluded at title/abstract screening | |
| 772 | 2010 | Schweizer J, Bellaire S. | On stability sampling strategy at the slope scale. | No | Irrelevant title/abstract (reports from other methods) | |
| 773 | 2007 | Schweizer J, Jamieson JB. | A threshold sum approach to stability evaluation of manual snow profiles | No | Irrelevant title/abstract (reports from other methods) | |
| 774 | 2001 | Schweizer J, Wiesinger T. | Snow profile interpretation for stability evaluation | No | Irrelevant title/abstract (reports from other methods) | |
| 775 | 2014 | Schweizer, J. | On the frequency of avalanche incidents with multiple victims | No | Excluded at title/abstract screening | |
| 776 | 2001 | Schweizer, J.; Camponovo, C. | The skier's zone of influence in triggering slab avalanches | No | Excluded at title/abstract screening | |
| 777 | 2001 | Schweizer, J.; Lütschg, M. | Characteristics of human-triggered avalanches | No | No relevant statistics obtained | |
| 778 | 2006 | Schweizer, JÃ¼rg; Harvey, Stephan | Legal Consequences of Fatal Avalanche Accidents in the European Alps | No | Excluded at title/abstract screening | |
| 779 | 2019 | Scognamiglio, S.; Basile, A.; Calcaterra, D.; Iamarino, M.; Langella, G.; Moretti, P.; Vingiani, S.; Terribile, F. | Andic soils and flow-like landslides: Cause-effect evidence from Italy | No | Irrelevant title/abstract (reports from other methods) | |
| 780 | 2001 | Scott, K. M.; Macías, J. L.; Naranjo, J. A.; Rodríguez, S.; McGeehin, J. P. | Catastrophic debris flows transformed from landslides in volcanic terrains: Mobility, hazard assessment, and mitigation strategies | No | Excluded at title/abstract screening | |
| 781 | 2021 | Serey, A.; Sepulveda, S. A.; Murphy, W.; Petley, D. N.; De Pascale, G. | Developing conceptual models for the recognition of coseismic landslides hazard for shallow crustal and megathrust earthquakes in different mountain environments - an example from the Chilean Andes | No | Excluded at title/abstract screening | |
| 782 | 2011 | Serrouya, R.; McLellan, B. N.; Pavan, G. D.; Apps, C. D. | Grizzly Bear Selection of Avalanche Chutes: Testing the Effectiveness of Forest Buffer Retention | No | Irrelevant title/abstract (reports from other methods) | |

**Appendix. Data screening record.**

**This table records inclusion and exclusion decisions (with reasons for exclusion) for 1031 studies/registries (750 unique ones from database search and 281 unique ones from reference screening and expert consultation); Reasons to exclude correspond to Fig 1 in the paper**

| Index | Publication Year | Author | Title | If included | Reason for exclusion | URL for grey included paper |
|---|---|---|---|---|---|---|
| 783 | 2003 | Sharp, David | Celebrating Everest | No | Excluded at title/ abstract screening | |
| 784 | 2006 | Shealy JE, Johnson RJ, Ettlinger CF | On piste fatalities in recreational snow sports in the U.S | No | Irrelevant title/ abstract (reports from other methods) | |
| 785 | 1985 | Shealy JE. | Skiing trauma and safery: fifth international symposium. | | Irrelevant title/ abstract (reports from other methods) | |
| 786 | 1985 | Shealy, Jasper E. | DEATH IN DOWNHILL SKIING | No | No relevant statistics obtained | |
| 787 | 2018 | Sheets, A.; Wang, D. L.; Logan, S.; Atkins, D. | Causes of Death Among Avalanche Fatalities in Colorado: A 21-Year Review | Yes, for meta-analysis and systematic review | NA, since included | |
| 788 | 1996 | Shephard, R. J. | Asphyxial death of a young skier - Case report | No | Irrelevant title/ abstract (reports from other methods) | |
| 789 | 1987 | Sherry E, Asquith J. | Nordic (cross-country) skiing injuries in Australia. | No | Irrelevant title/ abstract (reports from other methods) | |
| 790 | 1988 | Sherry E, Clout L.. | Deaths associated with skiing in Australia: a 32 year study of cases from the Snowy Mountains. | No | No relevant data (full text examined, papers through other methods) | |
| 791 | 2018 | Shiba, K.; Hasegawa, M.; Aiura, H.; Matsuura, T.; Nakata, M. | Effect of wooden anti-glide tripods and influence of planting density of sugi (Cryptomeria japonica) on establishment of an avalanche protection forest in a high snow accumulation region | No | Excluded at title/ abstract screening | |
| 792 | 2021 | Shirota, T.; Iwasaki, C.; Okano, T.; Oya, S. | NATURAL REGENERATION AND ARTIFICIAL THINNING FOR EARLY FOREST RESTORATION ON A PERMANENTLY CLOSED SKI SLOPE | No | Excluded at title/ abstract screening | |
| 793 | 1989 | Shlim DR, Houston R” | Helicopter rescues and deaths among trekkers in Nepal | No | Irrelevant title/ abstract (reports from other methods) | |
| 794 | 2014 | Sianes, X. S.; Rodes, P. | A comparative study about training on avalanche rescue | No | Excluded at title/ abstract screening | |
| 795 | 2007 | Silverton, Natalie A.; McIntosh, Scott E.; Kim, Han S. | Avalanche safety practices in Utah | No | Excluded at title/ abstract screening | |
| 796 | 2020 | Singh, D. K.; Mishra, V. D.; Gusain, H. S. | Simulation and Analysis of a Snow Avalanche Accident in Lower Western Himalaya, India | No | Excluded at title/ abstract screening | |

| **Appendix. Data screening record.** | | | | | | |
|---|---|---|---|---|---|---|
| **This table records inclusion and exclusion decisions (with reasons for exclusion) for 1031 studies/registries (750 unique ones from database search and 281 unique ones from reference screening and expert consultation); Reasons to exclude correspond to Fig 1 in the paper** | | | | | | |
| **Index** | **Publication Year** | **Author** | **Title** | **If included** | **Reason for exclusion** | **URL for grey included paper** |
| 797 | 2004 | Singhroy, V.; Molch, K. | Characterizing and monitoring rockslides from SAR techniques | No | Excluded at title/ abstract screening | |
| 798 | 2013 | Sinickas, Alexandra; Jamieson, Bruce | Validating a Relationship Between Avalanche Runout Distance and Frequency | No | Excluded at title/ abstract screening | |
| 799 | 2004 | Siu TLT, Chandran KN, Newcombe RL, Fuller JW, Pik JHT | Snow sports related head and spinal injuries: an eight-year survey from the neurotrauma centre for the snowy mountains, | No | Irrelevant title/ abstract (reports from other methods) | |
| 800 | 2016 | Skaiaa, S. C.; Thomassen, O. | Avalanche Accidents and treatment of victim | No | No relevant statistics obtained | |
| 801 | 2005 | Skutlaberg, S. M. I. | When do we carry out permanent safety improvements against avalanches and rockfalls along the Norwegian road network? | No | Irrelevant title/ abstract (reports from other methods) | |
| 802 | 2019 | Slaton, M. R.; MacKenzie, M.; Kohler, T.; Ramirez, C. M. | Whitebark Pine Recruitment in Sierra Nevada Driven by Range Position and Disturbance History | No | Excluded at title/ abstract screening | |
| 803 | 2009 | Slaughter, Andrew E.; McCabe, Doug; Munter, Henry; Staron, Patrick J.; Adams, Edward E.; Catherine, Doug; Henninger, Irene; Cooperstein, Mike; Leonard, Tom | An investigation of radiation-recrystallization coupling laboratory and field studies | No | Excluded at title/ abstract screening | |
| 804 | 2005 | Slotta-Bachmayr, L. | How burial time of avalanche victims is influenced by rescue method: An analysis of search reports from the alps | No | No relevant statistics obtained | |
| 805 | 1988 | Smith, K. | Avalanche hazards: the rising death toll | No | No relevant statistics obtained | |
| 806 | 2003 | Solomon, Christopher | Kelly's Last Ride | No | Excluded at title/ abstract screening | |
| 807 | 2006 | Solomon, Christopher | THE DANGEROUS GAME. (Cover story) | No | Excluded at title/ abstract screening | |
| 808 | 2018 | Solomon, Christopher | A TERROR WAY BEYOND FALLING | No | Excluded at title/ abstract screening | |
| 809 | 2007 | Søreide K, Krüger AJ, Vårdal AL, et al. | Epidemiology and contemporary patterns of trauma deaths: changing place, similar PACE, older face. | No | Irrelevant title/ abstract (reports from other methods) | |
| 810 | 2016 | Sorensen, Chris | Trails of destruction | No | Excluded at title/ abstract screening | |

| Appendix. Data screening record. | | | | | | |
|---|---|---|---|---|---|---|
| This table records inclusion and exclusion decisions (with reasons for exclusion) for 1031 studies/registries (750 unique ones from database search and 281 unique ones from reference screening and expert consultation); Reasons to exclude correspond to Fig 1 in the paper | | | | | | |
| **Index** | **Publication Year** | **Author** | **Title** | **If included** | **Reason for exclusion** | **URL for grey included paper** |
| 811 | | Soulé, B.; Lefèvre, B.; Boutroy, E. | The dangerousness of mountain recreation: A quantitative overview of fatal and non-fatal accidents in France | No | Irrelevant title/ abstract (reports from other methods) | |
| 812 | 2017 | Soule, B.; Reynier, V.; Lefevre, B.; Boutroy, E. | Who is at risk in the French mountains? Profiles of the accident victims in outdoor sports and mountain recreation | No | Excluded at title/ abstract screening | |
| 813 | 2008 | Soule, P. B.; Lebihain, P. | Post-accident analysis of an avalanche fatality | No | No relevant statistics obtained | |
| 814 | 2011 | Spencer, J. M.; Ashley, W. S. | Avalanche fatalities in the western United States: A comparison of three databases | No | No relevant statistics obtained | |
| 815 | 1994 | Spitz WU, Fisher RS | Medicolegal investigation of death | No | Excluded at title/ abstract screening | |
| 816 | 1989 | Stalsberg, H.; Albretsen, C.; Gilbert, M.; Kearney, M.; Moestue, E.; Nordrum, I.; Rostrup, M.; Orbo, A. | Mechanism of death in avalanche victims | Yes, for meta-analysis and systematic review | NA, since included | |
| 817 | 2018 | Statham, G., P. Haegeli, E. Greene, K. Birkeland, C. Israelson, B. Tremper, C. Stethem, B. McMahon, B. White, and J. Kelly, | A conceptual model of avalanche hazard, Natural Hazards | No | Irrelevant title/ abstract (reports from other methods) | |
| 818 | 2006 | Statham, Grant; Claude, Louise; DeschÃªnes, Marc; Gagnon, StÃ©phane; Gautron, Lise; Kelly, John | Translation of Public Avalanche Bulletins | No | Excluded at title/ abstract screening | |
| 819 | 2011 | Stech, Katy; Radnofsky, Louise | U.S. WATCH | No | Excluded at title/ abstract screening | |
| 820 | 1997 | Stepanek, J.; Claypool, D. W. | GPS signal reception under snow cover: a pilot study establishing the potential usefulness of GPS in avalanche search and rescue operations | No | Excluded at title/ abstract screening | |
| 821 | 2003 | Stethem C, Jamieson B, Schaerer P, et al. | Snow avalanche hazard in Canada — a review. | | Irrelevant title/ abstract (reports from other methods) | |
| 822 | 2014 | Stewart-Patterson, Iain | Role of intuition in the decision process of expert ski guides | No | Excluded at title/ abstract screening | |

| Appendix. Data screening record. | | | | | | |
|---|---|---|---|---|---|---|
| **This table records inclusion and exclusion decisions (with reasons for exclusion) for 1031 studies/registries (750 unique ones from database search and 281 unique ones from reference screening and expert consultation); Reasons to exclude correspond to Fig 1 in the paper** | | | | | | |
| **Index** | **Publication Year** | **Author** | **Title** | **If included** | **Reason for exclusion** | **URL for grey included paper** |
| 823 | 2006 | Stoffel, Andreas; Foppa, Nando; Raderschall, Norbert | Mapping Snow Depth in the Alps | No | Excluded at title/ abstract screening | |
| 824 | 2006 | Stoffel, M.; Bollschweiler, M.; Hassler, G. R. | Differentiating past events on a cone influenced by debris-flow and snow avalanche activity - A dendrogeomorphol ogical approach | No | Irrelevant title/ abstract (reports from other methods) | |
| 825 | 2014 | Stoffel, M.; Corona, C. | DENDROECOLOGI CAL DATING OF GEOMORPHIC DISTURBANCE IN TREES | No | Excluded at title/ abstract screening | |
| 826 | 2008 | Stoffel, Markus; Hitz, Oliver M. | Rockfall and snow avalanche impacts leave different anatomical signatures in tree rings of juvenile Larix decidua | No | Excluded at title/ abstract screening | |
| 827 | 2006 | Stokes, Rebecca | THE PATROLLER: BILLY RANKIN | No | Excluded at title/ abstract screening | |
| 828 | 2006 | Story, Rob | Welcome to Brokebutt Mountain | No | Excluded at title/ abstract screening | |
| 829 | 2012 | Strapazzon G, Beikircher W, Procter E, Brugger H. | Electrical heart activity recorded during prolonged avalanche burial | No | Irrelevant title/ abstract (reports from other methods) | |
| 830 | 2012 | Strapazzon G, Nardin M, Zanon P, Kaufmann M, Kritzinger M, Brugger H | Respiratory failure and spontaneous hypoglycemia during noninvasive rewarming from 24.7 ○C (76.5 ○F) core body temperature after prolonged avalanche burial. | No | Irrelevant title/ abstract (reports from other methods) | |
| 831 | 2018 | Strapazzon, G.; Brugger, H. | On-Site Treatment of Snow Avalanche Victims: From Bench to Mountainside | No | No relevant statistics obtained | |
| 832 | 2021 | Strapazzon, G.; Gatterer, H.; Falla, M.; Dal Cappello, T.; Malacrida, S.; Turner, R.; Schenk, K.; Paal, P.; Falk, M.; Schweizer, J.; Brugger, H. | Hypoxia and hypercapnia effects on cerebral oxygen saturation in avalanche burial: A pilot human experimental study | No | No relevant statistics obtained | |
| 833 | 2021 | Strapazzon, G.; Putzer, G.; Dal Cappello, T.; Falla, M.; Braun, P.; Falk, M.; Glodny, B.; Pinggera, D.; Helbok, R.; Brugger, H. | Effects of hypothermia, hypoxia, and hypercapnia on brain oxygenation and hemodynamic parameters during simulated avalanche burial: a porcine study | No | Study a single cause of death | |

| Appendix. Data screening record. | | | | | | |
|---|---|---|---|---|---|---|
| This table records inclusion and exclusion decisions (with reasons for exclusion) for 1031 studies/registries (750 unique ones from database search and 281 unique ones from reference screening and expert consultation); Reasons to exclude correspond to Fig 1 in the paper | | | | | | |
| **Index** | **Publication Year** | **Author** | **Title** | **If included** | **Reason for exclusion** | **URL for grey included paper** |
| 834 | 2020 | Strapazzon, Giacomo; Brugger, Hermann; Paal, Peter; Brown, Doug | Reconsidering the air pocket around mouth and nose as a positive outcome predictor in completely buried avalanche victims | No | No relevant statistics obtained | |
| 835 | 2018 | Strapazzon, Giacomo; Migliaccio, Daniel; Fontana, Diego; Stawinoga, Agnieszka Elzbieta; Milani, Mario; Brugger, Hermann | Knowledge of the Avalanche Victim Resuscitation Checklist and Utility of a Standardized Lecture in Italy | No | No relevant statistics obtained | |
| 836 | 2018 | Strapazzon, Giacomo; Migliaccio, Daniel; Fontana, Diego; Stawinoga, Agnieszka Elzbieta; Milani, Mario; Brugger, Hermann | Knowledge of the Avalanche Victim Resuscitation Checklist and Utility of a Standardized Lecture in Italy | No | Irrelevant title/ abstract (reports from other methods) | |
| 837 | 2017 | Strapazzon, Giacomo; Paal, Peter; Schweizer, Jürg; Falk, Markus; Reuter, Benjamin; Schenk, Kai; Gatterer, Hannes; Grasegger, Katharina; Dal Cappello, Tomas; Malacrida, Sandro; Riess, Lukas; Brugger, Hermann | Effects of snow properties on humans breathing into an artificial air pocket - an experimental field study | No | Excluded at title/ abstract screening | |
| 838 | 2021 | Strapazzon, Giacomo; Schweizer, Jürg; Chiambretti, Igor; Brodmann Maeder, Monika; Brugger, Hermann; Zafren, Ken | Effects of Climate Change on Avalanche Accidents and Survival | No | No relevant statistics obtained | |
| 839 | 2009 | Streit, Kathrin; Wunder, Jan; Brang, Peter | Slit-shaped gaps are a successful silvicultural technique to promote Picea abies regeneration in mountain forests of the Swiss Alps | No | Excluded at title/ abstract screening | |
| 840 | 2006 | Strouthopoulos, Chris | Total Immersion | No | Excluded at title/ abstract screening | |
| 841 | 2015 | Stu, Woo | HEARD ON THE FIELD | No | Excluded at title/ abstract screening | |
| 842 | 2017 | Su, L. J.; Hu, K. H.; Zhang, W. F.; Wang, J.; Lei, Y.; Zhang, C. L.; Cui, P.; Pasuto, A.; Zheng, Q. H. | Characteristics and triggering mechanism of Xinmo landslide on 24 June 2017 in Sichuan, China | No | Excluded at title/ abstract screening | |
| 843 | 2021 | Subramanyan, Raghavan | Avalanches in cardiology | No | Excluded at title/ abstract screening | |

| Appendix. Data screening record. | | | | | | |
|---|---|---|---|---|---|---|
| This table records inclusion and exclusion decisions (with reasons for exclusion) for 1031 studies/registries (750 unique ones from database search and 281 unique ones from reference screening and expert consultation); Reasons to exclude correspond to Fig 1 in the paper | | | | | | |
| **Index** | **Publication Year** | **Author** | **Title** | **If included** | **Reason for exclusion** | **URL for grey included paper** |
| 844 | 2020 | Sumann G, Moens D, Brink B, et al. | Multiple trauma management in mountain environments - a scoping review : Evidence based guidelines of the International Commission for Mountain Emergency Medicine (ICAR MedCom). | No | Irrelevant title/ abstract (reports from other methods) | |
| 845 | 2012 | Sumann G, Putzer G, Brugger H, Paal P. | Pulmonary edema after complete avalanche burial. | No | Irrelevant title/ abstract (reports from other methods) | |
| 846 | 2020 | Surve, J.; Mehta, V.; Rawat, A.; Kamaliya, K.; Deb, D. | Low-Cost 2 MHz Transmitter for the Detection of Human Trapped Under the Snow | No | Irrelevant title/ abstract (reports from other methods) | |
| 847 | 2015 | Sutterlin, Chester E., 3rd | Spine surgery in Nepal: the 2015 earthquake | No | Excluded at title/ abstract screening | |
| 848 | 2014 | Sward, Douglas G.; Bennett, Brad L. | Wilderness medicine | No | Excluded at title/ abstract screening | |
| 849 | 2010 | Sweet, David M. | Implications of Recent Research in the Fields of Evolutionary Psychology and Cognitive Biology for Avalanche Safety Education | No | Excluded at title/ abstract screening | |
| 850 | 2020 | Sytnik, O.; Vyzmitinov, I. | Probabilistic measure for description of marks of alive human in signals of radar for rescuer | No | Excluded at title/ abstract screening | |
| 851 | 2010 | Talon, A.; Muzeau, J. P.; Carlier, J. Ph | Avalanche actions on constructions | No | Excluded at title/ abstract screening | |
| 852 | 2024 | Tanaka, Tanaka | Characteristics of Fatal Accidents due to Exogenous Causes at Ski Resorts in Japan Over the Past 13 Years: A Retrospective Descriptive Study | Yes, only for systematic review | NA, since included | |
| 853 | 1974 | Teasdale G, Jennett B. | Assessment of coma and impaired consciousness: a practical scale. | No | Irrelevant title/ abstract (reports from other methods) | |
| 854 | 2013 | Techel, Frank; Zweifel, Benjamin | Recreational Avalanche Accidents in Switzerland: Trends and Patterns With an Emphasis on Burial, Rescue Methods and Avalanche Danger | Yes, only for systematic review | NA, since included | https://arc.lib.montana.edu/snow-science/item.php?id=1844 |

**Appendix. Data screening record.**

**This table records inclusion and exclusion decisions (with reasons for exclusion) for 1031 studies/registries (750 unique ones from database search and 281 unique ones from reference screening and expert consultation); Reasons to exclude correspond to Fig 1 in the paper**

| Index | Publication Year | Author | Title | If included | Reason for exclusion | URL for grey included paper |
|---|---|---|---|---|---|---|
| 855 | 2018 | Tedim, Fantina; Leone, Vittorio | The deadly avalanche of Rigopiano (Itália): evidences of a constructed local scale disaster | No | Irrelevant title/ abstract (reports from other methods) | |
| 856 | 2019 | Teich, M.; Giunta, A. D.; Hagenmuller, P.; Bebi, P.; Schneebeli, M.; Jenkins, M. J. | Effects of bark beetle attacks on forest snowpack and avalanche formation - Implications for protection forest management | No | Excluded at title/ abstract screening | |
| 857 | 2012 | Teich, Michaela; Zurbriggen, Natalie; Bartelt, Perry; GrÃªt-Regamey, Adrienne; Marty, Christoph; Ulrich, Melanie; Bebi, Peter | Potential Impacts of Climate Change on Snow Avalanches Starting in Forested Terrain | No | Excluded at title/ abstract screening | |
| 858 | 2018 | Tengesdal, S. M.; Kruke, B. I. | Urban avalanche search and rescue operations in Longyearbyen: A study of public-private cooperation | No | No relevant statistics obtained | |
| 859 | 2002 | Tennant, D. J.; Rich, T. C. G. | Distribution maps and IUCN threat categories for Hieracium section Alpina (Asteraceae) in Britain | No | Excluded at title/ abstract screening | |
| 860 | 2003 | Thali MJ, Yen K, Schweitzer W, Vock P, Boesch C, Ozdoba C, et al. | Virtopsy, a new imaging horizon in forensic pathology: virtual autopsy by postmortem multislice computed tomography (MSCT) and magnetic resonance imaging (MRI)--a feasibility study | No | Irrelevant title/ abstract (reports from other methods) | |
| 861 | 2012 | The Associated, Press | Two Die After Being Caught in Avalanches | No | No relevant statistics obtained | |
| 862 | Registry | The WSL Institute for Snow and Avalanche Researc | Avalanche accidents in Switzerland | No | Data already covered | |
| 863 | 1996 | Thomas, Susan | A view to die | No | Excluded at title/ abstract screening | |
| 864 | 2013 | Thumlert, Scott; Exner, Thomas; Jamieson, Bruce; Bellaire, Sascha | Measurements of localized dynamic loading in a mountain snow cover | No | Excluded at title/ abstract screening | |
| 865 | 2015 | Thumlert, Scott; Jamieson, Bruce | Stress measurements from common snow slope stability tests | No | Excluded at title/ abstract screening | |
| 866 | 2014 | Thumlert, Scott; Jamieson, Bruce | Stress measurements in the snow cover below localized dynamic loads | No | Excluded at title/ abstract screening | |

| Appendix. Data screening record. | | | | | | |
|---|---|---|---|---|---|---|
| **This table records inclusion and exclusion decisions (with reasons for exclusion) for 1031 studies/registries (750 unique ones from database search and 281 unique ones from reference screening and expert consultation); Reasons to exclude correspond to Fig 1 in the paper** | | | | | | |
| **Index** | **Publication Year** | **Author** | **Title** | **If included** | **Reason for exclusion** | **URL for grey included paper** |
| **867** | **2019** | Thys, S.; Van Daele, M.; Praet, N.; Jensen, B. J. L.; Van Dyck, T.; Haeussler, P. J.; Vandekerkhove, E.; Cnudde, V.; De Batist, M. | Dropstones in Lacustrine Sediments as a Record of Snow Avalanches-A Validation of the Proxy by Combining Satellite Imagery and Varve Chronology at Kenai Lake (South-Central Alaska) | No | Excluded at title/ abstract screening | |
| **868** | **2022** | Tichavský, Radek; Fabiánová, Andrea; Koutroulis, Aristeidis; Spálovský, Vilém | Occasional but severe: Past debris flows and snow avalanches in the Helmos Mts. (Greece) reconstructed from tree-ring records | No | Excluded at title/ abstract screening | |
| **869** | **2015** | Time | Google Executive Killed in Everest Avalanche After Nepal Quake | No | Excluded at title/ abstract screening | |
| **870** | **2013** | Timiraos, Nick | Five Killed as Risk of Avalanche Mounts in the Rockies | No | No relevant statistics obtained | |
| **871** | **2005** | Tobe, K.; Li, X.; Omasa, K. | Effects of irrigation on seedling emergence and seedling survival of a desert shrub Haloxylon ammodendron (Chenopodiaceae) | No | Excluded at title/ abstract screening | |
| **872** | **2020** | Todea, Corina; Pop, Olimpiu Traian; Germain, Daniel | Snow-avalanche history reconstructed with tree rings in Parâng Mountains (Southern Carpathians, Romania) | No | Excluded at title/ abstract screening | |
| **873** | **2002** | Tolme, Paul | Snowballed | No | Excluded at title/ abstract screening | |
| **874** | **2003** | Tomazin I, Kovacs T. | Medical considerations in the use of helicopters in mountain rescue. | No | Irrelevant title/ abstract (reports from other methods) | |
| **875** | **2008** | Tonina, D.; Luce, C. H.; Rieman, B.; Buffington, J. M.; Goodwin, P.; Clayton, S. R.; Ali, S. M.; Barry, J. J.; Berenbrock, C. | Hydrological response to timber harvest in northern Idaho: implications for channel scour and persistence of salmonids | No | Irrelevant title/ abstract (reports from other methods) | |
| **876** | **1993** | Tough, S. C.; Butt, J. C. | A review of 19 fatal injuries associated with backcountry skiing | Yes, for meta-analysis and systematic review | NA, since included | |
| **877** | **2018** | Toutonghi, Pauls | THE HOUSE OF MOURNING | No | Excluded at title/ abstract screening | |
| **878** | **2001** | Tremper B | Staying Alive in Avalanche Terrain. | No | Irrelevant title/ abstract (reports from other methods) | |

| Appendix. Data screening record. | | | | | | |
|---|---|---|---|---|---|---|
| **This table records inclusion and exclusion decisions (with reasons for exclusion) for 1031 studies/registries (750 unique ones from database search and 281 unique ones from reference screening and expert consultation); Reasons to exclude correspond to Fig 1 in the paper** | | | | | | |
| **Index** | **Publication Year** | **Author** | **Title** | **If included** | **Reason for exclusion** | **URL for grey included paper** |
| 879 | 1993 | Tremper, Bruce | Life and death in snow country | No | No relevant statistics obtained | |
| 880 | 2014 | Trepet, S.; Eskina, T.; Bibina, K. | Influence of environmental factors on number dynamics and spatial structure of the tur ( Capra caucasica) population in the Caucasian Reserve | No | Excluded at title/ abstract screening | |
| 881 | 1999 | Tresniowski, Alex; Saveri, Gabrielle; Dodd, Johnny | Death on the Mountain | No | No relevant statistics obtained | |
| 882 | 2007 | Tropeano, D.; Turconi, L.; Fasana, G.; Nocente, V.; Pelissero, C.; Piatti, G.; Savio, G.; Mussino, S. | A multidisciplinary landscape analysis applied to the civil protection plan of the Orco and Soana Valleys (NW Italy) | No | Excluded at title/ abstract screening | |
| 883 | 2000 | Tschirky F, Brabec B, Kern M | Avalanche Rescue Systems in Switzerland: Experience and Limitations | No | No relevant data (full text examined, papers through other methods) | |
| 884 | 2001 | Tschirky F, Brabec B, Kern M | Avalanche rescue devices, state of the development, success and failure | No | No relevant data (full text examined, papers through other methods) | |
| 885 | 1996 | Tschirky F, Schweizer J. | Avalanche balloons — preliminary test results | No | Irrelevant title/ abstract (reports from other methods) | |
| 886 | 2013 | Tumajer, J.; Treml, V. | META-ANALYSIS OF DENDROCHRONO LOGICAL DATING OF MASS MOVEMENTS | No | Excluded at title/ abstract screening | |
| 887 | 1974 | Turnbull BW. | Nonparametric estimation of a survivorship function with doubly censored data | No | Irrelevant title/ abstract (reports from other methods) | |
| 888 | 2001 | Turnock, David; Carter, F. W. | Part III: Country studies: Chapter 17: Romania | No | Excluded at title/ abstract screening | |
| 889 | 1996 | Ueland, Jon | Avalanche Hazard Evaluation at Ski Areas | No | Excluded at title/ abstract screening | |
| 890 | 1999 | Ueno, S.; Hayashida, M. | Structure and dynamics of beech forests disturbed by surface avalanche on a heavy snow-fall slope | No | Excluded at title/ abstract screening | |
| 891 | 2001 | Ulmer, Kristen | Cliffhanger | No | Excluded at title/ abstract screening | |
| 892 | 2018 | Unterrader, S.; Almond, P.; Fuchs, S. | Rockfall in the Port Hills of Christchurch: Seismic and non-seismic fatality risk on roads | No | Excluded at title/ abstract screening | |

**Appendix. Data screening record.**

**This table records inclusion and exclusion decisions (with reasons for exclusion) for 1031 studies/registries (750 unique ones from database search and 281 unique ones from reference screening and expert consultation); Reasons to exclude correspond to Fig 1 in the paper**

| Index | Publication Year | Author | Title | If included | Reason for exclusion | URL for grey included paper |
|---|---|---|---|---|---|---|
| 893 | | Uphoff, Eleonora; Robertson, Lindsay; Cabieses, Baltica; Villalon, Francisco J.; Purgato, Marianna; Churchill, Rachel; Barbui, Corrado | An overview of systematic reviews on mental health promotion, prevention, and treatment of common mental disorders for refugees, asylum seekers, and internally displaced persons | No | Excluded at title/ abstract screening | |
| 894 | 2019 | Urabe, Y.; Takeuchi, T.; Sasadai, J.; Maeda, N. | Niseko resort changes Japanese ski safety. | No | Excluded at title/ abstract screening | |
| 895 | 2015 | Vacchiano, G.; Maggioni, M.; Perseghin, G.; Motta, R. | Effect of avalanche frequency on forest ecosystem services in a spruce-fir mountain forest | No | Excluded at title/ abstract screening | |
| 896 | 2012 | Vacek, S.; Hejcmanová, P.; Hejcman, M. | Vegetative reproduction of Picea abies by artificial layering at the ecotone of the alpine timberline in the Giant (KrkonoŠe) Mountains, Czech Republic | No | Excluded at title/ abstract screening | |
| 897 | 1995 | Valla françois, Sivardière F. | Evolution of avalanche accidents in France from 1971 to 1994. | No | Irrelevant title/ abstract (reports from other methods) | |
| 898 | 2010 | Valt, M. | Avalanche Accidents in the Italian Alps 1985-2009 | No | Excluded at title/ abstract screening | |
| 899 | 2009 | Valt, M.; Chiambretti; Zasso, R. | 1985 - 2009 Twenty-five years of avalanche accidents in Italy | No | No relevant statistics obtained | |
| 900 | 2013 | Valt, Mauro; Pivot, Stefano | Avalanche accident documentation is of fundamental importance to under- stand the dynamics, taking place in snow, of risky activities in order to imple- ment the best possible prevention strategies | No | Irrelevant title/ abstract (reports from other methods) | |
| 901 | 2001 | Van Camp L | Trauma scoring. | | Irrelevant title/ abstract (reports from other methods) | |
| 902 | 2010 | van Herwijnen, Alec; Heierli, Joachim | A FIELD METHOD FOR MEASURING SLAB STIFFNESS AND WEAK LAYER FRACTURE ENERGY | No | Irrelevant title/ abstract (reports from other methods) | |

**Appendix. Data screening record.**

**This table records inclusion and exclusion decisions (with reasons for exclusion) for 1031 studies/registries (750 unique ones from database search and 281 unique ones from reference screening and expert consultation); Reasons to exclude correspond to Fig 1 in the paper**

| Index | Publication Year | Author | Title | If included | Reason for exclusion | URL for grey included paper |
|---|---|---|---|---|---|---|
| 903 | 2017 | Van Tilburg C, Grissom CK, Zafren K, McIntosh S, Radwin MI, Paal P, et al | Wilderness Medical Society practice guidelines for prevention and management of avalanche and nonavalanche snow burial accidents | No | Irrelevant title/ abstract (reports from other methods) | |
| 904 | 2000 | Van Tilburg, C. | In-area and backcountry snowboarding: medical and safety aspects | No | Excluded at title/ abstract screening | |
| 905 | 1996 | Van Tilburg, C. | Backcountry snowboarding: medical and safety aspects | No | Excluded at title/ abstract screening | |
| 906 | 2021 | Van Tilburg, C. | Should Airbag Backpacks Be Standard Avalanche Safety Equipment? | No | Irrelevant title/ abstract (reports from other methods) | |
| 907 | 2003 | Van Tilburg, Christopher | Preventing avalanche and deep snow submersion deaths using an artificial air pocket device and a digital dual-antenna avalanche transceiver | No | Excluded at title/ abstract screening | |
| 908 | 2010 | Van Tilburg, Christopher | Non-avalanche-related snow immersion deaths: tree well and deep snow immersion asphyxiation | No | Excluded at title/ abstract screening | |
| 909 | 2019 | Vargas-Cuervo, Germán; Rotigliano, Edoardo; Conoscenti, Christian | Prediction of debris-avalanches and -flows triggered by a tropical storm by using a stochastic approach: An application to the events occurred in Mocoa (Colombia) on 1 April 2017 | No | Excluded at title/ abstract screening | |
| 910 | 2016 | Vargyas, George | Backcountry Skiers, Avalanche Trauma Mortality, and Helmet Use | No | No relevant statistics obtained | |
| 911 | 2021 | Varsa, P. M.; Baranoski, G. V. G. | In silico assessment of light penetration into snow: Implications to the prediction of slab failures leading to avalanches | No | Excluded at title/ abstract screening | |
| 912 | 2009 | Veulliet, E.; Weck-Hannemann, H.; Stötter, J. | Pros and cons of four years experience of alps | No | Excluded at title/ abstract screening | |
| 913 | 2009 | Viglietti, D.; Letey, S.; Motta, R.; Maggioni, M.; Freppaz, M. | Snow and avalanche: The influence of forest on snowpack stability | No | Excluded at title/ abstract screening | |

## Appendix. Data screening record.

**This table records inclusion and exclusion decisions (with reasons for exclusion) for 1031 studies/registries (750 unique ones from database search and 281 unique ones from reference screening and expert consultation); Reasons to exclude correspond to Fig 1 in the paper**

| Index | Publication Year | Author | Title | If included | Reason for exclusion | URL for grey included paper |
|---|---|---|---|---|---|---|
| 914 | 2010 | Viglietti, D.; Letey, S.; Motta, R.; Maggioni, M.; Freppaz, M. | Snow avalanche release in forest ecosystems: A case study in the Aosta Valley Region (NW-Italy) | No | Excluded at title/ abstract screening | |
| 915 | 2013 | Vikhamar-Schuler, D.; Hanssen-Bauer, I.; Schuler, T. V.; Mathiesen, S. D.; Lehning, M. | Use of a multilayer snow model to assess grazing conditions for reindeer | No | Excluded at title/ abstract screening | |
| 916 | 2019 | Vikulina, M. A. | Estimation of avalanche risk in khibiny | No | No relevant statistics obtained | |
| 917 | 2021 | Vilca, Oscar; Mergili, Martin; Emmer, Adam; Frey, Holger; Huggel, Christian | The 2020 glacial lake outburst flood process chain at Lake Salkantaycocha (Cordillera Vilcabamba, Peru) | No | Irrelevant title/ abstract (reports from other methods) | |
| 918 | 1986 | Villar, R. N. | Casualties on Everest-an evacuation problem | No | No relevant statistics obtained | |
| 919 | 2014 | Voiculescu, M. | Patterns of the dynamics of human-triggered snow avalanches at the Făgăras{comma below} massif (Southern Carpathians), Romanian Carpathians | No | Excluded at title/ abstract screening | |
| 920 | 2016 | Voiculescu, M.; Ardelean, F.; Török-Oance, M.; Milian, N. | Topographical factors, meteorological variables and human factors in the control of the main snow avalanche events in the fĂgĂraŞ massif (Southern carpathians – Romanian Carpathians): Case studies | No | Excluded at title/ abstract screening | |
| 921 | 2011 | Voiculescu, M.; Popescu, F. | Management of snow avalanche risk in the Ski areas of the Southern Carpathians-Romanian Carpathians case study: The Bâlea (FăgăraŚ Massif) and Sinaia (Bucegi Mountains) Ski areas | No | Excluded at title/ abstract screening | |
| 922 | 1994 | Vretenar, D. F.; Urschel, J. D.; Parrott, J. C.; Unruh, H. W. | Cardiopulmonary bypass resuscitation for accidental hypothermia | No | Excluded at title/ abstract screening | |

| Appendix. Data screening record. | | | | | | |
|---|---|---|---|---|---|---|
| **This table records inclusion and exclusion decisions (with reasons for exclusion) for 1031 studies/registries (750 unique ones from database search and 281 unique ones from reference screening and expert consultation); Reasons to exclude correspond to Fig 1 in the paper** | | | | | | |
| **Index** | **Publication Year** | **Author** | **Title** | **If included** | **Reason for exclusion** | **URL for grey included paper** |
| 923 | 2014 | Vrtis, George | Encounters in Avalanche Country: A History of Survival in the Mountain West, 1820-1920 | No | No relevant statistics obtained | |
| 924 | 1986 | Vuori I | The cardiovascular risks of physical activity. | No | Irrelevant title/ abstract (reports from other methods) | |
| 925 | 1975 | Waddell G | Mountain rescue transport. | No | Irrelevant title/ abstract (reports from other methods) | |
| 926 | 2009 | Wagner, Eric | The Fall Guy | No | Excluded at title/ abstract screening | |
| 927 | 2012 | Wagner, Wendy | Investigating the Snow Climate of Turnagain Pass, Alaska | No | Excluded at title/ abstract screening | |
| 928 | 2019 | Walcher, Matthias; Haegeli, Pascal; Fuchs, Sven | Risk of Death and Major Injury from Natural Winter Hazards in Helicopter and Snowcat Skiing in Canada | No | No relevant statistics obtained | |
| 929 | 2014 | Walker, Emma; Latosuo, Eeva | Decision-Making Practices in Alaska's Dynamic Mountain Environments: A Study of Professional Mountain Guides | No | Excluded at title/ abstract screening | |
| 930 | 2019 | Wallner, B.; Moroder, L.; Brandt, A.; Mair, P.; Erhart, S.; Bachler, M.; Putzer, G.; Turner, R.; Strapazzon, G.; Falk, M.; Brugger, H. | Extrication Times During Avalanche Companion Rescue: A Randomized Single-Blinded Manikin Study | No | Excluded at title/ abstract screening | |
| 931 | 2004 | Walsh, S. J.; Weiss, D. J.; Butler, D. R.; Malanson, G. P. | An assessment of snow avalanche paths and forest dynamics using Ikonos satellite data | No | Excluded at title/ abstract screening | |
| 932 | 2020 | Walter, Fabian; Amann, Florian; Kos, Andrew; Kenner, Robert; Phillips, Marcia; de Preux, Antoine; Huss, Matthias; Tognacca, Christian; Clinton, John; Diehl, Tobias; Bonanomi, Yves | Direct observations of a three million cubic meter rock-slope collapse with almost immediate initiation of ensuing debris flows | No | Excluded at title/ abstract screening | |
| 933 | 2020 | Wang, Xiuling; Lai, Jinxing; Qiu, Junling; Xu, Wei; Wang, Lixin; Luo, Yanbin | Geohazards, reflection and challenges in Mountain tunnel construction of China: a data collection from 2002 to 2018 | No | Excluded at title/ abstract screening | |

| Appendix. Data screening record. | | | | | | |
|---|---|---|---|---|---|---|
| This table records inclusion and exclusion decisions (with reasons for exclusion) for 1031 studies/registries (750 unique ones from database search and 281 unique ones from reference screening and expert consultation); Reasons to exclude correspond to Fig 1 in the paper | | | | | | |
| **Index** | **Publication Year** | **Author** | **Title** | **If included** | **Reason for exclusion** | **URL for grey included paper** |
| 934 | 2011 | Wang, Z.; Qi, L.; Yu, G.; Liu, C. | New challenges in erosion and sedimentation research: A Chinese perspective | No | Excluded at title/abstract screening | |
| 935 | 2011 | Wang, Zhaoyin; Shi, Wenjing; Liu, Dandan | Continual erosion of bare rocks after the Wenchuan earthquake and control strategies | No | Excluded at title/abstract screening | |
| 936 | 1980 | Ward, R. G. W. | Avalanche hazard in the Cairngorm Mountains, Scotland | No | No relevant statistics obtained | |
| 937 | 1984 | Ward, R. G. W. | Avalanche prediction in Scotland: I. A survey of avalanche activity | No | Excluded at title/abstract screening | |
| 938 | 2021 | Wasowski, J.; McSaveney, M. J.; Pisano, L.; Del Gaudio, V.; Li, Y.; Hu, W. | Recurrent rock avalanches progressively dismantle a mountain ridge in Beichuan County, Sichuan, most recently in the 2008 Wenchuan earthquake | No | Excluded at title/abstract screening | |
| 939 | 1999 | Watzman, Nancy | Snow business | No | Excluded at title/abstract screening | |
| 940 | 2014 | Weber-Endress, S.; Endress, T. | Accidental falls into glacial crevasses in winter. Evidence-based algorithm for accident prevention and companion rescue from a medical perspective | No | Irrelevant title/abstract (reports from other methods) | |
| 941 | 2000 | Weber, Holly A.; Wilderness Medicine Newsletter, Inc Conway N. H. | Wilderness Medicine Newsletter, 2000: For the Recognition, Treatment, and Prevention of Wilderness Emergencies | No | Excluded at title/abstract screening | |
| 942 | 1998 | Weber, Holly A.; Wilderness Medicine Newsletter, Inc Conway N. H. | Wilderness Medicine Newsletter, Volume 9 | No | Excluded at title/abstract screening | |
| 943 | 2014 | Werner, A.; Tiedemann, J.; Gunga, H. C.; Falk, M.; Brugger, H.; Paal, P. | Measurement of body core temperature with heat flux double sensor in hypothermic pigs during artificial avalanche burial | No | Excluded at title/abstract screening | |
| 944 | 2010 | Werner, A.; Tiedemann, J.; Gunga, H. C.; Falk, M.; Brugger, H.; Paal, P. | Measurement of body core temperature by heat flux double sensor in hypothermic pigs during artificial avalanche burial | No | Excluded at title/abstract screening | |

| Appendix. Data screening record. | | | | | | |
|---|---|---|---|---|---|---|
| **This table records inclusion and exclusion decisions (with reasons for exclusion) for 1031 studies/registries (750 unique ones from database search and 281 unique ones from reference screening and expert consultation); Reasons to exclude correspond to Fig 1 in the paper** | | | | | | |
| **Index** | **Publication Year** | **Author** | **Title** | **If included** | **Reason for exclusion** | **URL for grey included paper** |
| **945** | **2013** | Westensee J, Rogé I, Van Roo JD, et al. | Mountaineering fatalities on Aconcagua: 2001-2012. | No | Irrelevant title/ abstract (reports from other methods) | |
| **946** | **2012** | Westhoff, John L.; Koepsell, Thomas D.; Littell, Christopher T. | Effects of experience and commercialisation on survival in Himalayan mountaineering: retrospective cohort study | No | Excluded at title/ abstract screening | |
| **947** | **1977** | Weston, J. T.; Moore, S. M.; Rich, T. H. | A five year study of mortality in a busy ski population | No | No relevant statistics obtained | |
| **948** | **2000** | Whiteman CD | Mountain Meteorology: Fundamentals and Applications | No | Irrelevant title/ abstract (reports from other methods) | |
| **949** | **1999** | Whitney, Craig R. | After the Avalanches, Europe Skis Into Spring | No | Excluded at title/ abstract screening | |
| **950** | **2009** | Wick MC, Weiss RJ, Hohlrieder M, Tecklenburg K, Jaschke W, Rieger M | Radiological aspects of injuries of avalanche victims | No | Irrelevant title/ abstract (reports from other methods) | |
| **951** | **2022** | Wik, L.; Brattebo, G.; Osteras, O.; Assmus, J.; Irusta, U.; Aramendi, E.; Mydske, S.; Skaalhegg, T.; Skaiaa, S. C.; Thomassen, O. | Physiological effects of providing supplemental air for avalanche victims. A randomised trial | No | Excluded at title/ abstract screening | |
| **952** | **2008** | Wild, Finlay J. | Epidemiology of mountain search and rescue operations in Banff, Yoho, and Kootenay National Parks, 2003-06 | No | No relevant statistics obtained | |
| **953** | **2001** | Wilkinson, Todd | Seeking backcountry thrills, skiers find avalanches | No | Excluded at title/ abstract screening | |
| **954** | **2007** | Williams R, Delaney T, Nelson E, et al. | Speeds associated with skiing and snowboarding. | No | Irrelevant title/ abstract (reports from other methods) | |
| **955** | **2023** | Winder, Samantha G.; Lia, Emilia H.; Braun, Laura A.; Guard, Charlotte; Schell, Scott; Wood, Spencer A. | MONITORING WINTER BACKCOUNTRY RECREATION | No | Excluded at title/ abstract screening | |
| **956** | **2020** | Windsor, J. | Mountain deaths | No | No relevant statistics obtained | |
| **957** | **2009** | Windsor, J. S. | Mountain mortality: a review of deaths that occur during recreational activities in the mountains | No | No relevant statistics obtained | |
| **958** | **2002** | Wines, Michael | Rising Star Lost in Russia's Latest Disaster | No | Excluded at title/ abstract screening | |

| Appendix. Data screening record. | | | | | | |
|---|---|---|---|---|---|---|
| **This table records inclusion and exclusion decisions (with reasons for exclusion) for 1031 studies/registries (750 unique ones from database search and 281 unique ones from reference screening and expert consultation); Reasons to exclude correspond to Fig 1 in the paper** | | | | | | |
| **Index** | **Publication Year** | **Author** | **Title** | **If included** | **Reason for exclusion** | **URL for grey included paper** |
| **959** | **2015** | Winter, M. B.; Baier, R.; Ammer, C. | Regeneration dynamics and resilience of unmanaged mountain forests in the Northern Limestone Alps following bark beetle-induced spruce dieback | No | Irrelevant title/ abstract (reports from other methods) | |
| **960** | **2014** | Wise MP, Horn J, Aneman A, Nilsen N. | Targeted temperature management after out-of-hospital cardiac arrest: certainties and uncertainties. | No | Irrelevant title/ abstract (reports from other methods) | |
| **961** | **2008** | Wise, Jeff | AVALANCHE! | No | Excluded at title/ abstract screening | |
| **962** | **2021** | Witting, Maximilian; Filimon, Sascha; Kevork, Sevag | Carry along or not? Decision-making on carrying standard avalanche safety gear among ski tourers in a German touring region | No | Excluded at title/ abstract screening | |
| **963** | **2016** | Witze, Alexandra | WHITE OUT. (Cover story) | No | Excluded at title/ abstract screening | |
| **964** | **2013** | Wolcke B | Lay-rescuer CPR – controversies in emergency medicine: lay rescuer CPR with or without mouth-to-mouth ventilation. | No | Irrelevant title/ abstract (reports from other methods) | |
| **965** | **2015** | Wolfe, V.; Frobe, W.; Shrinivasan, V.; Hsieh, T. Y. | Detecting and locating cell phone signals from avalanche victims using unmanned aerial vehicles | No | Excluded at title/ abstract screening | |
| **966** | **1987** | Wolkomir, Richard; Wolkomir, Joyce | When Adventure is a Snow Job | No | Excluded at title/ abstract screening | |
| **967** | **1991** | Wood SC | Interactions between hypoxia and hypothermia. | No | Irrelevant title/ abstract (reports from other methods) | |
| **968** | **1998** | Wood, Chris | A Trudeau tragedy | No | Excluded at title/ abstract screening | |
| **969** | **2019** | Wood, Joanne; Bennie, Jonathan | Do geomorphological processes constrain vegetation zones in mountain regions? | No | Excluded at title/ abstract screening | |
| **970** | **2022** | Wright, B. R. | Evidence that predator satiation drives reproductive synchrony in the desert masting grass, soft spinifex (Triodia pungens) | No | Excluded at title/ abstract screening | |
| **971** | **2003** | Xiang H, Stallones L | Deaths associated with snow skiing in Colorado 1980-81 to 2002–2001 ski seasons. | No | Irrelevant title/ abstract (reports from other methods) | |

| Appendix. Data screening record. | | | | | | |
|---|---|---|---|---|---|---|
| **This table records inclusion and exclusion decisions (with reasons for exclusion) for 1031 studies/registries (750 unique ones from database search and 281 unique ones from reference screening and expert consultation); Reasons to exclude correspond to Fig 1 in the paper** | | | | | | |
| **Index** | **Publication Year** | **Author** | **Title** | **If included** | **Reason for exclusion** | **URL for grey included paper** |
| 972 | 2004 | Xiang H, Stallones L, Smith GA. | Downhill skiing injury fatalities among children | No | Irrelevant title/ abstract (reports from other methods) | |
| 973 | 2008 | Xie, H.; Deng, J.; Tai, J.; He, C.; Wei, J.; Chen, J.; Li, X. | Wenchuan large earthquake and post-earthquake reconstruction-related geotechnical problems | No | Irrelevant title/ abstract (reports from other methods) | |
| 974 | 2012 | Xu, M.; Wang, Z.; Qi, L.; Liu, L.; Zhang, K. | Disaster chains initiated by the Wenchuan earthquake | No | Excluded at title/ abstract screening | |
| 975 | 2020 | Yang, Jinming; Li, Chengzhi; Li, Lanhai; Ding, Jianli; Zhang, Run; Han, Tao; Liu, Yang | Automatic Detection of Regional Snow Avalanches with Scattering and Interference of C-band SAR Data | No | Excluded at title/ abstract screening | |
| 976 | 2011 | Yasunaga H, Miyata H, Horiguchi H, et al. | Population density, callresponse interval, and survival of out-of-hospital cardiac arrest. | No | Irrelevant title/ abstract (reports from other methods) | |
| 977 | 2006 | Yilmaz, I.; Ekemen, T.; Yildirim, M.; Keskin, I.; Özdemir, G. | Failure and flow development of a collapse induced complex landslide: The 2005 Kuzulu (Koyulhisar, Turkey) landslide hazard | No | Irrelevant title/ abstract (reports from other methods) | |
| 978 | 2020 | Zachau | New Zealand coronial data analysis of avalanche fatalities | Yes, only for systematic review | NA, since included | https://drmwildernessemc.files.wordpress.com/2019/04/new-zealand-coronial-data-analysis-of-avalanche-fatalities-200.pdf. |
| 979 | 2004 | Zar JH. | Biostatistical analysis. 5th ed | No | Irrelevant title/ abstract (reports from other methods) | |
| 980 | 2021 | Zeitler, C.; Humenberger, M. | Injuries in Skiing and Snowboard tours | No | No relevant statistics obtained | |
| 981 | 2015 | Zhang, G. W.; Lei, J. S. | Focal mechanism solutions of moderate-sized aftershocks of the 2015 MS8.1 Nepal earthquake | No | Excluded at title/ abstract screening | |
| 982 | 2022 | Zhang, T. G.; Wang, W. C.; Gao, T. G.; An, B. S.; Yao, T. D. | An integrative method for identifying potentially dangerous glacial lakes in the Himalayas | No | Excluded at title/ abstract screening | |

## Appendix. Data screening record.

**This table records inclusion and exclusion decisions (with reasons for exclusion) for 1031 studies/registries (750 unique ones from database search and 281 unique ones from reference screening and expert consultation); Reasons to exclude correspond to Fig 1 in the paper**

| Index | Publication Year | Author | Title | If included | Reason for exclusion | URL for grey included paper |
|---|---|---|---|---|---|---|
| 983 | 2021 | Zhong, Y. T.; Wei, Y. F.; Min, N. N.; Guan, Q. Y.; Zhao, J.; Zhu, J. Y.; Hu, H. Y.; Geng, R.; Hong, C. Y.; Ji, Y. S.; Li, J.; Zheng, Y. Q.; Zhang, Y. J.; Li, X. R. | Comparative healing of swine skin following incisions with different surgical devices | No | Excluded at title/ abstract screening | |
| 984 | 2015 | Zhou, C.; Song, Y. | Influence of different longitudinal dune positions in the Gurbantunggut Desert on the reproduction of Haloxylon Ammodendron seedlings | No | Excluded at title/ abstract screening | |
| 985 | 2011 | Zhou, Q.; Zhang, C.; Chen, X. | Asymmetric disaster distribution and its cause analysis of the M s8.0 Whenchuan earthquake | No | Excluded at title/ abstract screening | |
| 986 | 1998 | Zimmermann, Tim | Everest? No Problem. Except for This Damn Full-Body Cast | No | Excluded at title/ abstract screening | |
| 987 | 2002 | Zingari, P. C.; Fiebiger, G. | Mountain risks and hazards | No | No relevant statistics obtained | |
| 988 | 1987 | Zink, Roman A.; Glaeser, Heribert | INCIDENCE, MORBIDITY, AND MORTALITY OF TORSO TRAUMA FROM SKIING | No | Excluded at title/ abstract screening | |
| 989 | 2005 | Zischg, A.; Fuchs, S.; Keiler, M.; Stötter, J. | Temporal variability of damage potential on roads as a conceptual contribution towards a short-term avalanche risk simulation | No | Excluded at title/ abstract screening | |
| 990 | 2004 | Zischg, A.; Fuchs, S.; Stotter, J. | Uncertainties and fuzziness in analysing risk related to natural hazards: a case study in the Ortles Alps, South Tyrol, Italy | No | Irrelevant title/ abstract (reports from other methods) | |
| 991 | 1997 | Zobel, D. B.; Antos, J. A. | A decade of recovery of understory vegetation buried by volcanic tephra from Mount St. Helens | No | Excluded at title/ abstract screening | |
| 992 | 2016 | Zorthian, Julia | Lithuanian Climbers Killed in Avalanche on French Alps | No | Excluded at title/ abstract screening | |
| 993 | 2014 | Zurbriggen, N.; Nabel, J. E. M. S.; Teich, M.; Bebi, P.; Lischke, H. | Explicit avalanche-forest feedback simulations improve the performance of a coupled avalanche-forest model | No | Excluded at title/ abstract screening | |

| Appendix. Data screening record. | | | | | | |
|---|---|---|---|---|---|---|
| **This table records inclusion and exclusion decisions (with reasons for exclusion) for 1031 studies/registries (750 unique ones from database search and 281 unique ones from reference screening and expert consultation); Reasons to exclude correspond to Fig 1 in the paper** | | | | | | |
| **Index** | **Publication Year** | **Author** | **Title** | **If included** | **Reason for exclusion** | **URL for grey included paper** |
| 994 | 2011 | Zurick, D. | Post-tsunami recovery in South Thailand, with special reference to the tourism industry | No | Excluded at title/ abstract screening | |
| 995 | 2012 | Zweifel B, Techel F, Björk C. | Who is involved in avalanche accidents? | No | Irrelevant title/ abstract (reports from other methods) | |
| 996 | 2014 | Zweifel, B.; Haegeli, P. | A qualitative analysis of group formation, leadership and decision making in recreation groups traveling in avalanche terrain | | Excluded at title/ abstract screening | |
| 997 | 2016 | Zweifel, Benjamin; Procter, Emily; Techel, Frank; Strapazzon, Giacomo; Boutellier, Roman | Risk of Avalanche Involvement in Winter Backcountry Recreation: The Advantage of Small Groups | No | No relevant statistics obtained | |
| 998 | 2012 | Zweifel, Benjamin; Techel, Frank; BjÃ¶rk, Christian | Who Is Involved In Avalanche Accidents? | No | Excluded at title/ abstract screening | |
| 999 | 2021 | Бабурин, В Л; Бадин, С В | ПРОГНОЗИРОВАНИЕ СОЦИАЛЬНО-ЭКОНОМИЧЕСКИХ УЩЕРБОВОТ ОПАСНЫХ ПРИРОДНЫХ ПРОЦЕССОВ ДЛЯ ТУРИСТИЧЕСКОГОКЛАСТЕРА «КУРОРТЫ СЕВЕРНОГО КАВКАЗА» | No | Excluded at title/ abstract screening | |
| 1000 | 1993 | | Avalanche claims three climbers | No | Excluded at title/ abstract screening | |
| 1001 | 1996 | | White death | No | Excluded at title/ abstract screening | |
| 1002 | 1996 | | 2 Americans die in Nepal | No | Excluded at title/ abstract screening | |
| 1003 | 1994 | | Vital moments after an avalanche | No | Excluded at title/ abstract screening | |
| 1004 | 1998 | | Man dies as climbers on Mount Rainier are hit by avalanche | No | Excluded at title/ abstract screening | |
| 1005 | 2012 | | Corrections of misinformation about avalanche | No | Excluded at title/ abstract screening | |
| 1006 | 2002 | | Five Student Climbers Die in Avalanche | No | Excluded at title/ abstract screening | |
| 1007 | 2002 | | Avalanche and gears | No | Excluded at title/ abstract screening | |
| 1008 | 2010 | | Austria: Experiment On Pigs Suspended | No | Excluded at title/ abstract screening | |
| 1009 | 10 | | Paid Notice: Deaths WALD, STEPHEN L | No | Excluded at title/ abstract screening | |
| 1010 | 2009 | | Avalanche disasters | No | Excluded at title/ abstract screening | |

| **Appendix. Data screening record.** | | | | | | |
|---|---|---|---|---|---|---|
| **This table records inclusion and exclusion decisions (with reasons for exclusion) for 1031 studies/registries (750 unique ones from database search and 281 unique ones from reference screening and expert consultation); Reasons to exclude correspond to Fig 1 in the paper** | | | | | | |
| **Index** | **Publication Year** | **Author** | **Title** | **If included** | **Reason for exclusion** | **URL for grey included paper** |
| **1011** | **2008** | | K2 and Its Consequences | No | Excluded at title/ abstract screening | |
| **1012** | **2008** | | Lesley toughs it out on Gasherbrum I | No | Excluded at title/ abstract screening | |
| **1013** | **2000** | | Swiss Avalanche Kills Skier | No | Excluded at title/ abstract screening | |
| **1014** | **1999** | | People in the News | No | Excluded at title/ abstract screening | |
| **1015** | **2006** | | Alpine Ski Resorts Are on Alert | No | Excluded at title/ abstract screening | |
| **1016** | **2005** | | CONRAD ANKER: HIGH-ALTITUDE ALTRUIST | No | Excluded at title/ abstract screening | |
| **1017** | **1998** | | World notes | No | Excluded at title/ abstract screening | |
| **1018** | **2005** | | Survival Facts | No | Excluded at title/ abstract screening | |
| **1019** | **2005** | | KASHMIR AVALANCHE | No | Excluded at title/ abstract screening | |
| **1020** | **2005** | | AID TO SNOWBOUND VILLAGES | No | Excluded at title/ abstract screening | |
| **1021** | **1999** | | Avalanches Kill 5 In the French Alps | No | Excluded at title/ abstract screening | |
| **1022** | **2020** | | Dragons in the Snow: Avalanche Detectives and the Race to Beat Death in the Mountains | No | Excluded at title/ abstract screening | |
| **1023** | **2004** | | World Briefing | No | Excluded at title/ abstract screening | |
| **1024** | **2004** | | BREATHING DEVICE SAVES MAN CAUGHT IN AVALANCHE | No | Excluded at title/ abstract screening | |
| **1025** | **2016** | | Imaging Findings of a Survivor of Avalanche without Any Life Support at Very High Altitude and Extreme Low Temperatures | No | Excluded at title/ abstract screening | |
| **1026** | **2015** | | China's Man-Made Disasters | No | Excluded at title/ abstract screening | |
| **1027** | **2015** | | Two U. S. Skiers Die in Austria In Avalanche | No | Excluded at title/ abstract screening | |
| **1028** | **2017** | | Expedition Organizers Supply Mount Everest Climbers with Trash Bags to Collect Waste | No | Excluded at title/ abstract screening | |
| **1029** | **2004** | | DEATHS | No | Excluded at title/ abstract screening | |
| **1030** | **2022** | | Book Review. Snow Avalanches – Beliefs, Facts and Science | No | Irrelevant title/ abstract (reports from other methods) | |
| **1031** | | | | No | Irrelevant title/ abstract (reports from other methods) | |

| Appendix. Data screening record. | | | | | | |
|---|---|---|---|---|---|---|
| **This table records inclusion and exclusion decisions (with reasons for exclusion) for 1031 studies/registries (750 unique ones from database search and 281 unique ones from reference screening and expert consultation); Reasons to exclude correspond to Fig 1 in the paper** | | | | | | |
| **Index** | **Publication Year** | **Author** | **Title** | **If included** | **Reason for exclusion** | **URL for grey included paper** |
| | 2002 | | Field tests of some new avalanche rescue devices. | No | Irrelevant title/ abstract (reports from other methods) | |
| | | | | | | |
