# Supplementary material for: Death of backcountry winter-sports practitioners in avalanches – A systematic review and meta-analysis of proportion of causes of avalanche death
Source: PLOS Glob Public Health. 2025 May 30;5(5):e0004551. doi: 10.1371/journal.pgph.0004551 (PMC12124587; doi:10.1371/journal.pgph.0004551)
Supplement: S5 Table — (PDF) [file pgph.0004551.s007.pdf]

Name of the first author	Year	Data extractor (Verifier)	Date of extraction	Eligibility confirmation	sample_size	trauma	asphyxia	hypothemia	asphyxia_hypothermia	asphyxia_trauma	drowning	representativeness	location	forensic	time_span
Alnoncourt	2017	RG(LA)	May 15th, 2024	Eligible for our MA and SR	25	7	24	0	0	0	0	Local	France	Full autopsy	Post-2000
Bilek and Würtl	2011	RG(LA)	May 25th, 2024	Eligible for our MA and SR	131	46	NA	2	0	0	0	Unclear	Austria	Mixed	Post-2000
Blancher	2017	RG(LA)	May 26th, 2024	Eligible for our SR-only	5	0	5	0	0	0	0	SR-only study, NA			
Boyd	2009	RG(LA)	May 15th, 2024	Eligible for our MA and SR	204	48	154	2	0	0	0	National	Canada	Mixed	Across 2000
Christensen	1999	RG(LA)	May 15th, 2024	Eligible for our MA and SR	12	2	10	0	0	0	0	Local	USA	Strategic	1970-2000
Degawa	2023	RG(LA)	May 25th, 2024	Eligible for our MA and SR	163	29	NA	8	0	0	0	National	Japan	Unclear	Across 2000
Eidenbenz	2021	RG(LA)	May 26th, 2024	Eligible for our SR-only	67(94, n= 113)	9	41	9	0	0	0	SR-only study, NA			
Eliakis	1974	RG(LA)	May 15th, 2024	Eligible for our MA and SR	24	1	21	2	0	0	0	Local	Switzerland	Unclear	NA
Fredriksen	2013	RG(LA)	May 15th, 2024	Eligible for our MA and SR	39	8	27	2	0	0	0	Local	Norway	Mixed	Across 2000
Gross	2021	RG(LA)	May 15th, 2024	Eligible for our MA and SR	32	NA	22	NA	0	0	0	Hospital	Switzerland	Mixed	Post-2000
Grosse	2007	RG(LA)	May 26th, 2024	Eligible for our SR-only	2	0	2	2	0	0	0	SR-only study, NA			
Grossman	1989 (C 1)	RG(LA)	May 15th, 2024	Eligible for our MA and SR	12	2	10	0	0	0	0	Local	USA	Mixed	1970-2000
Grossman	1989 (C 2)	RG(LA)	May 15th, 2024	Eligible for our MA and SR	390	97	273	8	0	0	0	Multi-National	Multiple european nations	Unclear	1970-2000
Grossman	1989 (C 3)	RG(LA)	May 15th, 2024	Eligible for our MA and SR	45	12	28	0	0	0	0	National	Canada	Unclear	1970-2000
Haegeli	2011	RG(LA)	May 26th, 2024	Eligible for our SR-only	143	27	116	0	0	0	0	SR-only study, NA			
Hatwal	2021	RG(LA)	May 26th, 2024	Eligible for our SR-only	12	12	0	0	0	0	0	SR-only study, NA			
Hohlrieder	2007	RG(LA)	May 26th, 2024	Eligible for our MA and SR	36	2	33	1	0	0	0	Local	Austria	Strategic	Across 2000
Irwin	2002	RG(LA)	April 28th, 2024	Eligible for our MA and SR	19	2	16	3	0	0	0	Local	Newzealand	Unclear	1970-2000
Jamieson	2007	RG(LA)	April 28th, 2024	Eligible for our MA and SR	10	2	8	0	0	0	0	National	Canada	Unclear	Post-2000
Johnson	2001	RG(LA)	May 15th, 2024	Eligible for our MA and SR	28	6	22	NA	0	0	0	Local	USA	Unclear	1970-2000
Lapras	1980	RG(LA)	May 26th, 2024	Eligible for our SR-only	40	11	17	2	0	0	0	SR-only study, NA			
Locher	1996	RG(LA)	May 26th, 2024	Eligible for our SR-only	16	0	8	8	0	0	0	SR-only study, NA			
Lugger and Unterdorfer	1972	RG(LA)	May 15th, 2024	Eligible for our MA and SR	20	1	19	0	0	0	0	Local	Austria	Full autopsy	Pre-1970
Mair	2012	RG(LA)	May 26th, 2024	Eligible for our SR-only	NA	NA	NA	NA	NA	NA	NA	SR-only study, NA			
Markwalder	1970	RG(LA)	May 26th, 2024	Eligible for our SR-only	29	7	23	1	0	0	0	SR-only study, NA			
Martínez	2022	RG(LA)	May 15th, 2024	Eligible for our MA and SR	42	14	24	4	0	0	0	Local	Spain	Mixed	Across 2000
McIntosh	2007	RG(LA)	May 15th, 2024	Eligible for our MA and SR	56	8	53	0	0	5	0	Local	USA	Mixed	Across 2000
McIntosh	2019	RG(LA)	May 15th, 2024	Eligible for our MA and SR	32	9	26	0	0	3	0	Local	USA	Mixed	Post-2000
Moroder	2015	RG(LA)	May 15th, 2024	Eligible for our MA and SR	13	3	10	0	0	0	0	Local	Austria	Full autopsy	Post-2000
Oshiro	2022	RG(LA)	May 15th, 2024	Eligible for our MA and SR	26	4	22	0	0	0	0	Local	Japan	External autopsy	Post-2000
Pasquier	2017	RG(LA)	May 26th, 2024	Eligible for our SR-only	35	33	2	0	0	0	0	SR-only study, NA			
Sheets	2018	RG(LA)	May 15th, 2024	Eligible for our MA and SR	110	32	74	4	0	0	0	Local	USA	Mixed	Across 2000

Name of the first author	Year	Data extractor (Verifier)	Date of extraction	Eligibility confirmation	sample_size	trauma	asphyxia	hypothemia	asphyxia_hypothermia	asphyxia_trauma	drowning	representativeness	location	forensic	time_span
Stalsberg	1989	RG(LA)	May 15th, 2024	Eligible for our MA and SR	18	2	16	0	0	0	0	National	Norway	Mixed	1970-2000
Tough	1993	RG(LA)	May 15th, 2024	Eligible for our MA and SR	19	3	15	1	0	0	0	Local	Canada	Full autopsy	1970-2000
Zachau	2020	RG(LA)	May 26th, 2024	Eligible for our SR-only	21	9	11	1	0	0	0	SR-only study, NA			
Techel and Zweifel	2013	RG(LA)	May 26th, 2024	Eligible for our SR-only	276	115	152	8	0	0	0	SR-only study, NA			
Tanka	2024	RG(LA)	May 26th, 2024	Eligible for our SR-only	5	NA	NA	NA	0	0	0	SR-only study, NA			
Geisenberger	2015	RG(LA)	May 26th, 2024	Eligible for our SR-only	0	0	0	0	2	0	0	SR-only study, NA			
Kobek	2003	RG(LA)	May 26th, 2024	Eligible for our SR-only	6	0	6	0	0	0	0	SR-only study, NA			
