# Supplementary material for: Death of backcountry winter-sports practitioners in avalanches – A systematic review and meta-analysis of proportion of causes of avalanche death
Source: PLOS Glob Public Health. 2025 May 30;5(5):e0004551. doi: 10.1371/journal.pgph.0004551 (PMC12124587; doi:10.1371/journal.pgph.0004551)
Supplement: S6 Table — (PDF) [file pgph.0004551.s012.pdf]

Publication	1. Was the study's target population a close representation of the national population in relation to relevant variables, e.g. age, sex, occupation?<br>(Nationally representative of avalanche death or above got a yes; sport practitioners' avalanche death got a no)	2. Was the sampling frame a true or close representation of the target population?<br><br>(Does the hospital represent the local?)	3. Was some form of random selection used to select the sample, OR, was a census undertaken?<br><br>(If all cases in sampling frame were included, yes.)	4. Was the likelihood of non-response bias minimal?	5. Were data collected directly from the subjects (as opposed to a proxy)?<br><br>(Researcher determined the cause of death by reading the records, getting a no)	6. Was an acceptable case definition used in the study?	7. Was the study instrument that measured the parameter of interest (e.g. prevalence of low back pain) shown to have reliability and validity (if necessary)?<br><br>(A categorization including the three or more got a yes)	8. Was the same mode of data collection used for all subjects?<br><br>(All autopsy or some autopsy according to medical consideration got a yes)	9. Was the length of the shortest prevalence period for the parameter of interest appropriate?<br><br>(A period of at least 1 year got a yes)	10. Were the numerator(s) and denominato r(s) for the parameter of interest appropriate?<br><br>(If there is some unkonwn causes that is not due to random missing or not clarified as random missing, got a no)	11. Summary item on the overall risk of study bias
Alnoncourt, 2017	No.<br>(All avalanche victims who died and were autopsied at the forensic institute of Grenoble University Hospital)	No.<br>(Single hospital)	No.<br>(Cases who were not autopsied were not included)	Yes	Yes<br>(The medical examiners had direct access to the bodies)	Yes.<br>(Clearly defined)	Yes<br>(Three types of cause including trauma, asphyxia and hypothermia)	Yes<br>(All from autopsy)	Yes.<br>(7 years)	No<br>(Some cases without autopsy were not possible to include in the calculation)	High
Bilek and Würtl, 2011	Yes<br>(Police report, national)	Yes<br>(The police report was reviewed again for all 143 fatalities or, if anything was unclear, again with those involved officials asked)	Yes<br>(All cases included)	Yes	Yes<br>(The district or postmortem doctors have direct access to the body)	No	Yes<br>(Three types of cause including trauma, asphyxia and hypothermia)	No<br>(Not all underwent autopsy)	Yes<br>(6 years)	No<br>(12 unclear causes)	Moderate
Boyd, 2009	Yes<br>(We included all victims who died from snow avalanches, as the primary event, in outdoor terrain.)	Yes.<br>(we investigated all snow avalanche deaths in British Columbia and Alberta between Apr. 1, 1984, and Apr. 5, 2005. This date range coincided with entries of avalanche data in the Canadian Avalanche Centre Avalanche Incident Database that were complete upon study inception.)	Yes.<br>(All cases included)	Yes	Yes<br>(Medical examiners who made the record had direct access to the bodies)	No	Yes<br>(Three types of cause including trauma, asphyxia and hypothermia)	No<br>In 117 cases (57%), an autopsy had been performed. In the remaining 87 cases, a forensic external examination without dissection had been conducted.	Yes<br>(11 years)	Yes<br>All causes were reported	Moderate
Christensen, 1999	No<br>(Only Mountaineers)	Yes<br>(The case files of the Pierce County Medical Examiner's Office were reviewed for all fatalities reported on Mount Rainier)	Yes<br>(All cases included)	Yes	Yes.<br>(County Medical Examiner ho made the record had direct access to the bodies)	No	Yes<br>(Three types of cause including trauma, asphyxia and hypothermia)	No.<br>(Autopsies were performed in 30 of the remaining 37 cases (81%). Those cases not autopsied were certified as to cause of death by the coroner without autopsy. Twenty-one of the 30 autopsies were performed by forensic pathologists working in the Pierce County Medical Examiner's Office, and the remaining 9 autopsies were done by hospital pathologists)	Yes<br>(20 years)	Yes<br>All causes were reported	Moderate
Degawa, 2023	No.<br>(The database used include data from various sources, with uncertain reliability of some)	Yes.<br>(The database includes data from most Japanese prefectures)	Yes<br>(All cases included)	Yes	No<br>(It is unclear since the data were from multiple sources)	No	No<br>(Three types of cause including trauma, suffocation and hypothermia. Suffocation does not completely correspond to asphyxia, which is a more common category)	No<br>(It is unclear if autopsy was done to the fatalities)	Yes<br>(30 years)	No<br>(33, or 17% unclear causes)	High
Eliakis, 1974	No	No	No	Yes	No<br>(The author cited forensic diagnoses performed by another person)	No	Yes<br>(Three types of cause including trauma, asphyxia and hypothermia)	No	No	No	High
Fredriksen 2013	Yes<br>(To represent north Norway)	Yes<br>(We searched reports from the rescue teams and the medical records from institutions that received the avalanche victims to determine the most likely cause of death.)	Yes.<br>(All included)	Yes	Yes<br>(Medical Examiner ho made the record had direct access to the bodies)	Yes<br>(Autopsy details for determining the cause were given)	Yes<br>(Three types of cause including trauma, asphyxia and hypothermia)	No<br>(5 underwent autopsy. Reasons for not having autopsy were unclear)	Yes<br>(16 years)	No<br>(9 unclear causes)	Moderate
Gross, 2021	No<br>Only **victims referred to the emergency unit of a major level I referral trauma centre**	No.<br>(There can be missing cases who died on-site)	Yes<br>(All cases included)	Yes	Yes.<br>(The researchers had access to the bodies)	No	Yes<br>(Three types of cause including trauma, asphyxia and hypothermia)	No<br>(Autopsy was denied in 1 of the latter and no injury-related data could be obtained)	Yes<br>(19 years)	No<br>(Before the authors email back, we do not know the causes for 10 avalanche deaths, except that they are for sure not asphyxial death)	High
Grossman,1989 (cohort 1)	Yes<br>(Utha Representative)	Yes<br>(The United States For. est Service maintains the Utah Avalanche Forecasting Center (U.A.F.C.) in Salt Lake City run by one of the authors (B.T.). This center collects information on local snowpack conditions, issues daily reports on avalanche hazards, and compiles data on avalanche accidents. Reords from that facility were reviewed for the period January 1, 1982 through December 31, 1987 to identify all avalanches that resulted in major injury or death and to identify victims.)	Yes<br>(All cases included)	Yes	Yes<br>(Medical examiner had access to all bodies)	No	Yes<br>(Three types of cause including trauma, asphyxia and hypothermia)	No<br>(Autopsy data were available in only one case:)	Yes<br>(5 years)	Yes<br>(No unclear cause)	Moderate
Grossman,1989 (cohort 2)	Yes<br>(Multiple European countries)	Yes<br>(International commission on Alpine Rescue collected the data)	Yes<br>(All cases included)	Yes	Yes<br>(Medical examiner had access to all bodies)	No	Yes<br>(Three types of cause including trauma, asphyxia and hypothermia)	No<br>(Unclear if all took autopsy)	Yes<br>(10 years)	Yes<br>(No unclear cause)	Moderate
Grossman,1989 (cohort 3)	Yes	Yes<br>(Canadian Avalanche Association)	Yes<br>(All cases included)	Yes	Yes<br>(Medical examiner had access to all bodies)	No	No<br>(Not clear if hypothermia is considered)	No<br>(Unclear if all took autopsy)	Yes<br>(6 years)	Yes<br>(No unclear cause)	Moderate
Hohlrieder, 2007	Yes<br>(Canada)	Yes<br>(Cited: "Any data relating to the circumstances of the accident were also collected from the accident reports of the mountain rescue service and the mountain police squad.")<br><br>(Comment: We did not regard there is much chance missing any cases)	Yes<br>(Comment: All death cases were included)	Yes	Yes.<br>(Cited: The medical diagnoses established during hospital treatment or the results of the autopsy were obtained for each identified case.)	Yes.<br>(Comment: the definition for injury severity were clarified)<br>(Autopsy details for determining the cause were given)	Yes.<br>(Three types of cause including trauma, asphyxia and hypothermia)	Yes.<br>(Autopsy was performed in 30 of 36 nonsurvivors. The cause of death in the remaining 6 victims was concluded from clinical, radiological, and electrophysiological findings, whereby the presence of major trauma could reliably be excluded in all.)	Yes.<br>(10 years)	Yes.<br>(No unclear cause)	Low
Irwin, 2002	Yes<br>(National)	Yes<br>(National registry)	Yes<br>(All included)	Yes	Yes<br>(Cause of death diagnosis was made by people with access to the body)	No	Yes<br>(Three types of cause including trauma, asphyxia and hypothermia)	No.<br>(Cause of death diagnoses were not reported for two cases. No further information about the reason for the misses)	Yes<br>(16 years)	No<br>(There were cases with unclear causes of death, without further information to evaluate if they are random or biased)	Moderate

Jamieson, 2007	Yes (National)	Yes (National registry)	Yes (All included)	Yes	Yes (Cause of death diagnosis was made by people with access to the body)	No	Yes (Three types of cause including trauma, asphyxia and hypothermia)	No. (Cause of death diagnoses were not reported for two cases. No further information about the reason for the misses)	Yes (The registry includes decades of data. We used the year of 2006-2007 as a supplement to existing studies base the same regions for other years)	No (There were cases with unclear causes of death, without further information to evaluate if they are random or biased)	Moderate
Johnson, 2001	Yes (Utah representative)	Yes (we reviewed the records of the Utah state medical examiner for the period October 1992 through April 1999. While most avalanche victims were pronounced dead at the scene, a few were treated in emergency rooms/hospitals prior to the declaration of death, and in these patients, medical records were also reviewed))	Yes (All cases included)	Yes	Yes (Medical examiner had access to all bodies)	No (Not defined)	No (The cause of death was recorded by the medical examiner as either asphyxiation or blunt trauma. Hypothermia was not considered)	No (Autopsy for the causes of death were not reported)	Yes (7 years)	Yes (No missing causes or data points)	Moderate
Lugger and Unterdorfer, 1972	Yes (Innsbruck representative)	Yes (Avalanche victims who are rescued by the Innsbruck Mountain Rescue Service are sent to the University's Institute of Forensic Medicine to clarify the cause of death.)	Yes (All cases included)	Yes	Yes (Researchers did the autopsies)	Yes	Yes (All three major types of death causes were considered)	Yes (The researchers did all autopises)	Yes (6 years)	Yes (No unclear causes of death)	Low
Martínez， 2022	No (Excluded avalanche death without autopsy)	Yes (Mountain Intervention Unit of the Mossos d'Esquadra; Instituto Cartográfigo y Geológico de Cataluña. (Cartographic and Geological Institute of Catalonia).)	Yes (All cases included)	Yes	Yes (Examiners had direct access to the bodies)	No	Yes (Three types of cause including trauma, asphyxia and hypothermia)	Yes (All autopsy)	Yes 50 years	No (Eight cases without autopsy were not possible to include in the calculation)	Moderate
McIntosh, 2007	Yes (Historical records from the UAC were reviewed for all avalanche fatalities during the winter seasons of 1989–90 to 2005–06.)	Yes (The center investigates all avalanche accidents that occur in Utah by studying the snow pack that contributed to the avalanche as well as reporting on human and safety factors. The UAC is the only organization that performs this service in Utah.)	Yes All cases included	Yes	Yes (Medical examiner had direct access to the bodies)	Yes (Clearly defined in the introduction)	Yes All three types of death including trauma, asphyxia and hypothermia were considered	No. (A medical examiner review and autopsy was conducted in all cases. Fifty percent of the victims received an internal autopsy whereas the other 50% received an external autopsy. No clarification for the reason of not having an internal autopsy)	Yes (15 years)	Yes (No missing causes or data points)	Low
McIntosh, 2019	Yes	Yes	Yes	Yes	Yes	Yes (Author clarified it was an update from a previous study. The category was defined in that study.)	Yes	No	Yes	Yes	Low
Moroder, 2015	No (All avalanche victims to be rescued with out-of-hospital cardiac arrest)	Yes (Data from all rescue missions of the region)	Yes (All included)	Yes	Yes (The researcher had access to the victims)	Yes (Although the authors did not define directly, they can be deduced from the description of autpsy)	Yes All three types of death including trauma, asphyxia and hypothermia were considered	Yes	Yes (Five consecutive winter seasons)	No. (Causes of death were not identified for 3 victims)	Moderate
Oshiro, 2022	Yes (Japan National)	Yes (After each mountain rescue mission, police officers record the event on the rescue report form, which is kept for the annual rescue report for 5 years.)	No (We collected such reports from police headquarters within all prefectures that had over 10 mountain death cases between January 2011 and December 2015 (the last 5 years in which records were kept as of 2016 when this study began)	Yes	Yes (Yes, police officers had direct access to the bodies)	No (Not clarified)	No (Three types of cause including trauma, asphyxia and hypothermia; though hypothermia was not identified but it was considered. However, none autopsy was performed)	Yes (All from external examination)	Yes (5 years)	Yes (No missing causes or data points)	Moderate
Sheets, 2018	Yes. (We reviewed all snow avalanche deaths in the Colorado Avalanche Information Center database for the avalanche years 1994 to 2015.5; We excluded incidents in which avalanche was not a primary contributor to the fatality. We also excluded the 3 avalanche fatalities that resulted from slides off a roof or building, as they did not occur in a wilderness or mountain environment.)	Yes (All Colorado avalanche death over a period of time)	Yes (All cases included)	Yes	Yes (For all cases, the investigator assigned cause of death based on the coroner's official determination of the principal cause of death.)	No Definitions for the causes were not clarified	Yes (Three types of cause including trauma, asphyxia and hypothermia)	No (Mortality data was available for 110 of these (121); 64 had complete forensic autopsies performed.) 64 of 121 (60%) of the subjects in the study had definitive forensic autopsies performed, the remainder had cause of death attributed by external examination only. It is possible that this could result in misclassification in some cases.)	Yes 21 years	No It is possible that information in the missing 11 records would substantially alter our results or our conclusions.	Moderate
Stalsberg, 1989	No. (Two purposive accidents)	No. (No information was given about whether they were all accidents over a period of time in the region)	Yes (All fatality in the accidents were included)	Yes	Yes (Authors did autopsy)	Yes (Although the authors did not define directly, they can be deduced from the description of autopsy)	Yes (4 types of causes, including trauma, asphyxia, drowning and hypothermia). Although no hypothermia was not identified from the victims, it was considered in the forensic diangosing)	No. (Some refused autopsy)	No. (Short time gap between two avalanche accidents)	Yes (Clear number of all cases and number for each causes)	High
Tough, 1993	No. (Local data, only include avalanche death of skiers)	Yes (Ski fatality files from the Alberta Office of the Chief Medical Examiner)	Yes. (All cases included)	Yes	Yes (Ski fatality files from the Alberta Office of the Chief Medical Examiner)	Yes (Authors did not give explicit definition for each cause of death, but it can be summarised from clear case description)	Yes Three types of cause including trauma, asphyxia and hypothermia)	Yes (Pathology and toxicology for most of the cases)	Yes (All cases over 1980~1991 were included)	Yes	Low
