# Supplementary material for: Death of backcountry winter-sports practitioners in avalanches – A systematic review and meta-analysis of proportion of causes of avalanche death
Source: PLOS Glob Public Health. 2025 May 30;5(5):e0004551. doi: 10.1371/journal.pgph.0004551 (PMC12124587; doi:10.1371/journal.pgph.0004551)
Supplement: S7 Table — (PDF) [file pgph.0004551.s013.pdf]

S7 Table. Subgroup analysis for hypothermia

Cause*	Subgroup#	Ns, Nc†	Est(%)‡	Heterogeneity§				$p^{\parallel}$
				PI(%)	Tau^2	p	I^2(%)	
**Time Span**								
Hypothermia	1970-2000	515,7	2(1–7)	0–26	0.72	0.15	36	0.23
	after 2000	235,6	1(0–3)	0–6	0	1	0	
	across 2000	650,7	3(2–6)	1–14	0.28	0.26	22	
**Regions**								
Hypothermia	Austria	200,4	2(0–5)	0–16	0	0.97	0	<0.01
	Canada	278,4	1(0–3)	0–12	0	0.59	0	
	Japan	189,2	4(2–8)	2–8	0	1	0	
	Norway	57,2	4(1–13)	1–13	0	1	0	
	USA	222,5	1(0–72)	0–72	0.651	1	0	
**Data representativeness**								
Hypothermia	Local	485,15	3(1–6)	1–14	0.46	0.98	0	0.79
	National	440,5	1(0–6)	0–33	0.65	0.37	0	
**Forensic diagnostic procedure¶**								
Hypothermia	Mixed	655,9	2(1–4)	0–12	0.47	0.33	13	0.62
	Full or strategic	125,6	2(0–6)	0–11	0	1	0	

* All rows pertain to Hypothermia. This column is kept for aligning with table 3 in the paper

\# Predefined subgroups with <2 cohorts were removed from subgroup analysis. In forensic diagnostic procedure, "Mixed" indicates some cases underwent external examination alone while others had autopsy (or both) for NON-medical reasons; "Full or strategic" indicates either full autopsy or autopsy conducted based on medical considerations.

† Ns: Number of samples; Nc: Number of cohorts.

‡ Subgroup proportion estimates with 95% confidence interval.

§ (a) PI is the 95% prediction interval. (b) p is for Cochran Q test.

‖ Cochran's Q statistic for subgroup differences.
