# Supplementary material for: Death of backcountry winter-sports practitioners in avalanches – A systematic review and meta-analysis of proportion of causes of avalanche death
Source: PLOS Glob Public Health. 2025 May 30;5(5):e0004551. doi: 10.1371/journal.pgph.0004551 (PMC12124587; doi:10.1371/journal.pgph.0004551)
Supplement: Attachment — Submitted filename: Falk_PGPH-D-24-01671.pdf [file pgph.0004551.s014.pdf]

Response to reviewers

February 13, 2025

Response to editor's points

Unless otherwise specified, all page and row references pertain solely to the revised manuscript.

1. Please amend your detailed Financial Disclosure statement. This is published with the article. It must therefore be completed in full sentences and contain the exact wording you wish to be published.

   Response: The texts have been revised in the manuscript. See section 'Financial Disclosure Statement' (P54/R1290). They are presented below for your convenience.

   'The study was supported by a NordForsk project Grappling with Uncertainty in Environments Signaling Spurious Experiential Decisions (GUESSED), Grant Number 105061. Institutional support was provided by the University of Helsinki and UiT The Arctic University of Norway. The funding was received by GR, LA, GP and BUC. Sponsors had no role in study design, data collection and analysis, decision to publish or manuscript preparation. GR and LA received a salary from the project.'

2. Thank you for uploading your study's underlying data set. Unfortunately, the repository you have noted in your Data Availability statement does not qualify as an acceptable data repository according to PLOS's standards.

   At this time, please upload the minimal data set necessary to replicate your study's findings to a stable, public repository (such as figshare or Dryad) and provide us with the relevant URLs, DOIs, or accession numbers that may be used to access these data.

   Response: we have uploaded the data set to replicate findings to Open Science Framework (OSF, https://osf.io/ygza4/) and the link has been clarified in the manuscript (P17/R430).

3. We ask that a manuscript source file is provided at Revision. Please upload your manuscript file as a .doc, .docx, .rtf or .tex.

   Response: a .tex file and its dependencies have been uploaded.

4. Please provide separate figure files in .tif or .eps format. For more information about figure files please see our guidelines.

   Response: Figures .tif format following the guidelines have been uploaded.

5. We noticed that you used "not shown" in the manuscript. We do not allow these references, as the PLOS data access policy requires that all data be either published with the manuscript or made available in a publicly accessible database. Please amend the supplementary material to include the referenced data or remove the references.

   Response: We have provided full results of subgroup analysis in appendix S9 (P54/R1292) and changed the wording about "now shown" in mansucript (P37: Table 3, first line of footnote)

6. We notice that your figures 1 and 2 are included in the manuscript file. Please remove them as there are already uploaded in the File Inventory

   Response: They are removed. Additionally, we split the Fig 2 (a-d) in original manuscript into Fig 2(a-c) and Fig3, for clearer demonstration (captions: P34/R748; P35/R754).

7. As required by our policy on Data Availability, please ensure your manuscript or supplementary information includes the following:

   A numbered table of all studies identified in the literature search, including those that were excluded from the analyses.

   For every excluded study, the table should list the reason(s) for exclusion.

   If any of the included studies are unpublished, include a link (URL) to the primary source or detailed information about how the content can be accessed.

   Response: The numbered table of all identified studies with reasons for exclusion and link to primary source for included grey studies has been created, uploaded to Open Science Framework and attached as Appendix S5–Data screening history (P54/R1285 in manuscript; or P17 in appendix).

   A table of all data extracted from the primary research sources for the systematic review and/or meta-analysis. The table must include the following information for each study:Name of data extractors and date of data extraction. Confirmation that the study was eligible to be included in the review.

All data extracted from each study for the reported systematic review and/or meta-analysis that would be needed to replicate your analyses.

Response: The table of all extracted data has been created, uploaded to , uploaded to Open Science Framework and attached as Appendix S6–Data screening history (P54/R1286 in manuscript; or P18 in appendix).

If data or supporting information were obtained from another source (e.g. correspondence with the author of the original research article), please provide the source of data and dates on which the data/information were obtained by your research group.

Response: Yes, the information is in S4. We did not report dates of contact in our original manuscript but now they are supplemented in appendix S4 (P54/R1284 in manuscript; ; or P15 in appendix).

If applicable for your analysis, a table showing the completed risk of bias and quality/certainty assessments for each study or outcome. Please ensure this is provided for each domain or parameter assessed. For example, if you used the Cochrane risk-of-bias tool for randomized trials, provide answers to each of the signalling questions for each study. If you used GRADE to assess certainty of evidence, provide judgements about each of the quality of evidence factor. This should be provided for each outcome.

Response: We created a table for showing the completed risk of bias assessment for our study. It has been uploaded to Open Science Framework and attached as Appendix S11–Ratings of risk-of-bias assessment (P54/R1291 in manuscript; P23 in appendix).

We believe quality assessments for each of the multiple outcomes are not applicable to our study. There are three causes of avalanche death considered in our study (trauma, asphyxia and hypothermia). However, all three causes arise from a single forensic strategy within each study, and hence any potential bias would affect the diagnosis as a whole, rather than individual outcomes separately. Given this, a separate risk of bias assessment per cause of death would not provide meaningful additional insights. We also clarified this in the manuscript in the section of 'Risk of bias' (P8/R157-160)

An explanation of how missing data were handled.

Response: Methods of handling missing data have been clarified in the section 'Dealing with missing data' (P9/R192).

This information can be included in the main text, supplementary information, or relevant data repository. Please note that providing these underlying data is a requirement for publication in this journal, and if these data are not provided your manuscript might be rejected. Additional Editor Comments (if provided):

Response: Yes, all the information is available now, as described above. This information is essential for enhancing the replicability of our study,

which we have diligently aimed to achieve. Thank you.

Response to reviewers' points

Reviewer 1

1. In the abstract 'PCAD' - Proportions of causes of avalanche death - should be correctly described. It seems that the journal site has not formated the original abstract correctly, see first page of the .pdf file, e.g. 'Between-study heterogeneity was assessed jointly by I^2 and 95% prediction interval of pooled estimates. Meanwhile, we performed a narrative review to supplement the meta-synthesis' and in the conclusions 'A sample size > 75 is needed to estimate the proportion of hypothermia'

   Response: It is now fixed now. See abstract (P1-2)

2. Several references to not correspond to the journal style and relevant data eg journal is missing, see for example ref 3, 10, 11 and many others

   Response: Indeed, there had been problems with migrating the manuscript to PLOS format and thus many of the citations were broken. This is now corrected in the updated manuscript. We have carefully reviewed all references to ensure they adhere to the PLOS journal style. We sincerely apologize for inconvenience caused by the earlier errors and appreciate your feedback in identifying this issue.

Reviewer 2

All page and row references in reviewer comments pertain exclusively to the original manuscript, while those in author responses refer solely to the revised manuscript.

1. Meta-analyses of categorical or ordinal data are typically conducted by treating the data as dichotomous. As a result, the point estimates and confidence intervals apply to individual events, such as trauma, but are not suitable for providing a combined estimate across multiple events. For example, on page 44/926, the proportions of trauma and asphyxia from 1970 to 2000 are reported as 23% and 72%, respectively. For the period after 2000, the proportions are 29% for trauma, 82% for asphyxia, and 2% for hypothermia, totaling 114%. To correct this, the proportions should be normalized so that their sum equals 100%. Applying a normalizing constant of 1/1.14 results in corrected proportions of 25% for trauma, 73% for asphyxia, and 2% for hypothermia, making these values comparable to those before 2000. Since applying a normalizing constant can significantly alter the results, it is essential to use appropriate methods, as suggested in [2, 3, 4, 5], to obtain reliable combined proportion estimates. Alternatively, proportions should at least be normalized when comparing event proportions or subgroups to ensure accurate comparisons

Response: Thank you for your insightful comment regarding the need for a combined estimate across multiple events. We have made changes to relevant places in the manuscript (detailed below). We especially appreciate your suggestion to apply a post-estimate normalization constant or opting for a statistical model enforcing the constraint that the sum of the pooled proportions should be 100%, which we believe to be very good practices in dealing with the issue. However, we found these approaches not applicable to our study due to the nature of the data. We next clarify why we took that decision, and after that what changes we made instead to handle this issue.

Clarification:

a. About adopting a model generating a combined estimate across multiple events

Among the methodological publications you have suggested, we found approach suggest by Trikalinos and Olkin[1] in dealing with mutually exclusive binary outcomes most relevant to our case. The authors concluded that analyzing mutually exclusive outcomes using a multinomial model, at least as a secondary analysis, is a good practice. However, in our study, the included studies vary in whether they report all three causes, two causes, or a combination of multiple causes, meaning it does not fit the assumption on mutual exclusiveness of the outcomes. This is already clarified in Data Extraction (under both Methods and Results sections) and is fully documented in Table 1 (column 'Diagnosis') and described in results (P24/R438) in the original manuscript. The limitations of included studies in using less-than-three-categories, or overlapping-cause classifications, have been accounted for in our risk-of-bias analysis and considered in result interpretation (Fig 3 and Appendix S3) and fully discussed in our original manuscript (P48/R1090).

b. About applying normalization constant

The downside of this approach is that sampling error and heterogeneity are accounted for in the estimates one way or another regardless of the models for meta-analysis adopted (i.e. the summary proportion estimate is influenced by the absolute number of a cause, in addition to the absolute number of sample size), applying normalization after estimation ignores the meta-analytic weights and uncertainty incorporated differently on each cause.

c. About the validity of making proportion estimates separately for each cause (our practice)

In our study, each proportion is estimated separately using GLMM models. We sought to interpret proportion estimate of each cause in its own context and highlighted they should always be interpreted with 95%CI, which quantifies the precision of the estimate. The causes have distinct natural probability and the proportion estimate for each should sensibly have distinct precisions, which inevitably leads to estimates not summing

up to 100%. We believe this does not invalidate our proportion estimates. While admitting in the manuscript we need clearer clarification that this approach yields the proportions not summing up to 100%, we believe separate analysis without imposing artificial trade-offs between causes can account for heterogeneity of each cause more properly (especially when there are different number of studies for each cause). For this reason, our practice better fits one of the principal aims of the study, which is to better understand what factor can explain the high heterogeneity in the overall proportion of estimates. Separately estimating each correlated outcome is also the practice of some high-quality meta-analyzes with a design similar to ours [2; 3].

d. About the comparability of these proportion estimates in subgroup analysis

As suggested by Barker et al.[4], for proportional meta-analyses, differences between subgroups can be compared and contrasted using a statistical test. This practice is also used in other meta-analyzes with a design similar to ours[3]. Since in our study, heterogeneity and uncertainty are modeled independently for each cause of death, differences in pooled estimates between subgroups reflect probability-based changes in absolute prevalence. The focus is then on how each proportion estimate independently changes across subgroups, rather than their relative composition within a fixed total. This indicates that in interpreting the subgroup analysis we should always explain the pooled proportion estimate together with 95%CI or 95% PI. As you have pointed out, there are several places in the original manuscript where we were not clear enough or over-interpreted at the matter. We have made changes to these issues, detailed next.

Changes made:

Change 1:

In discussion, Limitation and footnote of Table 3, we highlighted the fact that the proportions do not add up to 100% and explained the reason by adding 'the proportions do not add up to 100%, because different studies are included for estimating each outcome, and sampling errors accounted for is partially dependent on the absolute number for each cause' (P41/901; Tab3, footnote; P52/R1233);

Change 2:

We had this description of trends over time in original manuscript, which over-interpreted the trends:

'For trauma, we noted a proportion of 23% (CI 20–27%) in the 1970-2000 subgroup, which increased to 29% (CI 21–39%) in the after-2000 subgroup, while asphyxia showed a rise from 72% (CI 68–75%) to 82% (CI 72–88%) across the same periods. This subgroup strategy accounts for substantial amount of heterogeneity from overall proportion estimates , denoting reliability of identified trends over time.'

It has been revised to (P44/R990):

'For trauma, we noted a proportion of 23% (CI 20–27%) in the 1970-2000 subgroup, which **turned** to 29% (CI 21–39%) in the after-2000 subgroup, while asphyxia showed a **change** from 72% (CI 68–75%) to 82% (CI 72–88%) across the same periods. This subgroup strategy accounts for substantial amount of heterogeneity from overall proportion estimates **(according to none to low I^2 and Cochran's Q test)**, denoting **time's role in explaining between-study heterogeneity**. '

Change 3:

In Limitations, we have further highlighted that we do not use normalization or multi-nominal modeling, and explained our rationale (P52/R1233); copied below for your convenience:

In our study, the proportion estimates of three causes do not add up to 100%. This is because different studies are included for estimating each outcome; the sampling errors accounted for are partially dependent on the absolute number for each cause; and each proportion is estimated separately. Trikalinos et al.[1] suggest using a multi-nominal model that enforces a constraint in estimating the pooled proportion that the sum of the pooled proportions should be 100%. However, this model assumes mutual exclusiveness of the outcomes. Our included studies vary in whether they reported all three causes, two causes, or a combination of multiple causes, which prevented us from adopting this approach. This also undermines the validity of normalizing the sum of the three estimates to 100%. Importantly, we believe this does not invalidate our proportion estimates and between-subgroup comparisons. Separate analyses, without imposing artificial trade-offs between causes, can account for heterogeneity of each cause more properly (especially when there are different numbers of studies for each cause). For this reason, our practice better fits one of the principal aims of the study, which is to explore the factor that explains the high heterogeneity in the overall proportion of estimates. Separately estimating each correlated outcome is also the practice of other meta-analyses with a design similar to ours [2,3]. As suggested by Barker et al.[4], for proportional meta-analyses, differences between subgroups can be compared and contrasted using a statistical test. This practice is also used in other meta-analyses with a design similar to ours [3]. In our study, the various categorizations of causes have been fully documented and the implications have been accounted for by our risk-of-bias analysis and fully discussed. Our subgroup comparisons were always made by referring to both the proportion estimates and statistical indicators (CI, PI, Cohrane's Q, etc.)

To sum up: in principle, we agree that proportions would be tidier and more intuitive if they summed to 100% – however, in practice we cannot necessarily expect this outcome because (a) there is sampling error, and (b) causes of death may not be reported exclusively.

2. Page 14/324 and 341: It should be stated if the p-value is double or single

sided

Response: Two-sided p-value has been stated. (P13/R317)

3. Page 24/Table: note § reads "for man". Shouldn't this be "male"?.

   Yes, it should've been "male". It's corrected now. We really appreciated careful attention to detail and your thorough review (P23, footnote 2)

4. Page 38/Fig 2.: Please clearly specify the events—trauma, asphyxia, and hypothermia—insections a) to c), as they are currently mentioned only in the main text.

   Response: Now they have been specified in Figure 2.

5. Page 39/799: what does "(??)" mean?

   It should have been a citation to Appendix 8. Sorry for any inconvenience caused. Now it has been updated to correct content (P36/R786).

6. Page 40/table 3: at least a normalizing constant should be applied to given subgroup proportions.

   As explained above, applying a normalizing constant is a good practice but does not apply to our study due to the nature of the data. Instead, to caution the reader, we highlighted this fact in multiple places listed above and also here in the footnote of table 3: 'the proportions of three causes under one subgroup do not add up to 100%, because different studies are included for estimating each outcome, and sampling errors accounted for is partially dependent on the absolute number for each cause'. We also corrected all the relevant over-interpretations as described above. (P37, footnote double-dagger)

7. Page 41/846: this "(PI 50%–1)" should read (PI 50–100%)

   Response: It has been corrected to 100%. (P38/R832)

8. Page 43/Table 4: the "(data time periods: 1997–1028)" is given. 1028 should be corrected.

   Response: It has been corrected to 2018 (P41: table 4 footnote).

9. Page 44/917: Given proportions are not consistent with fig.2. 80% (CI 74-85% and 21% (CI 17-25%) should be the correct ones.

   Response: Yes, it has been corrected now.(P41/R899)

10. Page 44/926: Results would change when using a normalizing constant.

    Response: As explained above, applying a normalizing constant is a good practice but does not apply to our study due to the nature of the data (P41/R900). Instead, to caution the reader, we highlighted this fact in multiple places listed above and also in the beginning of the paragraph. We also corrected over-interpretations of any trends. With these improvements and also considering the reasons we described in *c*, we believe this conclusion is still valid. (P41/R910)

11. Page 47/1013: Results would change when using a normalizing constant.

    Response: We clarified the observed heterogeneity explained might be attributable to diverse patterns within the post-2000 subgroup (reflected by wider 95%PI), rather than trends of PCAD (P45/R1008).

12. Page 48/1029 and 1043: Results would change when using a normalizing constant.

    Response: We corrected our over-interpretations of the trends (we referred to 95%CI to make valid interpretation) (P44/R988).

13. Page 50/1084: We have recently shown that long-term survival as well as overall survival improved in the last decades in Switzerland, due to a shift from organized to rescue by comrades, shorter burial times and improved medical treatment.

    Response: Thank you for sharing this very relevant study. This important evidence further grounds our points, and we included it in the discussion (P47/R1071) .

14. Page 54/1223: Results would change when using a normalizing constant.

    Response: We corrected this over-interpretation of trends and focused on time period's role in explaining heterogeneity, which has more valid grounding (P51/R1202).

Reviewer 3

1. The paper is dense and a very tough read if a general readership is desired. It is extremely long (55 pages of regular text), it reads more like a thesis/dissertation rather than a focused journal manuscript. For me, it was a tough read, and I mostly just commented on the things I know. The authors did a mathematical meta-analysis approach. I have no expertise on this approach, The authors seemed knowledgeable and their setup of biased verses unbiased looks good, but again I am no expert and won't comment in this review. I hope that there are other reviewers that can comment on these aspects. The flowchart summarizes their approach well but I am not sure it could be appealing to general Plos Global Public Health Review readers.

   Response:

   We have made fresh efforts to revise the work for brevity and clarity, cutting or re-wording throughout the manuscript.

   However, our paper is not intended for a general readership but for those who can understand the detailed methodological approaches, especially those methods we use for meta-analysis. A more accessible version of the findings has been published with a short paper at International Snow Science Workshop (ISSW) 24[5] (https://arc.lib.montana.edu/snow-science/item.php?id=3351). After publication of this manuscript, we intend to further publicize the

findings in the popular press, for example community-oriented magazines, to raise awareness.

In addition, we note that the paper is so extensive to ensure it is comprehensive, rigorous, transparent, and reproducible, which are essential for a high-quality meta-analysis. This approach aligns with standard practices, such as meta-analyses published in Cochrane, which sometimes have over 100 pages[6]. The length serves two purposes: (A) to provide an informative and actionable conclusion for most readers; (B) to offer detailed methods and analyses for researchers and practitioners needing to evaluate rigor or replicate the study. While reading abstract and/or conclusion address general readers' needs, the full text ensures transparency and scientific integrity for specialized audiences. When new evidence emerges, such integrity makes it possible for any interested researcher to reproduce and update the summary estimate based on the reported methods and results.

2. 1415 reports but why a much number for meta-analysis?

   Response:

   Following the procedures recommended by PRISMA guideline[7], we conducted a broad initial search to identify all potentially relevant studies. This was followed by a rigorous manual eligibility assessment, systematically evaluating the relevance and quality of each study to ensure that only those meeting our predefined criteria were included in the final analysis. Our initial search yielded 1,415 reports, of which 22 studies met the criteria for inclusion in the meta-analysis, while 37 studies met the criteria for qualitative review.

3. I am skeptical on the quality of some of the databases (electronically) used to search for avalanche death data. The authors should focus on official avalanche data from things like the USA Westwide Network and reported avalanche incident reports, and similar official/operation databases. Things like Academic Search Complete, SPORTDIscus, etc. may not have a true picture on whether it was officially a true avalanche scientifically. The authors provide little information on the geography of avalanche incidents. I am pleased they looked for non-English sources, but comprehensive good avalanche incident data is critical. For at least the United States (can be different in some international countries), it is well-known that backcountry skiers have a much different aspect on avalanche death issues than those in regular ski-areas (pretty much non-existent)

   Response:

   Meta-analyses constitute fundamental tools of the Evidence-Based Medicine (EBM) aiming at synthesizing outcome data from **individual trials or studies**[8]. Raw case reports that lack consistent reporting of events of interest, such as case reports from *avalanche.org* are not typically considered for inclusion since they are prone to selection/reporting bias, do

not allow for quality evaluation and statistical pooling and may over-represent rare events. For this reason, we followed PRISMA procedures[7] and searched a comprehensive set of databases of scientific publications for studies summarizing avalanche deaths, such as Academic Search Complete, SPORTDIscus, Medline, Embase, etc, rather than raw case reports.

Despite this, to produce a comprehensive meta-analysis, we did consider all possible data sources to enhance the rigor of our results. We achieved this asking domain experts with multiple language backgrounds to suggest studies and data sources that we failed to cover (P16/R377). In that phase, one of the experts suggested multiple official avalanche data source including SLF (www.slf.ch), ANENA (anena.org), avalanche accident reports from American Avalanche Association (avalanche.org) and Avalanche Canada (avalanche.ca). We fully evaluated these sources and found (a). they mostly do not have a consistent reporting of cause of avalanche death for all cases, and (b). the region and time period covered by these sources overlap with some of included studies with more rigorous reporting of methodology. We hence did not include the data in our analysis. These decisions are fully documented in an Apendix of screening details (P54/R1285 in manuscript; P17 in appendix; or http://osf.io/e3d5v).

Regarding the databases used, we acknowledge the variability in data quality across sources. However, we followed PRISMA procedures[7] to ensure good practices in systematic review and meta-analysis. To address quality concerns, we prioritized data verification through triangulation with official sources, where available. We also included gray papers with stats from official accident databases.

As for geographic considerations, we do include region as a subgroup analysis factor. Additionally, many studies mention the terrain as a relevant factor in avalanche fatalities. While we recognize differing perspectives on its influence, our inclusion is supported by existing literature.

4. The section on Weather and Snow doesn't consider detailed meteorological factors within a climate timeframe that could be important on avalanche fatalities. I wouldn't expect terrain to be a big factor in fatalities. On changes of factors through time on fatalities, I would like to see some clear visual(s) that show this over the years, but with the sketchy nature of most avalanche fatality data, especially in some remote areas in some cases, its hard to do.

   Response:

   We agree there should be detailed meteorological factors within a climate timeframe. However, because meta-analysis is a summary of existing evidence, our level of detail depends on the included studies. We did propose some climate-relevant assumptions, and point out they could have been verified if the included studies had reported more information on the matter (P50/R1167). We appreciate the suggestion to explore these in future analyses. This is now better expressed in the text: (P53/R1256)

'We are unable to conduct subgroup analyses on burial type, depth, and duration, and their impact on the proportions of causes of avalanche deaths, due to the lack of reporting of these features in included studies. In addition, some subgroups were now inspired by the meta-analysis. However, different types of groupings could be used in the future work. '

We agree with the comment on the need for clear visuals to depict changes over time. While our focus was on summarizing trends within the limitations of the data, future analyses can utilize these aggregations for investigating specific elements in the results.

Thank you for highlighting these points, which have allowed us to clarify our methodology and focus.

References

[1] Trikalinos TA, Olkin I. A method for the meta-analysis of mutually exclusive binary outcomes. Statistics in Medicine. 2008; 27(21):4279–4300.

[2] Law MR, Morris JK, Wald NJ. Use of blood pressure lowering drugs in the prevention of cardiovascular disease: meta-analysis of 147 randomised trials in the context of expectations from prospective epidemiological studies. Bmj. 2009; 338.

[3] Weidner J, Kern I, Reinecke I, Bathelt F, Manuwald U, Henke E, et al. A systematic review and meta-regression on international trends in the incidence of ulcerative colitis in children and adolescents associated with socioeconomic and geographic factors. European Journal of Pediatrics. 2024; 183(4):1723–1732.

[4] Barker TH, Migliavaca CB, Stein C, Colpani V, Falavigna M, Aromataris E, et al. Conducting proportional meta-analysis in different types of systematic reviews: a guide for synthesisers of evidence. BMC medical research methodology. 2021; 21:1–9.

[5] Guang R, Ahonen L, Pfuhl G, Cowley BU. DEATH OF BACKCOUNTRY WINTER-SPORTS PRACTITIONERS IN AVALANCHES–A SYSTEMATIC REVIEW AND META-ANALYSIS OF PROPORTION OF DEATH CAUSES;.

[6] Harvey L. Summaries of Cochrane Systematic Reviews: Making high-quality evidence accessible. Spinal Cord. 2018; 56(3):185–185.

[7] Page MJ, McKenzie JE, Bossuyt PM, Boutron I, Hoffmann TC, Mulrow CD, et al. The PRISMA 2020 statement: an updated guideline for reporting systematic reviews. bmj. 2021; 372.

[8] Papakostidis C, Giannoudis PV. Meta-analysis. What have we learned? Injury. 2023; 54:S30–S34.
