# Supplementary material for: Death of backcountry winter-sports practitioners in avalanches – A systematic review and meta-analysis of proportion of causes of avalanche death
Source: PLOS Glob Public Health. 2025 May 30;5(5):e0004551. doi: 10.1371/journal.pgph.0004551 (PMC12124587; doi:10.1371/journal.pgph.0004551)
Supplement: Attachment — Submitted filename: response_to_reviewers.pdf [file pgph.0004551.s015.pdf]

Response to reviewers

March 8, 2025

Reviewer 1

1. Some counting mistake has occurred: In line 889 the authors mention four results of this review: After the third, in line 892, the go on with Fifth line 894 and sixth line 895 without mentioning the fourth. Please correct

Response: Thank you for you careful review. We mistakenly indexed the fourth point as the fifth. We appreciate your attention to detail. Relevant texts have been revised accordingly in the manuscript (P44/R929 and R930).

Reviewer 2

1. please add the file MetaData2024.xlsx to the repository https://osf.io/ygza4/.

   Response: We have uploaded the requested file to OSF (https://osf.io/br8m5) and updated the manuscript accordingly (P15/R358). For clarity, we have renamed the file to "MetaData.xlsx". Additionally, we have updated the corresponding codes in OSF (https://osf.io/4c5ah) to correctly read the new file name.

   Response:

2. In line 753 on page 35/66 there are two question marks (??) in the text.

   Response: Thank you for your careful review. The issue was caused by a LaTeX syntax error that prevented the correct citation of Appendix S11. We have now corrected it (P38/R781).
