# Supplementary material for: Death of backcountry winter-sports practitioners in avalanches – A systematic review and meta-analysis of proportion of causes of avalanche death
Source: PLOS Glob Public Health. 2025 May 30;5(5):e0004551. doi: 10.1371/journal.pgph.0004551 (PMC12124587; doi:10.1371/journal.pgph.0004551)
Supplement: Attachment — Submitted filename: response_to_reviewers_auresp_2.pdf [file pgph.0004551.s016.pdf]

The authors provide a comprehensive review of the causes of death in avalanche accidents. A meta-analysis of 18 out of 26 eligible studies offers estimates on the proportions of trauma, asphyxia, and hypothermia-related fatalities. Subgroup analyses further refine these findings, offering deeper insights into the underlying factors and patterns of mortality.

This paper represents the most comprehensive review to date on the subject, offering a significant and valuable contribution to the field.

There are however some issues and concerns that should be addressed to improve the paper before publication.

Main concerns

Meta-analyses of categorical or ordinal data are typically conducted by treating the data as dichotomous [1]. As a result, the point estimates and confidence intervals apply to individual events, such as trauma, but are not suitable for providing a combined estimate across multiple events.

For example, on page 44/926, the proportions of trauma and asphyxia from 1970 to 2000 are reported as 23% and 72%, respectively. For the period after 2000, the proportions are 29% for trauma, 82% for asphyxia, and 2% for hypothermia, totaling 114%. To correct this, the proportions should be normalized so that their sum equals 100%. Applying a normalizing constant of 1/1.14 results in corrected proportions of 25% for trauma, 73% for asphyxia, and 2% for hypothermia, making these values comparable to those before 2000.

Since applying a normalizing constant can significantly alter the results, it is essential to use appropriate methods, as suggested in [2, 3, 4, 5], to obtain reliable combined proportion estimates. Alternatively, proportions should at least be normalized when comparing event proportions or subgroups to ensure accurate comparisons.

Minor comments

Page 14/324 and 341: It should be stated if the p-value is double or single sided

Page 24/Table: note § reads “for man”. Shouldn’t this be “male”?.

Page 38/Fig 2.: Please clearly specify the events—trauma, asphyxia, and hypothermia—in sections a) to c), as they are currently mentioned only in the main text.

Page 39/799: what does “(??)” mean?

Page 40/table 3: at least a normalizing constant should be applied to given subgroup proportions.

Page 41/846: this “(PI 50%‐1)“ should read (PI 50‐100%)

Page 43/Table 4: the “(data time periods: 1997‐1028)” is given. 1028 should be corrected.

Page 44/917: Given proportions are not consistent with fig.2. 80% (CI 74-85% and 21% (CI 17-25%) should be the correct ones.

Page 44/926: Results would change when using a normalizing constant.

Page 47/1013: Results would change when using a normalizing constant.

Page 48/1029 and 1043: Results would change when using a normalizing constant.

Page 50/1084: We have recently shown that long-term survival as well as overall survival improved in the last decades in Switzerland, due to a shift from organized to rescue by comrades, shorter burial times and improved medical treatment [6].

Page 54/1223: Results would change when using a normalizing constant.

[1] https://training.cochrane.org/handbook/current/chapter-10#section-10-7

[2] Avci, E. (2021). Bayesian approach to the meta-analysis of multi-category prevalence. Communications in Statistics - Simulation and Computation, 50(5), 1541–1559. https://doi.org/10.1080/03610918.2021.1887229

[3] Rebecca Graziani, Sergio Venturini, A Bayesian approach to discrete multiple outcome network meta-analysis, PLOS ONE, 10.1371/journal.pone.0231876

[4] Whitehead, A., Omar, R.Z., Higgins, J.P.T., Savaluny, E., Turner, R.M. and Thompson, S.G. (2001), Meta-analysis of ordinal outcomes using individual patient data. Statist. Med., 20: 2243-2260. https://doi.org/10.1002/sim.919

[5] Trikalinos, T.A. and Olkin, I. (2008), A method for the meta-analysis of mutually exclusive binary outcomes. Statist. Med., 27: 4279-4300. https://doi.org/10.1002/sim.3299

[6] Rauch S, Brugger H, Falk M, et al. Avalanche Survival Rates in Switzerland, 1981-2020. JAMA Netw Open. 2024;7(9):e2435253. doi:10.1001/jamanetworkopen.2024.35253
